# Light-Activated Metal-Dependent Protein Degradation: A Heterobifunctional Ruthenium(II) Photosensitizer Targeting New Delhi Metallo-β-lactamase 1

**DOI:** 10.1021/jacs.5c12405

**Published:** 2025-11-27

**Authors:** Lars Stevens-Cullinane, Thomas W. Rees, Calum Evans, Po-Yu Ho, Mika Kintzel, Yew Mun Yip, Ruoning Jia, Jonathan Bailey, Eleanor Clifford, Ruqaiya Alam, Sarah Maslen, Stephane Mouilleron, Adrien Pasquier, Ok-Ryul Song, Scott Warchal, Joanna Redmond, Michael Howell, Svend Kjær, Mark Skehel, Manuel M. Müller, Eachan O. Johnson, Maxie M. Roessler, Jeannine Hess

**Affiliations:** † Department of Chemistry, 4616 King’s College London, London, SE1 1DB, U.K.; ‡ The Biological Inorganic Chemistry Laboratory, 376570 The Francis Crick Institute, London, NW1 1AT, U.K.; § Systems Chemical Biology of Infection and Resistance Laboratory, The Francis Crick Institute, London, NW1 1AT, U.K.; ∥ Chemical Biology Science and Technology Platform, The Francis Crick Institute, London, NW1 1AT, U.K.; ⊥ Department of Chemistry, 4615 Imperial College London, London, W12 0BZ, U.K.; # Proteomics Science and Technology Platform, The Francis Crick Institute, London, NW1 1AT, U.K.; g Structural Biology Science and Technology Platform, The Francis Crick Institute, London, NW1 1AT, U.K.; h High Throughput Screening Science and Technology Platform, The Francis Crick Institute, London, NW1 1AT, U.K.; 9 Department of Chemistry, Department of Chemistry and Centre for Pulse EPR Spectroscopy (PEPR), 4615 Imperial College London, London, W12 0BZ, U.K.

## Abstract

Antimicrobial resistance (AMR) is a global health threat, yet, despite this, antibiotic drug discovery has stagnated. Most compounds entering the clinic represent already discovered classes, to which bacteria already display resistance. We urgently need novel therapeutics to address this. Targeted protein degradation, typified by proteolysis-targeting chimeras (PROTACs), is a promising approach that has already seen success in oncology. A significant hurdle faced by these methods, however, is the complexity inherent in recruiting the host cell’s proteolytic processes. We herein describe an approach where proteolysis is performed by a light-activated ruthenium complex, termed LAMP-D (**Light-Activated Metal-dependent Protein Degradation**), thus circumventing the need for ligase recruitment. This method allows precise spatiotemporal control of protein degradation and may be adapted to degrade other proteins of interest. In a proof-of-concept study, New Delhi metallo-β-lactamase 1 (NDM-1) was chosen as a target for LAMP-D. NDM-1 is employed by Gram-negative bacteria to hydrolyze β-lactam antibiotics and is considered one of the most clinically relevant β-lactamase targets due to its global prevalence. In *in vitro* assays, the complex **Ru1** demonstrated a greater than 100-fold improvement in NDM-1 inhibition on exposure to light (450 nm, 20 J cm^–2^). Detailed analyses by SDS-PAGE and mass spectrometry show that **Ru1** induces highly specific degradation of the protein adjacent to the active site. **Ru1** was shown to inhibit NDM-1 in *Escherichia coli* expressing NDM-1 and demonstrated a 53-fold improvement in meropenem MIC with light irradiation (450 nm, 60 J cm^–2^). Furthermore, the complex exhibited no toxicity toward mammalian cells.

## Graphical abstract

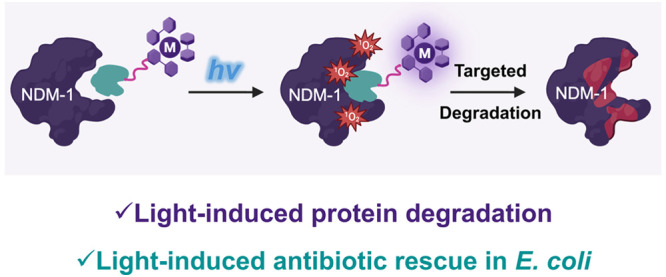

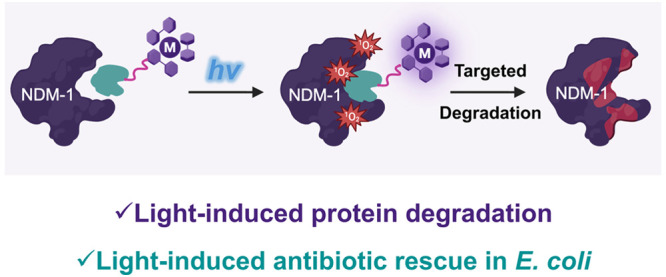

## Introduction

The rise of antimicrobial resistance (AMR) represents one of the most pressing public health challenges of our time. Recent estimates indicate that 4.7 million deaths were associated with AMR in 2021,^1^ a figure that is projected to climb to 8.2 million per annum by 2050. Despite this, progress in antibiotic research has slowed. In 2023, the world health organization identified 32 antibiotics in clinical development, which specifically tackle their list of priority pathogens.^2^ Of these 32 compounds, only 18 have evidence of activity against at least one of the critical Gram-negative pathogens. Furthermore, most drugs that progress to market represent classes of compounds that have already been discovered. These ‘me-too’ drugs typically have modes of action to which bacteria have already developed resistance and therefore cannot be used as long-term solutions.^3^ One resistance mechanism that has proven challenging to address is the production of β-lactamase enzymes, which hydrolyze and inactivate β-lactam antibiotics.^4^ New Delhi Metallo-β-lactamase 1 (NDM-1) is a broad spectrum metallo-β-lactamase (MBL) produced primarily by Gram-negative pathogens,^5^ and can hydrolyze nearly all known β-lactam antibiotics with high efficiency.^6^ Many inhibitors for NDM-1 have been reported in the literature since its identification in 2009,^7–10^ however, none are yet clinically available. Only one compound, Taniborbactam, has reached phase III clinical trials, which indicates the clinical potential for targeting NDM-1.^11^

Given the limited success in translating an NDM-1 inhibitor to the clinic, we were interested in exploring novel therapeutic modalities to address this challenge. An exciting new approach in the field of oncology is the use of small heterobifunctional molecules which modulate disease mechanisms through targeted protein degradation.^12^ The archetypal method is known as proteolysis-targeting chimeras (PROTACs).^13,14^ These bifunctional molecules bring a target protein close to an E3 ubiquitin ligase, triggering proximity-induced ubiquitination and subsequent proteasomal degradation. Despite their success, PROTAC development is complex, requiring simultaneous optimization of two ligand-protein interactions to achieve efficient ternary complex formation. Further challenges are also posed by varying expression levels of some of the widely used E3 ligases in different cancer subtypes or tissues, and the mechanisms leading to protein degradation are not yet fully understood.^15–17^ PROTACs use the eukaryotic intracellular degradation machinery and cannot directly be used in bacteria and other prokaryotes. The exception to this is the recently reported BacPROTACs, which hijacks the ClpCP bacterial proteasomal machinery of mycobacteria to elicit degradation of bacterial proteins.^18,19^ BacPROTACs have shown success in Gram-positive bacteria and mycobacteria, however, the challenge of targeting Gram-negative bacteria using protein degradation modalities remains unanswered. Developing novel therapeutic modalities which target these species is therefore crucial.^20,21^

Photodynamic therapy (PDT) is an approach with the potential to address some of these challenges. In PDT, a photosensitizer (PS) is activated by light and produces reactive oxygen species (ROS) which can cause highly localized and targeted damage.^22,23^ PDT has seen clinical use since the first PS was approved in the 1990s.^24^ Although one of the earliest reports of PDT was the inactivation of a microbe,^25^ PDT research and clinical usage in the modern era has mainly focused on cancer and skin disease treatment.^22,26^ Clinically approved PSs have also been tested in smaller studies in patients for a wide array of diseases including fungal, viral, and bacterial infections.^26^ Use of phototherapy to treat bacterial infection (antimicrobial PDT, aPDT) has several key advantages. Most antibiotics are toxic in high doses, and extended courses can have off-target toxicity.^27,28^ In addition, research continues to reveal the importance of the microbiome in health and disease. Treatment with antibiotics can harm commensal bacteria which causes well documented secondary negative effects such as dysbiosis.^29,30^ In PDT, photoactivation allows pinpoint control of the timing and location of toxicity, preventing off-target effects. While the precise control afforded by light activation is an advantage of PDT, it is also a limitation. The visible light (400–700 nm) typically used in PDT penetrates tissues poorly allowing therapy only in areas accessible to light sources and on the surface of tissue. This makes phototherapy most suited to skin and dental disease treatment.^26,31^ However, effective treatment of bladder cancer,^32^ and *Helicobacter pylori* infection in the stomach for example demonstrate that optical probes can allow treatments beyond the surface of the body.^33^ Furthermore, major causes of nosocomial infections with serious clinical outcomes result from bacteria and biofilms on surfaces and implants such as catheters.^34,35^ Effective methods for the sterilization of these is urgently needed, and tissue penetration is not a restriction.

An approach related to both PDT and PROTACs is chromophore assisted light inactivation (CALI). CALI was first introduced in the late 1980s,^36^ whereby a photosensitizer dye, malachite green, was conjugated to streptavidin and exhibited the selective inactivation of biotinylated proteins upon binding and light activation. Most of the work on CALI in subsequent years used organic based dyes such as fluorescein,^37,38^ or ROS-producing proteins, such as KillerRed,^39^ SuperNova,^40^ and mini ‘singlet oxygen generator’ (miniSOG),^41^ to act as photosensitizers. The Kodadek group was the first to use a metal-based PS for CALI, a ruthenium­(II) polypyridyl complex, which was found to be a more effective PS than fluorescein.^42^ Subsequent work by the Kodadek group developed two peptoid-ruthenium conjugates in which a ruthenium polypyridyl complex was attached to two different peptoid targeting moieties. These peptoids targeted the complex to the vascular endothelial growth factor receptor 2 (VEGFR2), as well as Rpt4, which is one of the ATPases in the 26S proteosome. Both peptoid-ruthenium conjugates exhibited highly improved IC_50_ values against their respective targets through photoinduced inactivation.^43^ More recent work using ruthenium­(II)-based PS-conjugates have explored targeting the mitochondria, mitochondrial guanine quadruplexes,^44,45^ carbonic anhydrase IX,^46^ and cereblon.^47^

Metal polypyridyl complexes are well-known to include potent PSs, with advantageous properties for oncological biomedical applications.^48,49^ Due to their long-lived triplet excited states they exhibit efficient generation of reactive oxygen species (ROS) as well as strong phosphorescence, which enables direct imaging of the complexes *in cellulo*. They have therefore seen widespread use in approaches such as PDT and photoactivated chemotherapy (PACT).^32,50–56^ For example highly potent ruthenium polypyridyl complexes have been developed by the McFarland group, with one complex (TLD-1433) currently in phase II clinical trials for the treatment of bladder cancer.^57^ Ru polypyridyl complexes are therefore highly promising for antimicrobial phototherapy.^58^

Herein we present an alternative strategy to address challenging targets in Gram-negative bacteria, termed Light Activated Metal-dependent Protein Degradation (LAMP-D) (Figure 1). In this heterobifunctional approach, a metal complex which produces ROS upon light activation, is tethered to a targeting vector specific for a protein of interest (POI).^46,47^ Unlike PROTACs, which require complex protein–protein interactions to form an active ternary complex, this strategy relies on the interaction of a relatively small metal complex with the protein target, facilitating design and optimization. Light activation allows instant and precise control of ROS production, and due to the short lifetime (τ_Δ_) and small diffusion radius (*d*) of the generated reactive radicals (e.g., ^1^O_2_ τ_Δ_ ≈ 3.5 μs and *d* ≈ 150 nm), the biomolecule of interest is targeted selectively and off-target effects are minimized.^59^ Finally, with the correct choice of ligands and metal center, complexes with inherent phosphorescence can be leveraged to enable direct imaging of the molecules localization in cells or tissues via light microscopy.^60^ Previous work in this field has, like PROTACs, focused largely on oncology targets.^46,47^ However, this approach does not depend on endogenous protein-degradation machinery and can therefore be translated for use in Gram-negative bacteria.

**Figure 1:**
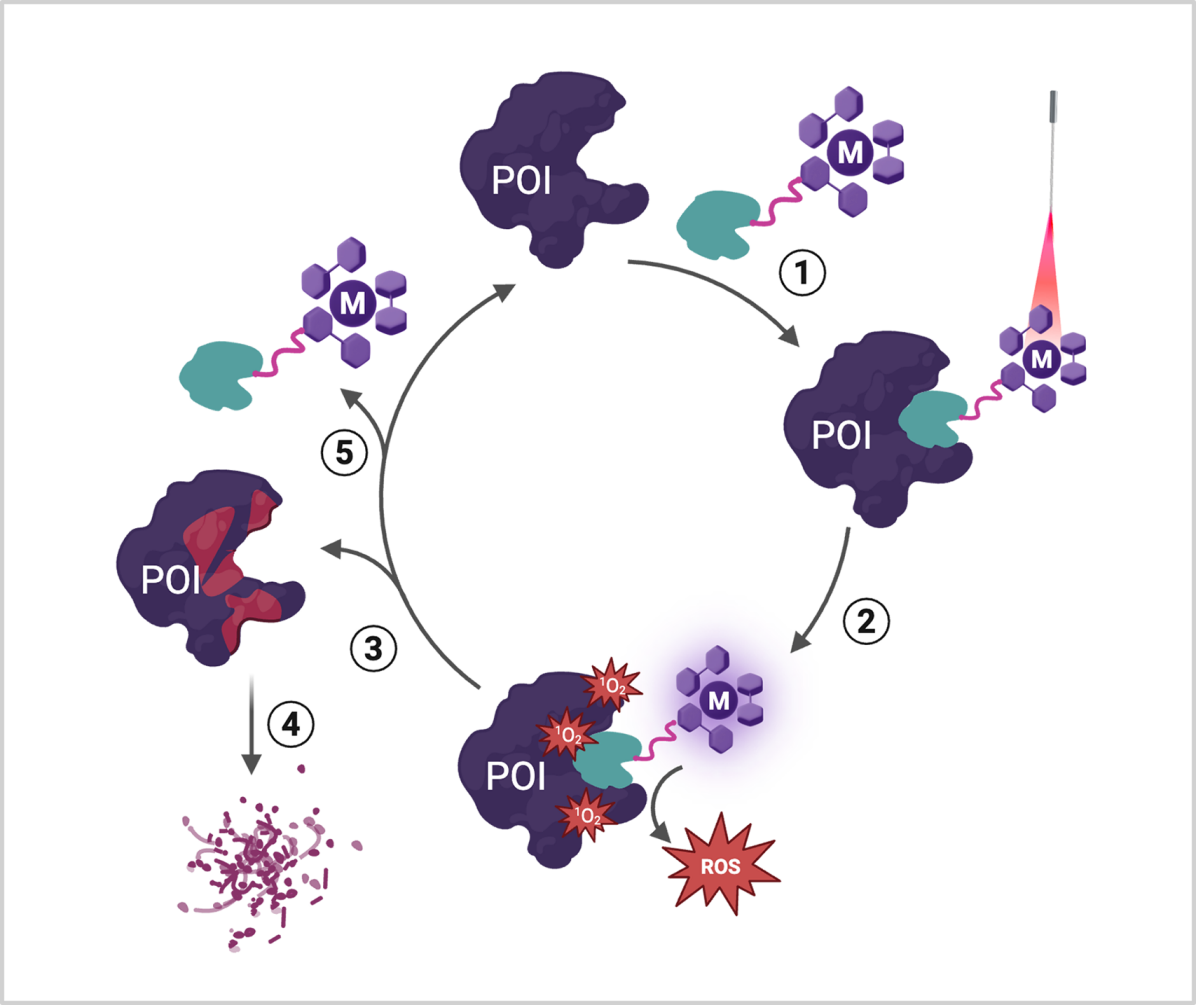
Light activated targeted protein degradation with a heterobifunctional metal complex. 1) LAMP-D molecule binds to the POI; 2) irradiation with light produces ROS in proximity to the POI; 3) the ROS produced by the LAMP-D irreversibly modifies and damages the POI; 4) degradation of the POI; 5) LAMP-D is released and may then bind to another POI to continue the cycle.

Herein we describe the first metal-based bacterial protein-targeted photosensitizer, **Ru1**, which binds to and photocatalytically inhibits NDM-1. We have shown that **Ru1** induces efficient and highly specific degradation of the enzyme *in vitro*. **Ru1** was also shown to both inhibit NDM-1 and rescue antibacterial efficacy in NDM-1 expressing *Escherichia coli*. This approach represents an innovative technology for addressing AMR in Gram-negative bacteria.

## Results and Discussion

### Metal Complex Design, Synthesis and Characterization

In the design of the heterobifunctional complex **Ru1** (Figure 2), we endeavored to find an NDM-1 inhibitor that 1) binds to the enzyme with high affinity, 2) allows further modification to the chemical structure without severely diminishing binding affinity and 3) is synthetically tractable to allow rapid access to the complex. The NDM-1 inhibitor 4-(3-amino-phenyl) dipicolinic acid (**N1**, Figure 2) was previously reported by Chen et al.^61^ and was found to bind to NDM-1 via interaction with the two active site Zn­(II) ions. Rather than sequestering Zn­(II), as observed for some inhibitors,^62^ **N1** forms a ternary structure between NDM-1 and the Zn­(II) ions. This was an important consideration when designing a targeting vector, as sustained proximity of the photosensitizer (PS) to the protein of interest (POI) is a necessity. Furthermore, structure activity relationship (SAR) profiling of **N1** revealed tolerance for modification of the amino group,^61^ therefore providing an ideal exit vector for attachment of our PS.

**Figure 2:**
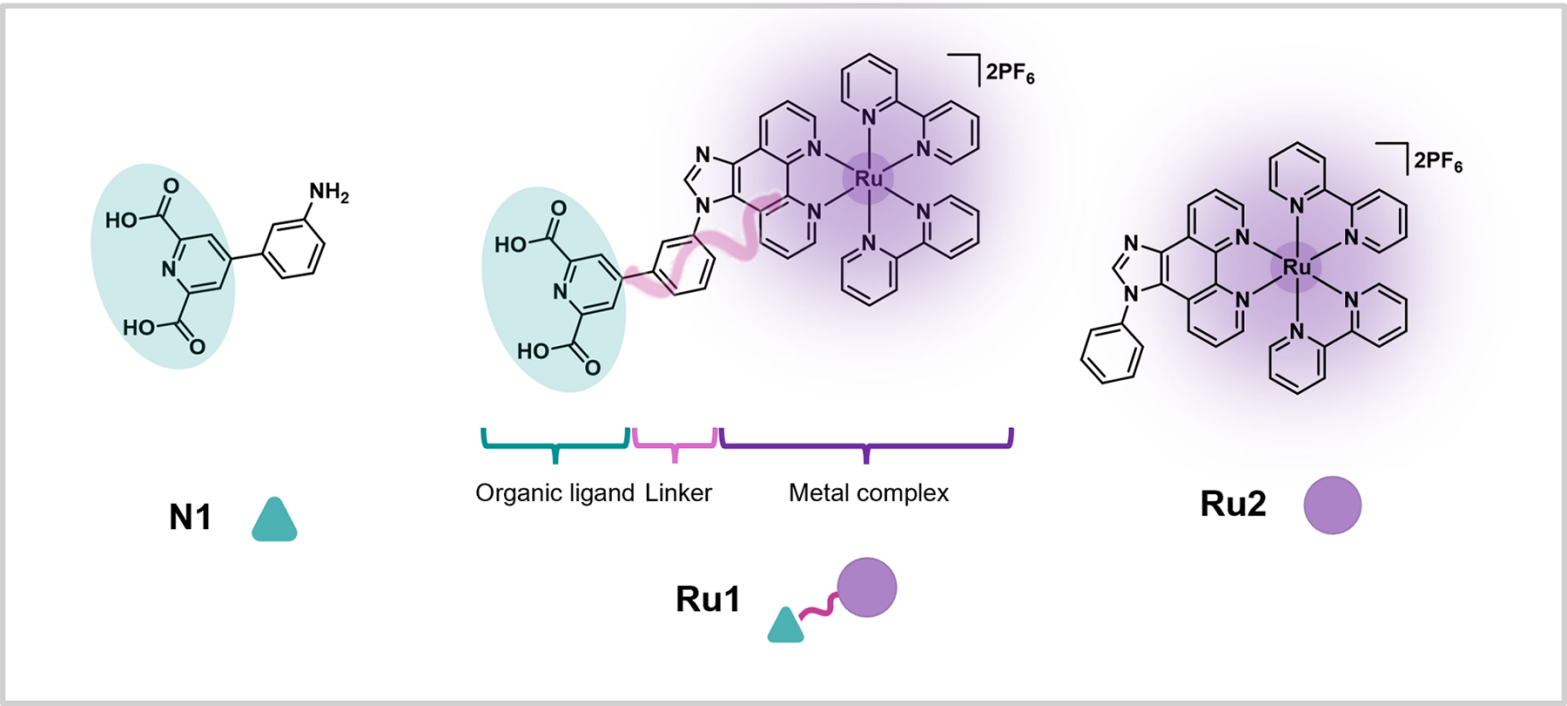
Design of metal complex **Ru1** as a targeted photocatalytic degrader of NDM-1. **Ru1** consists of an organic ligand for NDM-1 bound via a linker to a photosensitizer capable of producing ROS upon light irradiation. **N1** is the organic ligand control (represented here as the freebase form), while **Ru2** is the photosensitizer control without the targeting vector.

Due to the clinical advancement of these systems, we chose Ru­(bpy)_2_(**L**) as the core PS, where (**L**) denotes a N^N coordinating ligand to which the targeting vector is attached. Utilizing the ring closing synthesis used to great advantage in the synthesis of TLD-1433, we envisioned **L** as an imidazo­[4,5-*f*]­[1,10]­phenanthroline, which can be easily assembled to link the targeting vector to the PS core. This core ligand structure has seen widespread usage in PSs due to the ease of derivatization and positive impact on absorption and therefore PS activity.^55,63^

Taking these factors into consideration, the first step in the synthesis was the Suzuki–Miyaura coupling between dimethyl 4-chloropyridine-2,6-dicarboxylate and 3-aminophenyl-boronic acid to provide the diester **1** (Scheme 1). This was utilized as the intermediate for subsequent attachment to the PS. To synthesize **N1·HCl**, hydrolysis of the diester **1** was achieved via treatment with NaOH, followed by acidic work up. Cyclisation reaction between the diester **1** and complex **2** was performed. Partial de-esterification was observed during the reaction, and therefore saponification with LiOH was included as part of the workup to directly access **Ru1**. The metal complex control **Ru2** was synthesized by analogous cyclization of aniline with **2**. All compounds were fully characterized by NMR spectroscopy, LC-MS and HRMS. Chromatographically determined LogD values at pH 7.4 (chromLogD) were measured for **N1**, **Ru1**, and **Ru2** (Table S1).^64^ It should be noted that although **Ru1** was isolated as the PF_6_ salt, in buffer and media at pH 7.4 we expect the complex to be overall neutral due to deprotonation of the carboxylic acids.

**Scheme 1:**
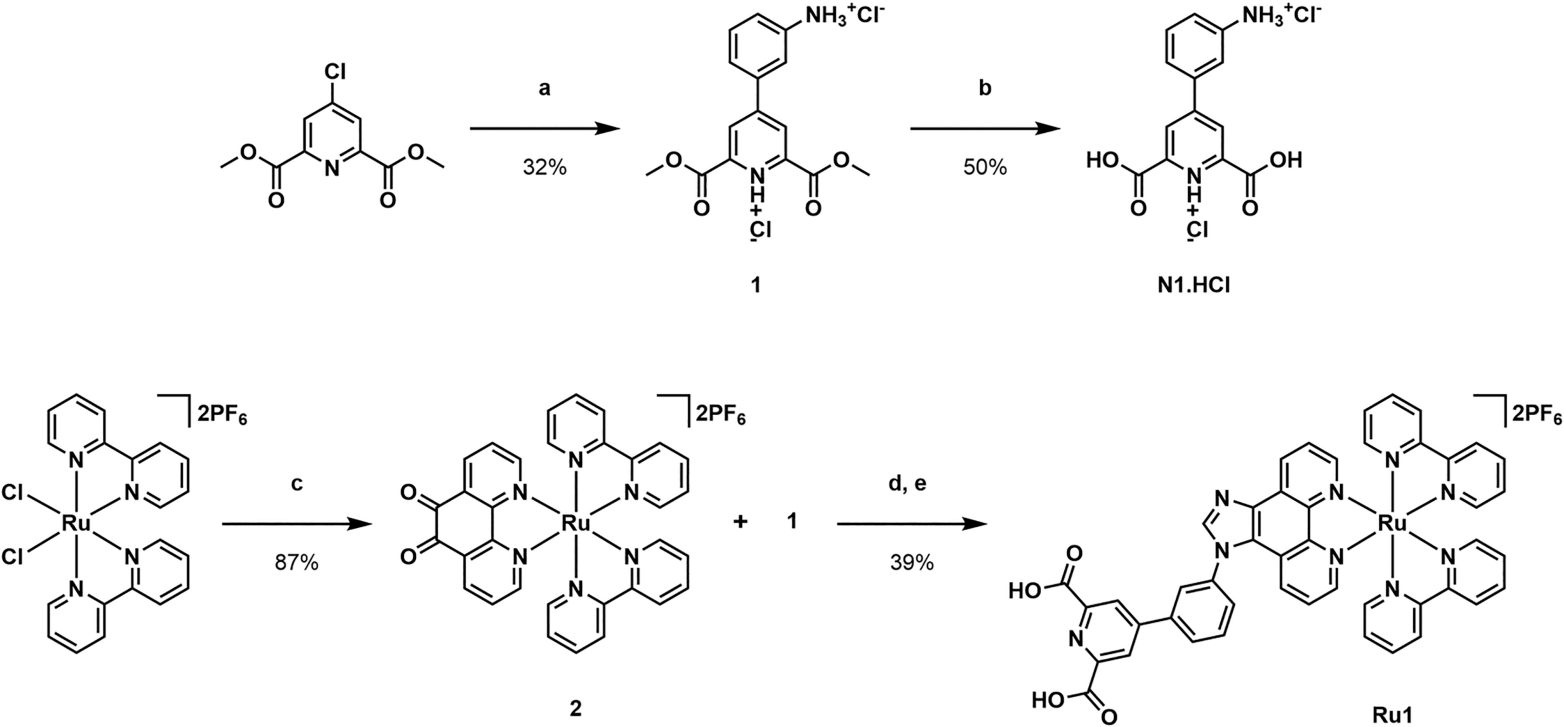
Synthesis of **N1·HCl** and **Ru1** ^a^

a a) Dimethyl 4-chloropyridine-2,6-dicarboxylate (1.0 equiv), 3-aminophenyl-boronic acid (1.6 equiv), K_3_PO_4_ (3.0 equiv), tetrakis­(triphenylphosphine)­palladium(0) (0.15 equiv) 1,4-dioxane, 85 °C, 18 h; b) (i) NaOH (5.0 equiv), THF, rt, 1 h, (ii) HCl, H_2_O; c) (i) 1,10-phenanthroline-5,6-dione (1.0 equiv), ethanol:H_2_O (95:5), 80 °C, 3 h, (ii) KPF_6_ (10 equiv), H_2_O; d) **1** (1.0 equiv), formaldehyde (1.1 equiv), NH_4_OAc (20 equiv), acetic acid, 120 °C, 4 h; e) LiOH·H_2_O (10 equiv), acetone:H_2_O (1:1), 90 min, (ii) KPF_6_ (10 equiv), H_2_O.

### Ru1 and Ru2 Are Highly Efficient Photosensitizers

To ensure that the complexes could act as effective PSs and oxidatively damage NDM-1, their photophysical properties were investigated. The UV–Vis spectra of both **Ru1** and **Ru2** (10 μM) were measured at pH 7.4 in phosphate buffered saline (PBS) and exhibit a broad absorption band from 350–560 nm with an absorbance maximum at 456 nm, indicative of the metal–ligand charge transfer (^1^MLCT) band for Ru­(II) complexes (Figure S2).^49^ **Ru1** and **Ru2** have near identical excitation and emission spectra. The excitation spectra align with the absorption data with MLCT based excitation maxima at 456 nm. Both complexes display emission maxima at 621 nm in PBS (Figure 3a, Figures S3 and S4).

**Figure 3:**
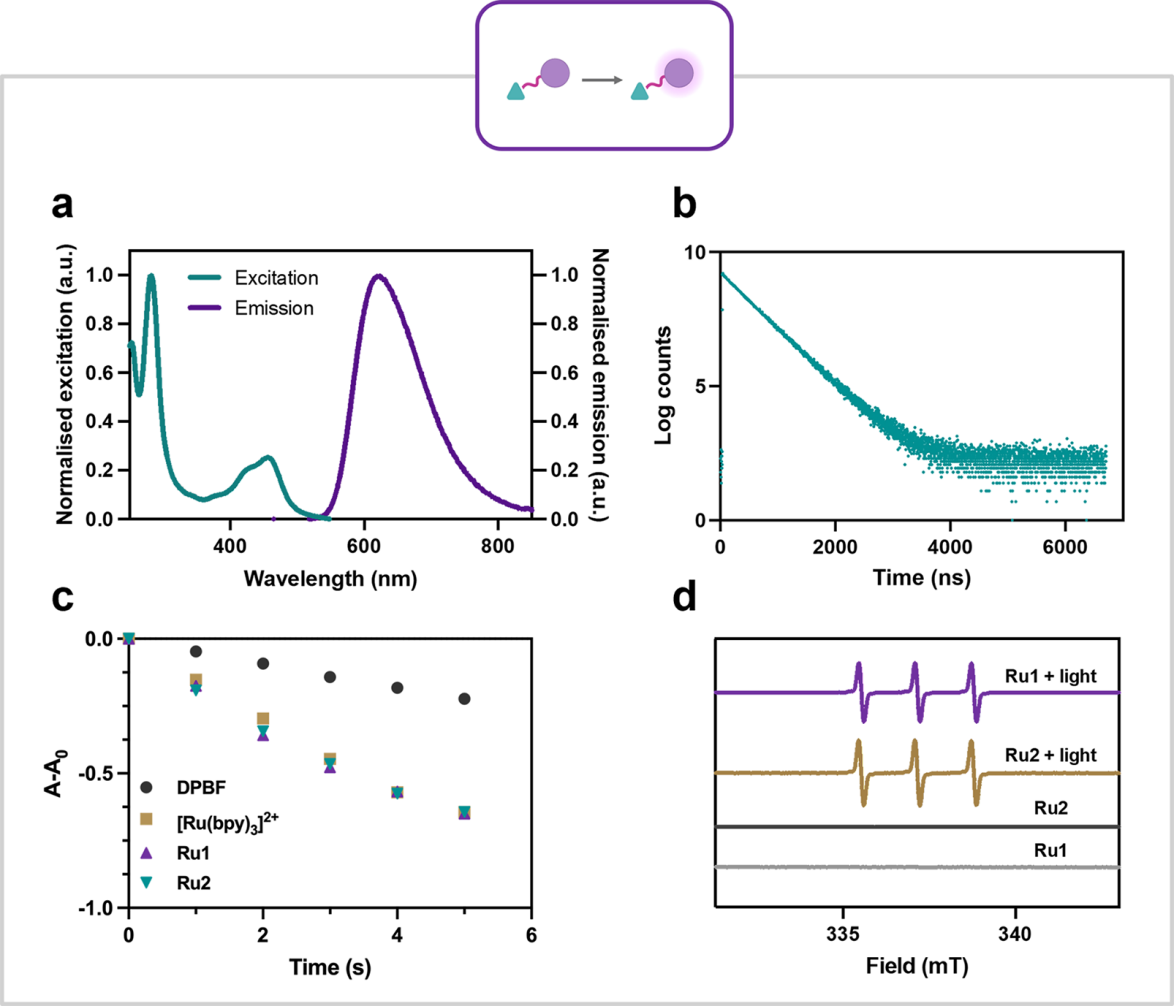
**a**: Excitation and emission spectrum of **Ru1** in PBS. **b**: Lifetime of the excited state of **Ru1** = 481 ns in PBS. **c**: Photooxidation of DPBF used to quantify the ^1^O_2_ quantum yield of **Ru1** (Φ_Δ_ = 0.89) and **Ru2** (Φ_Δ_ = 0.87) in oxygenated methanol. **d**: EPR spectra of **Ru1** and **Ru2** (1 mM) and 2,2,6,6-tetramethylpiperidine (20 mM) in methanol in the presence and absence of light, indicating the production of ^1^O_2_ upon irradiation.

To ensure stability toward light in aqueous media and at physiological pH, **Ru1** and **Ru2** were assessed by UV–Vis in PBS with light exposure (450 nm, 0.96 J cm^–2^ s^–1^). Both complexes were stable up to and including 23 J cm^–2^ (Figure S5). The lifetime of the excited state is one of the key factors which affect ROS generation.^65^ The luminescence lifetimes of **Ru1** and **Ru2** were therefore measured in PBS and found to be 481 and 463 ns, respectively, similar to the archetypal [Ru­(bpy)_3_]­Cl_2_ (510 ns) (Figure 3b).^66^ These lifetimes are considerably longer than organic photosensitizers such as the FDA approved porfimer sodium (14 ns) and protoporphyrin IX (13 ns) demonstrating the advantageous properties of ruthenium based PSs.^67^ Electron paramagnetic resonance (EPR) spectroscopy was performed with the spin traps 2,2,6,6-tetramethylpiperidine (TMP) and 5,5-dimethyl-1-pyrroline *N*-oxide (DMPO) to investigate the types of ROS produced by **Ru1** and **Ru2**. A strong and distinctive triplet EPR signal was observed for both **Ru1** and **Ru2** upon irradiation in the presence of the singlet oxygen (^1^O_2_) trap TMP (Figures 3d and S6). No signal was observed with the ^•^OH radical spin trap DMPO (Figure S7), indicating that ^1^O_2_ is the primary form of ROS produced.^68^ To quantify the generation of ^1^O_2_ produced by the complexes, 1,3-diphenylisobenzofuran (DPBF) was used as a ^1^O_2_ scavenger. The change in absorbance of DPBF in the presence of **Ru1** and **Ru2** in oxygenated methanol was measured with increasing light dosage. This data in comparison to the control ([Ru­(bpy)_3_]­Cl_2_) allows calculation of the ^1^O_2_ quantum yields (Φ_Δ_) (Figure 3c). The Φ_Δ_ of **Ru1** and **Ru2** were measured to be 0.89 and 0.87, respectively, which is comparable to ([Ru­(bpy)_3_]­Cl_2_) (Φ_Δ_ = 0.87) (Figure S8 and Table S2).^69^ The long-lived excited state and resulting high ^1^O_2_ quantum yields contribute to complexes **Ru1** and **Ru2** acting as highly efficient photosensitizers (Figure S9 and Table S3). Furthermore, the near-identical photophysical properties observed between compounds **Ru1** and **Ru2** validate the choice of **Ru2** as a control for further studies.

### Ru1 Binds to NDM-1

To investigate the binding of **Ru1** to NDM-1, differential scanning fluorimetry (DSF) was employed. Both compounds **Ru1** and **N1** destabilize the protein in a dose-dependent manner (Figure S10) suggesting strong binding to the protein. The control complex **Ru2** had no effect on the melting temperature of the protein (Table 1).

**Table 1:** Change in Melting Temperature (Δ*T*_m_) of NDM-1 upon Incubation with Compounds **N1**, **Ru1** and **Ru2**

Concentration (μM)	Δ*T*_N1_ (°C)	Δ*T*_Ru1_ (°C)	Δ*T*_Ru2_ (°C)
63	–8.8	–13	0.0
16	–3.4	–6.8	0.0
4	–0.6	–3.9	0.0
0	0.0	0.0	0.0

To measure an apparent K_d_ from the DSF data, the change in melting temperature (Δ*T*) was plotted against compound concentration, providing *K*_d,app_ values of 35 and 7.4 μM for **N1** and **Ru1**, respectively (Figure S11). These results show that **Ru1** successfully binds to NDM-1 and can be employed for proximity induced degradation.

### Ru1 Exhibits Targeted and Light Enhanced Inhibition of NDM-1

To determine the ability of complex **Ru1** to inhibit NDM-1, a chromogenic substrate assay was employed using the β-lactam nitrocefin. The hydrolysis product of nitrocefin is highly colored and therefore enables the direct spectroscopic monitoring of NDM-1 activity (Figure 4a).^70^ In the dark, it was found that **N1** inhibited NDM-1 with an IC_50(dark)_ of 3.2 ± 0.080 μM (Figure S13), while **Ru1** inhibits the enzyme with an IC_50(dark)_ of 23 ± 1.4 μM. Light irradiation of **N1** showed no enhancement of potency as expected.

**Figure 4:**
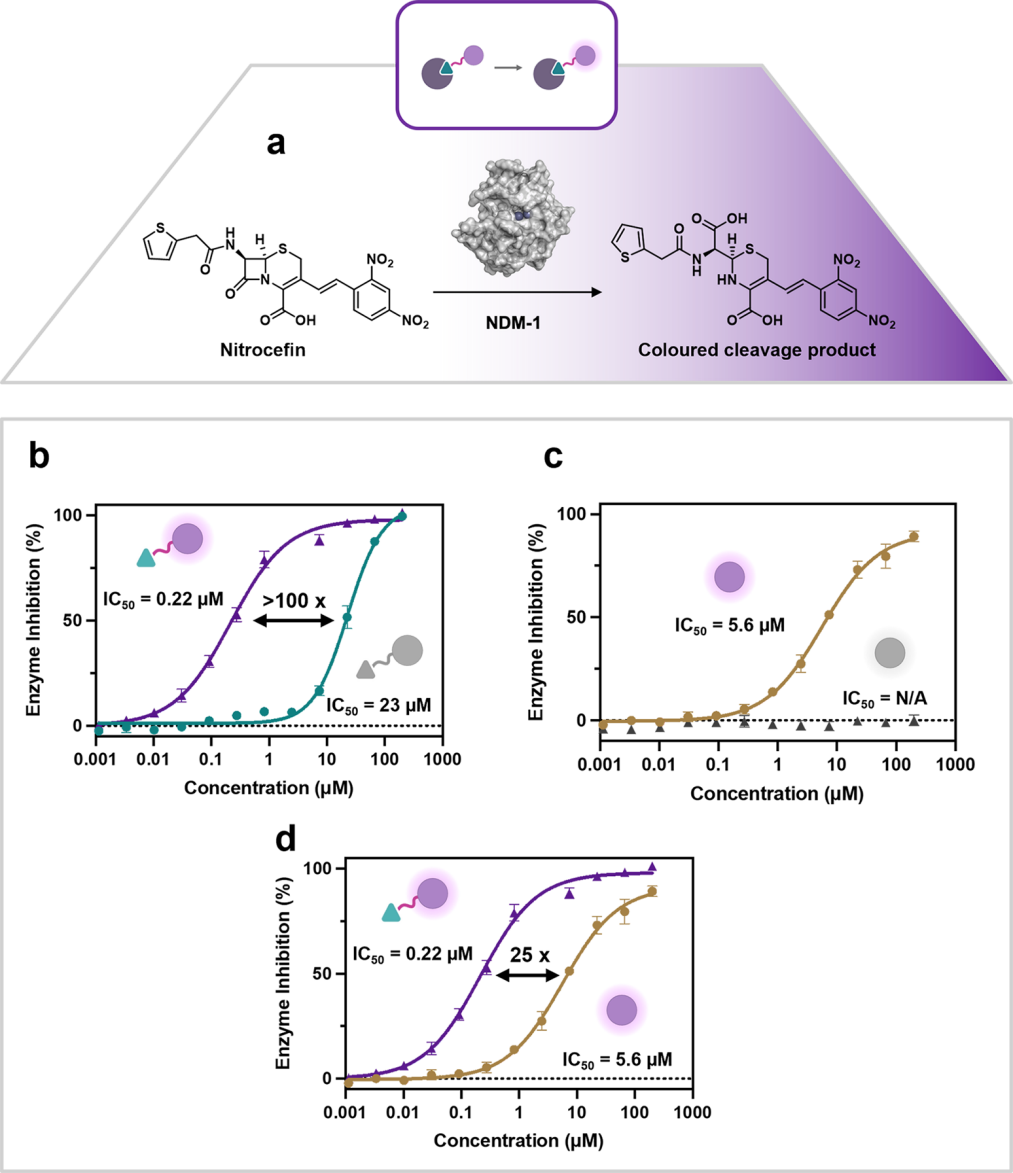
Chromogenic inhibition assay with NDM-1 and nitrocefin. **a:** On cleavage of the lactam ring of nitrocefin by NDM-1 a distinct color change occurs with absorption maximum at 486 nm. **b: Ru1** inhibits NDM-1 in the dark (23 ± 1.4 μM) and with light irradiation a 105-fold improvement in inhibition to 0.22 ± 0.020 μM is observed. **c: Ru2** inhibits NDM-1 in the light (IC_50_ = 5.6 ± 0.50 μM), but no inhibition is observed in the dark. **d:** Replotted data from b and c showing that **Ru1** displays 25-fold stronger inhibition of NDM-1 (IC_50_ = 0.22 ± 0.020 μM) compared with the untargeted control **Ru2** (IC_50_ = 5.6 ± 0.50 μM), when irradiated with light. Error bars represent standard deviation with *n* = 3.

When compound **Ru1** was incubated with enzyme and irradiated (450 nm, 20 J cm^–2^), a >100-fold improvement in potency was achieved compared to the dark conditions (IC_50(light)_ = 0.22 ± 0.020 μM) (Figure 4b). This result in combination with the photophysical data suggests that the light induced production of ROS causes significant damage to the protein with subsequent inhibition of the enzymatic activity. The next step was to investigate whether this inhibition was due to the synergistic effects of inducing proximity of the complex to the protein via attachment of the NDM-1 inhibitor **N1**. To test this hypothesis, the nontargeted control complex **Ru2** was incubated with NDM-1. In the dark this complex showed no inhibition, whereas irradiation of **Ru2** (450 nm, 20 J cm^–2^) inhibited the enzyme activity with an IC_50(light)_ = 5.6 ± 0.50 μM (Figure 4d). This was approximately 25-fold less potent than the targeted complex **Ru1** (Figure 4c). Literature studying the structure of NDM-1 and related MBLs, shows that the surface of NDM-1 is negatively charged.^71^ This could be responsible for some nonspecific electrostatic interactions with **Ru2**, contributing in part to the light-induced inhibition observed.

The organic inhibitor **N1** has been previously shown to exhibit selectivity for NDM-1 over other Zn­(II)-containing metalloproteins.^61^ To investigate whether our targeted complex **Ru1** indiscriminately binds other Zn­(II) containing proteins, we tested the complex against the clinically relevant Zn­(II)-containing metalloprotein histone deacetylase 1 (HDAC1). **Ru1** shows no off-target inhibition of HDAC1, demonstrating the complex does not inhibit all Zn­(II) containing proteins (Figure S15).

### Ru1 Effectively Degrades NDM-1

We further analyzed the effect of our metal complexes **Ru1** and **Ru2** on NDM-1 by SDS-PAGE and mass spectrometry in the presence and absence of light. Treatment of NDM-1 with up to 100-fold excess of **Ru1** in the dark showed very little effect on the monomeric band of NDM-1 (Mw_(NDM‑1)_ = 25 576 Da). However, even in strictly dark conditions, and at high concentrations of **Ru1**, a small amount of dimerized NDM-1 is observed (Figures S16 and S17). We speculate that there may be some oxidative cross-linking of monomers occurring with very low concentrations of ROS. This phenomenon is absent in the dark samples for the untargeted control complex **Ru2**, indicating that the proximity induced by the targeting moiety enhances this effect. This observation corroborates previous reports that NDM-1 can exist as both monomeric and dimeric forms in solution.^72^ When irradiated with light (450 nm, 20 J cm^–2^), the samples containing **Ru1** show prominent blurring of the monomeric band of NDM-1, as well as formation of a cross-linked NDM-1 dimer band. Samples containing **Ru2** exhibit a similar effect to the monomeric band of NDM-1 at high concentrations, however, this is not as prominent. When irradiated with a higher dose of light (450 nm, 60 J cm^–2^) cleavage of the protein into smaller peptides is observed (Figure S17).

To investigate whether **Ru1** selectively modifies and damages NDM-1 in the presence of other proteins, NDM-1 and bovine serum albumin (BSA) were incubated with **Ru1** in the presence and absence of light. Densitometry of the SDS-PAGE bands was used to measure the decrease in band intensities with increasing concentrations of **Ru1** (Figure 5c and d). Both proteins were unaffected in the dark, whereas with light irradiation (450 nm, 20 J cm^–2^), only NDM-1 was selectively modified and degraded. When this experiment was carried out using the untargeted complex **Ru2**, both NDM-1 and BSA band intensities were diminished upon light irradiation, albeit to a lesser extent than observed for NDM-1 in the presence of **Ru1** (Figures S18/S19). In addition, intact mass spectrometry was performed on the samples prepared for SDS-PAGE. These experiments show that in the absence of **Ru1**, NDM-1 is intact under both dark and irradiated conditions. With a high excess of compound (100 equiv) but without irradiation, no change is observed to the protein (Figure 5a). However, when irradiated (450 nm, 20 J cm^–2^) in the presence of only one equivalent of **Ru1**, considerable modification to the protein is observed, exemplifying that only a small quantity of **Ru1** is required to modify and damage NDM-1 (Figure 5b).

**Figure 5:**
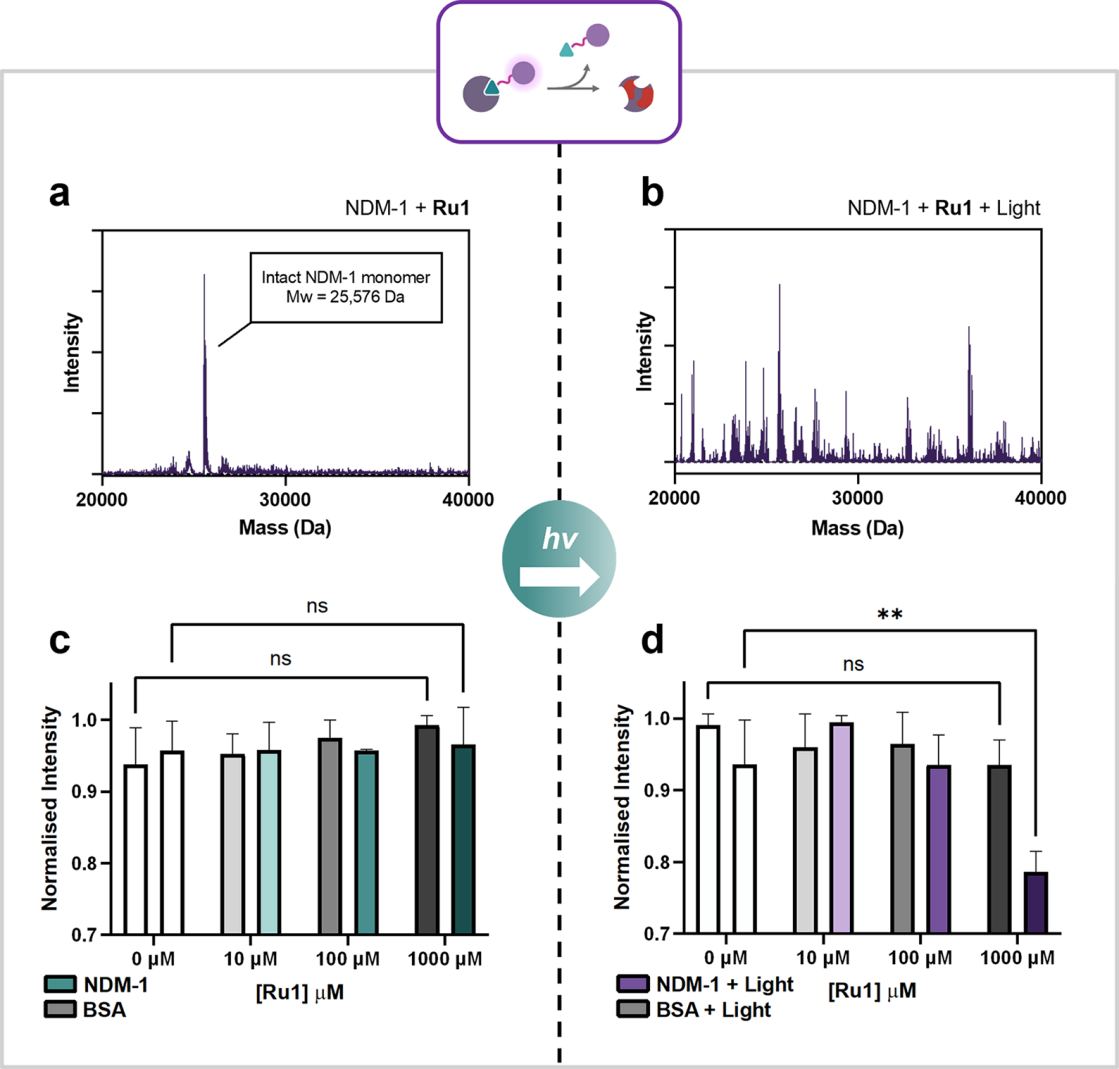
**a:** Deconvoluted intact mass spectrum of NDM-1 following incubation with **Ru1** (1 equiv) in the dark (Mw_(NDM‑1)_ = 25,576 Da). **b:** Deconvoluted intact mass spectrum of NDM-1 following incubation with **Ru1** (1 equiv) under light irradiation (450 nm, 20 J cm^–2^). **c:** Results of SDS-PAGE densitometry analysis of a mixture of NDM-1 and BSA treated with **Ru1** (0–1000 μM) in the dark. **d:** Results of SDS-PAGE densitometry analysis of a mixture of NDM-1 and BSA treated with **Ru1** (0–1000 μM) under light irradiation (450 nm, 20 J cm^–2^). Selective degradation of NDM-1 over BSA is exhibited between 0 and 1000 μM. Error bars represent standard deviation with *n* = 3. ns (not significant) = *P* > 0.05, ** = *P* ≤ 0.01.

Following this, liquid chromatography–mass spectrometry (LC-MS) was employed to analyze small modifications to the protein which SDS-PAGE may not reveal. Several gel bands from the SDS-PAGE were excised and analyzed by in-gel tryptic digestion followed by LC-MS/MS (Figures S28–S34), revealing that each band contained NDM-1 and there was a clear correlation between the light treated samples and decreased amino acid sequence coverage. ROS produced by **Ru1** modifies the protein, resulting in undetectable amino acid sequences. The amino acid sequence Met126–Lys181 was consistently modified in each light treated sample with **Ru1** (Figure 6b, purple). This is a solvent exposed region of the protein adjacent to the active site residues His120, His122, Asp124 and His189 (Figure 6b, gold). This further confirms that the targeting vector of **Ru1** is binding in the active site, with the PS core situated extending outward from to the binding pocket (Figure 6a and b). Within the damaged region of the protein is the Ω-loop, the function of this particular loop in MBLs has yet to be studied extensively. However, the corresponding loop in serine β-lactamases aids substrate binding, and mutations in this loop enables active site expansion and substrate spectrum extension.^73–77^ To visualize the potential binding pose and interactions of **Ru1** with NDM-1, and further rationalize the LC-MS data, molecular docking of **Ru1** to the NDM-1 active site was performed using MetalDock.^78^

**Figure 6:**
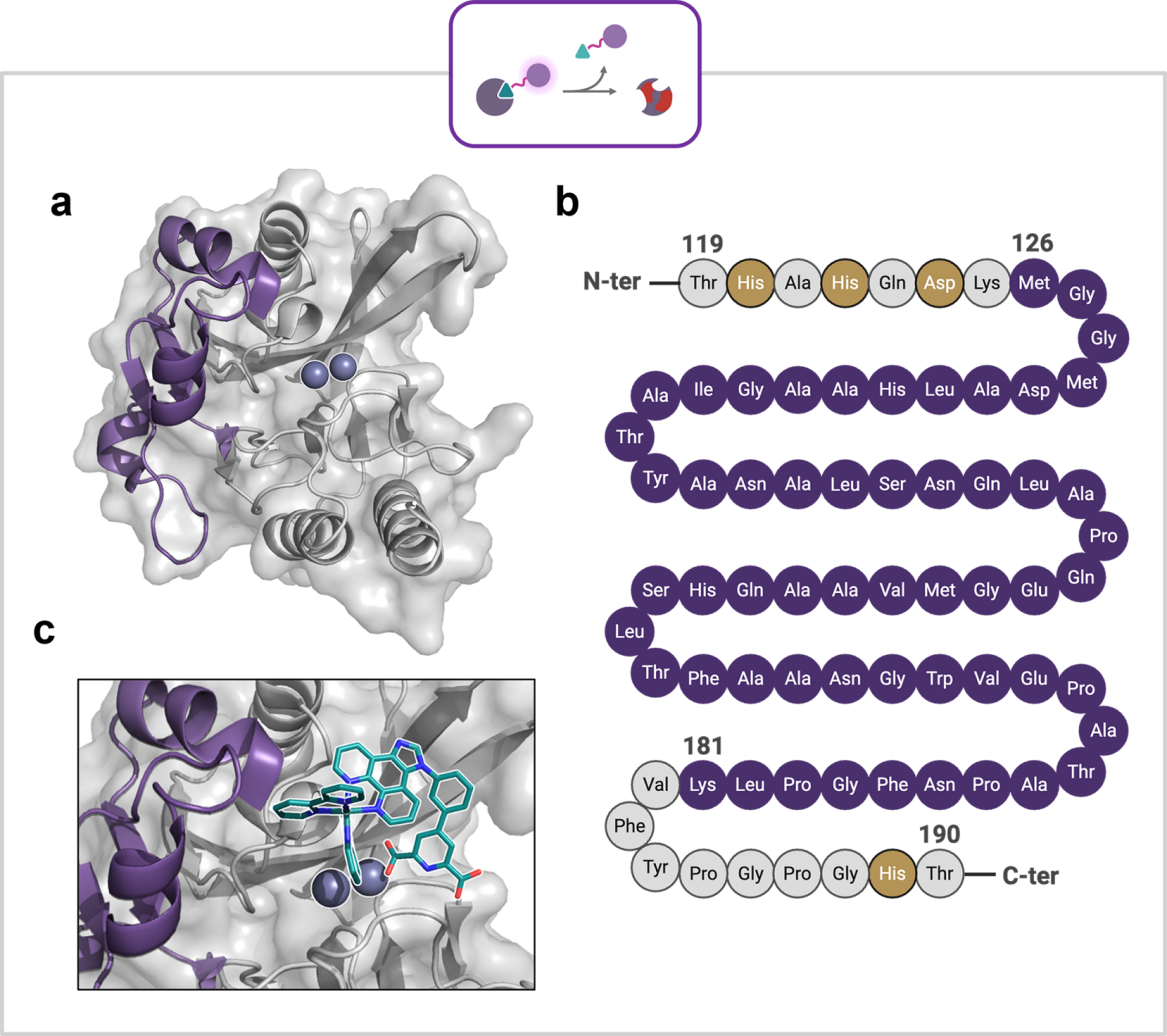
**a:** Structure of NDM-1 with the damaged region highlighted in purple. **b:** Amino acid sequence of NDM-1 from Thr119 to Thr190 with the damaged region highlighted in purple and active site residues in gold. **c:** Docking pose of **Ru1** in the active site of NDM-1. The carboxylate is coordinated to the Zn­(II) ion, while the photosensitizer is oriented toward the damaged region.

50 docking poses were generated, and the binding poses of highest performing clusters analyzed. The cluster with the best docking score revealed a binding pose that is in accordance with the report by Chen et al. showing **N1** binding to NDM-1.^61^ In this pose the majority of the complex core of the PS is adjacent to the active site and faces toward the region which is shown to be damaged by our trypsin digest LC-MS/MS study (Figure 6b and c).

### Ru1 Accumulates in *E. coli*

Due to the intrinsic and strong red phosphorescence of **Ru1** and **Ru2**, confocal microscopy was utilized to investigate the complexes’ accumulation in living bacteria. An *E. coli* MG1655 strain harboring a pSU18 vector with NDM-1 (*E. coli* NDM-1) was chosen alongside an *E. coli* MG1655 strain with the pSU18 vector but without NDM-1 as a control (*E. coli* Empty)^79–81^ *E. coli* was grown to log phase before incubation with the complexes at 100 μM for 1 h. The cells were subsequently fixed with paraformaldehyde prior to imaging. Additional control samples were prepared under the same conditions with the addition of the peptidoglycan stain 3-[[(7-hydroxy-2-oxo-2H-1-benzopyran-3-yl)­carbonyl]­amino]-d-alanine hydrochloride (HADA, 100 μM) during incubation. **Ru1** could be observed internalized in both strains (Figure 7 and Figure S36). In the samples costained with HADA the peptidoglycan is clearly stained and there is no colocalization with **Ru1**, which appears to be mainly in the cytoplasm. Under the same conditions, however, **Ru2** was undetectable (Figures 35 and S36).

**Figure 7:**
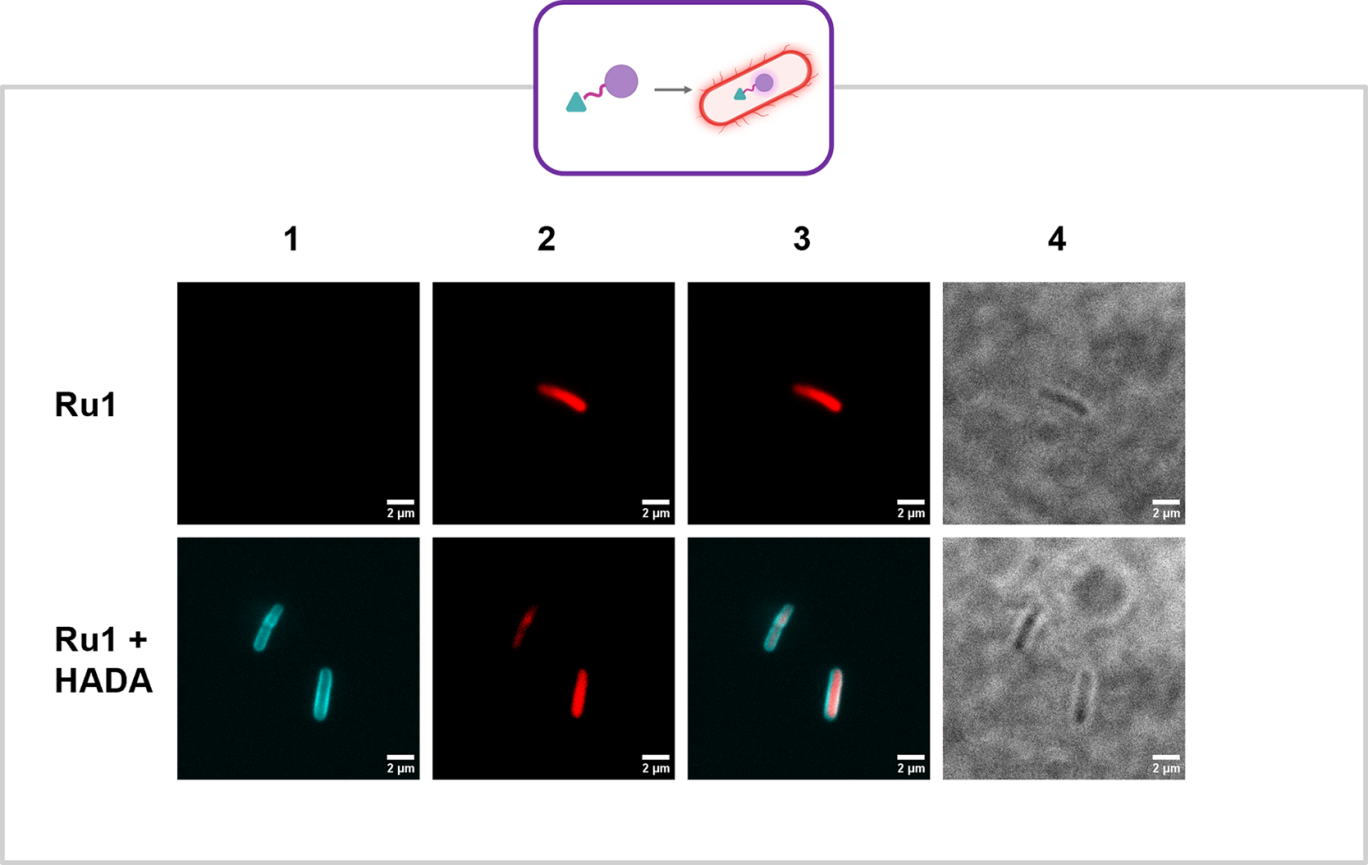
*E. coli* NDM-1 treated with **Ru1** (100 μM), with or without peptidoglycan stain HADA (100 μM). Suspended in SlowFade Gold Antifade Mountant (Thermofisher) imaged on a Visitech-international VT-iSIM confocal microscope, 150x oil objective. **1**: HADA ex/em: 405/450 nm, **2**: Ru complex ex/em: 445/680, **3**: combination of 1 and 2, **4**: brightfield. Images were analyzed using FIJI (ImageJ).

Some Ru polypyridyl complexes display either turn-on or turn-off emission depending on their cellular environment and localization. Stronger emission may not necessarily mean greater accumulation.^82^ In one study of a closely related compound by Xu et al., interaction of the complex with yeast RNA quenched emission while interaction with calf thymus DNA enhanced emission.^83^ The lack of emission of **Ru2** could therefore be due to environmental factors rather than poor cellular uptake. To confirm whether **Ru2** and **N1** accumulate within bacteria and serve as appropriate controls in bacterial assays, we quantified their intrabacterial accumulation using an LC-MS method adapted from Geddes et al.^84,85^ and Widya et al.^86^

*E. coli* NDM-1 and empty vector strains were incubated with 320 μM **Ru1**, **Ru2**, or **N1** for 1 h. In parallel, a control set was incubated without compounds for 50 min, followed by incubation with 75 μM meropenem trihydrate for 10 min. Cells were then pelleted, washed, resuspended in water, lysed, and clarified before LC-MS analysis. The results show that with a 75 μM dose, meropenem accumulates in the empty strain (150 nM/OD_600_, Figure S37) while in the NDM-1 strain it is not detectable due to the activity of NDM-1. By comparison, **Ru1** accumulates slightly more in the empty strain than the NDM-1 strain (604 vs 370 nM/OD_600_) (Figure 8). **Ru2** shows less accumulation than **Ru1** in the empty strain but a slightly higher accumulation in the NDM-1 strain (405 vs 495 nM/OD_600_). Finally, **N1** accumulates in both the empty (609 nM/OD_600_) and the NDM-1 strains (731 nM/OD_600_).

**Figure 8:**
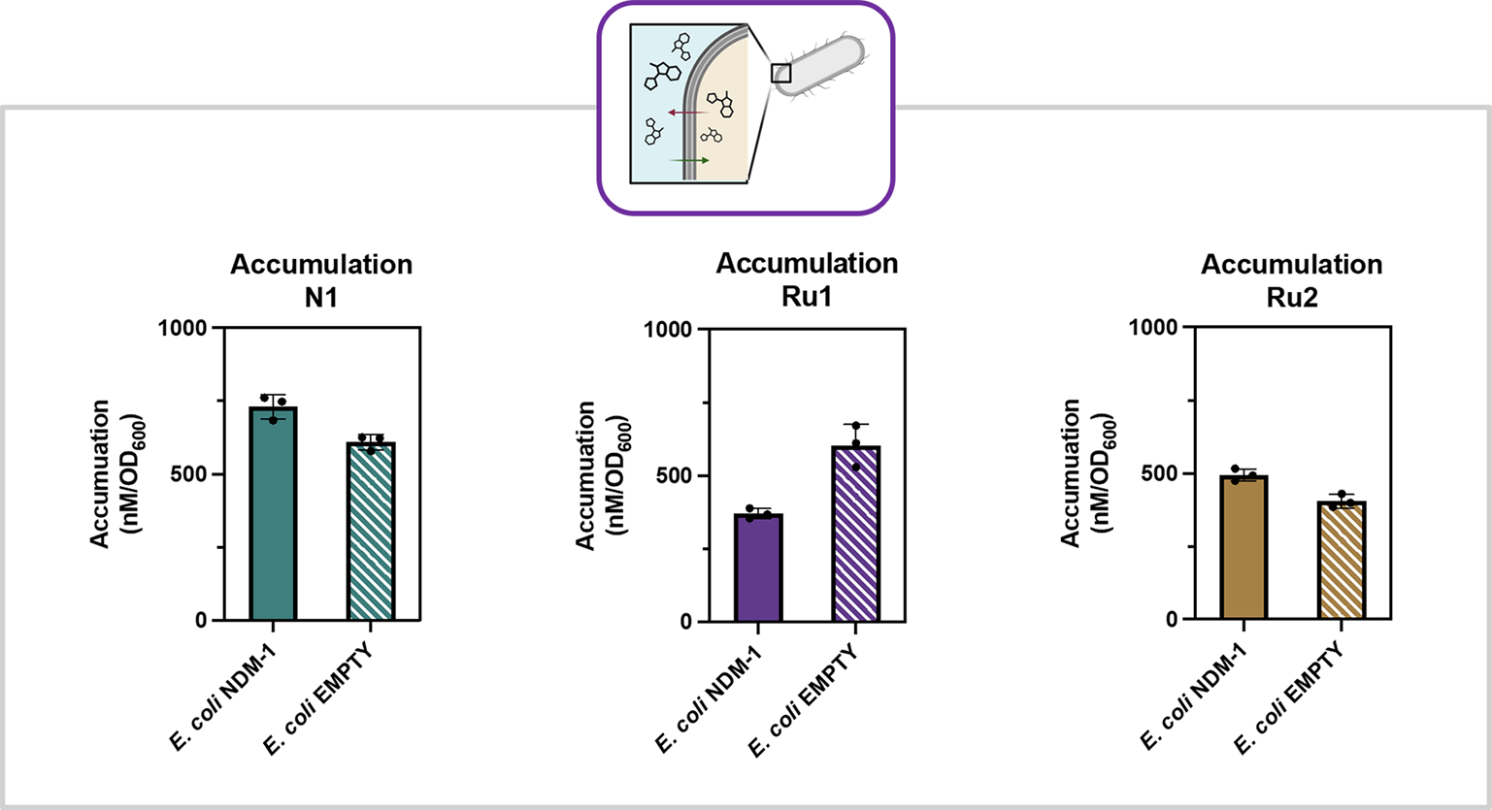
Accumulation data for compounds in *E. coli empty* and *E. coli NDM-1* strains. **N1** (Empty = 609 nM/OD_600_, NDM-1 = 731 nM/OD_600_); **Ru1** (Empty = 604 nM/OD_600_, NDM-1 = 370 nM/OD_600_); **Ru2** (Empty = 405 nM/OD_600_, NDM-1 = 494 nM/OD_600_). Error bars represent standard deviation with *n* = 3.

Given the greater lipophilicity and positive charge of **Ru2** one might expect that it would display better internalization than **Ru1** due to positive interactions with the phospholipids in the outer membrane.^87,88^ The cell wall architecture of bacteria is, however, complex and trends in accumulation are rarely straightforward. In Gram-negative bacteria, drugs must penetrate the external lipopolysaccharide layer, the outer membrane, peptidoglycan layer, and inner membrane, as well as evade defenses such as efflux pumps. While studies have sought to better understand what features make a good accumulator in Gram-negative species, this field is still a topic of major research.^85,89,90^ For example, efflux pumps are used by bacteria as defense against small molecule inhibitors. In the work by Gurvic et al., several factors (hydrogen bond donors and LogD) were shown to enhance efflux evasion.^91^ In addition, bacteria can act rapidly in response to external stimuli as new generations can emerge in a time frame of minutes. Adjusting the cell wall architecture by, for example, altering porin expression can also aid evasion of drugs.^92^ Therefore, without an in-depth study it is hard to say why this particular trend is observed for **Ru1** and **Ru2**. For the purposes of this study, we can see that **Ru1**, **Ru2**, and **N1** can accumulate in these *E. coli* strains and therefore have the potential to inhibit NDM-1 *in cellulo*.

### Ru1 Inhibits NDM-1 in Live Bacteria

Building on our finding that **Ru1** accumulates in *E. coli* and degrades and inhibits NDM-1 *in vitro*, we next aimed to show that **Ru1** can disrupt NDM-1 within live Gram-negative bacteria. The chromogenic nitrocefin assay was adapted for use with *E. coli* NDM-1 whereby the bacteria were incubated with **Ru1**, **Ru2** and **N1** under both dark and light (450 nm, 60 J cm^–2^) conditions, and the bacteria then tested for their ability to hydrolyze nitrocefin. **N1** was shown to inhibit NDM-1 with an IC_50(dark)_ of 13 ± 0.84 μM, with no change in potency upon irradiation as expected (Figure S14). **Ru1** inhibited NDM-1 with an IC_50(dark)_ of 22 ± 1.5 μM, however, upon irradiation (450 nm, 60 J cm^–2^), a 30-fold improvement in potency was observed (IC_50(light)_ = 0.75 ± 0.054 μM) (Figure 9a). To confirm that this was due specifically to targeting our PS to NDM-1, the nontargeted control complex **Ru2** was tested. **Ru2** exhibited no inhibition of NDM-1 in the dark or when irradiated with light (450 nm, 60 J cm^–2^) (Figure 9b, Figure S14), showing that this 30-fold improvement in potency can be attributed to our targeted approach.

**Figure 9:**
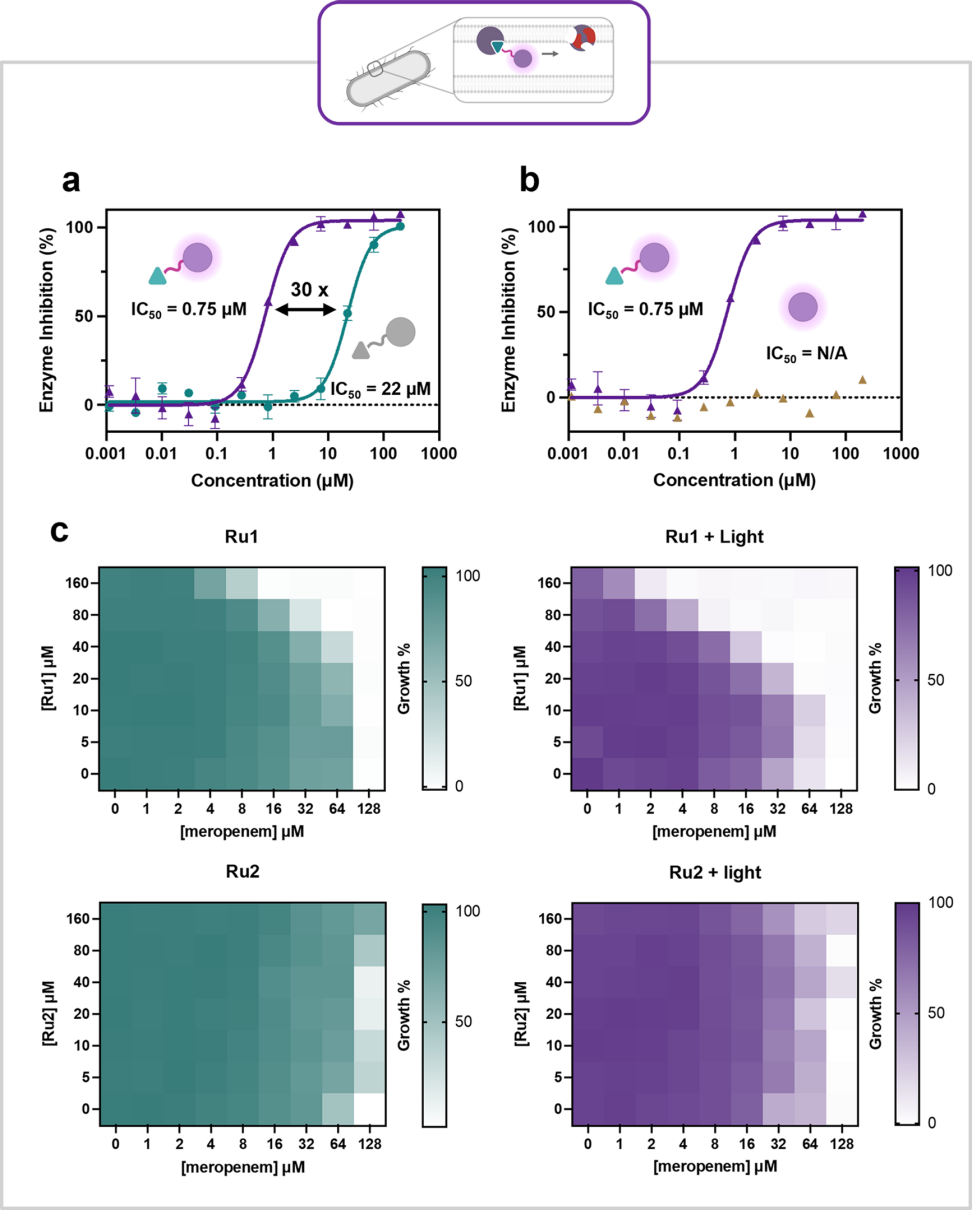
**a: Ru1** inhibits NDM-1 in *E. coli* NDM-1 in the dark (22 ± 1.5 μM), and with light irradiation (450 nm, 60 J cm^–2^), a 30-fold improvement in inhibition to 0.75 ± 0.054 μM is observed. **b:** No inhibition of NDM-1 is observed when *E. coli* NDM-1 is treated with **Ru2** under light irradiation (450 nm, 60 J cm^–2^). Error bars represent the standard deviation with *n* = 3. **c:** Heat maps representing the checkerboard assay results for **Ru1** and **Ru2** (0–160 μM) combined with meropenem (0–128 μM) in the dark and in the light (450 nm, 60 J cm^–2^). Higher intensity of color represents greater bacterial growth.

### Ru1 Rescues Meropenem Activity in Live Bacteria

Finally, to assess the ability of **N1**, and **Ru1** to rescue the efficacy of a β-lactam antibiotic in live bacteria, checkerboard minimum inhibitory concentration (MIC) assays in *E. coli* NDM-1 were performed under both dark and light (450 nm, 60 J cm^–2^) conditions alongside the antibiotic meropenem.^93^ The compounds exhibited no intrinsic inhibitory activity against either *E. coli* empty or *E. coli* NDM-1 strains up to 160 μM under both light and dark conditions (Table 2, Figures 9c and S38). This result demonstrates that PDT alone is not causing inhibition. **N1** achieved a 43-fold restoration of meropenem activity in the NDM-1 expressing strain at 160 μM (Table 3), while the nontargeted control complex, **Ru2**, showed no rescue of meropenem activity at the highest concentration tested under either condition (Figure 9c). At 160 μM, **Ru1** exhibited a 9-fold rescue of meropenem activity in the dark, which increased to a 53-fold rescue upon light irradiation (450 nm, 60 J cm^–2^) (Table 3). Across all tested concentrations (20–160 μM), **Ru1** under light irradiation outperformed all other compounds.

**Table 2:** MIC Values for **N1**, **Ru1** and **Ru2** ^a^

	MIC (μM)			
Strain	**N1**	**Ru1**	**Ru2**	**Meropenem**
*E. coli* Empty	>160	>160	>160	0.32
*E. coli* NDM-1	>160	>160	>160	128

a Both dark and light treated samples produced equivalent MIC values.

**Table 3:** Fold Change Meropenem MIC for **N1**, **Ru1** and **Ru2** in *E. coli* NDM-1^a^

	Fold change in Meropenem MIC (μM)					
	Compound concentration (μM)					
Compound	5	10	20	40	80	160
**N1 Dark**	0	0	0	0.66	2.6	35
**N1 Light**	0.66	0.66	1.3	2.0	6.6	43
**Ru1 Dark**	0	0	0.66	0.66	2.6	9.3
**Ru1 Light**	0.66	0.66	2.0	5.3	19	53
**Ru2 Dark**	0	0	0	0	0	0
**Ru2 Light**	0	0	0	0	0	0.60

a Values represent the mean of three biological replicates.

These checkerboard assay results for **Ru1** corroborate the NDM-1 inhibition observed in the chromogenic assays, both *in vitro* and in live bacteria. Finally, the safety of **N1**, **Ru1**, and **Ru2** were evaluated across a panel of seven mammalian cell lines, including HepG2, commonly used to study hepatotoxicity.^94^ Notably, none of the compounds exhibited any cytotoxicity up to 50 μM (Table S6 and Figures S39–S41).

## Conclusion

This study describes the design and synthesis of a metal-based heterobifunctional molecule **Ru1** aimed at selectively degrading NDM-1 under visible light irradiation and an in-depth characterization of its biological activity *in vitro* and in live Gram-negative bacteria. We showed that **Ru1** is a potent photosensitizer (Φ_Δ_ = 0.89) with a long-lived triplet excited state (481 ns) and effectively produces ^1^O_2_. This heterobifunctional complex binds to and inhibits NDM-1 with a >100-fold improvement on exposure to light (450 nm, 20 J cm^–2^). Comparison to the control **Ru2** shows that this targeted reactivity is driven, as designed, by the addition of a targeting vector. **Ru1** was shown to specifically bind to and degrade NDM-1 even in the presence of BSA. SDS-PAGE coupled with mass spectrometry analysis demonstrated that **Ru1** causes damage to NDM-1 at a specific location adjacent to the active site. The complex was shown to accumulate in *E. coli* via confocal microscopy and LC-MS accumulation assay. Checkerboard MICs in both an NDM-1 expressing *E. coli* strain as well as a control strain were performed. The results show that **Ru1** can inhibit NDM-1 in living bacteria and effectively rescue meropenem activity leading to *E. coli* inhibition both in the dark (9-fold improvement of meropenem MIC at 160 μM **Ru1**) and even more effectively with light activation (450 nm, 60 J cm^–2^, 53-fold improvement of meropenem MIC at 160 μM **Ru1**).

To our knowledge, this represents the first example of a metal-based bacterial protein-targeted photosensitizer. We have demonstrated that targeted degradation of a challenging antimicrobial target, namely, NDM-1, a membrane bound protein in Gram-negative bacteria, can be achieved by combining our protein-targeted photosensitizer with light irradiation. Our future goal is to expand the scope of LAMP-D to demonstrate that it is a plug and play approach which can precisely target different proteins of interest in various organisms and in a disease agnostic fashion. We anticipate that further progress in this field will drive the development of exciting chemical biology tools and therapeutics.

## Supplementary Material



## Notes

General information, materials and methods and experimental data: chemical synthesis, NMR, photophysical characterization, protein expression and purification, structural biology, enzyme inhibition and degradation assays, docking, accumulation assays, and microbiology (PDF)

## ∇

UCD School of Biomolecular and Biomedical Science, H1.38 O’Brien Centre for Science, University College Dublin, Belfield, Dublin 4, Ireland
