# Supplementary material for: Light-Activated Metal-Dependent Protein Degradation: A Heterobifunctional Ruthenium(II) Photosensitizer Targeting New Delhi Metallo-β-lactamase 1
Source: J Am Chem Soc. 2025 Nov 27;147(49):44860–74. doi: 10.1021/jacs.5c12405 (PMC12703741; doi:10.1021/jacs.5c12405)
Supplement: Supplementary file 1 [file ja5c12405_si_001.pdf]

Supporting Information

Light-Activated Metal-dependent Protein Degradation: A Heterobifunctional Ruthenium(II) Photosensitizer Targeting New Delhi Metallo-β-lactamase 1

*Lars Stevens-Cullinane,[a,b] Thomas W. Rees,[b] Calum Evans,[a,b,c] Po-Yu Ho,[a,b] Mika Kintzel,[a,b] Yew Mun Yip,[d] Ruoning Jia,[a] Jonathan Bailey,[b] Eleanor Clifford,[e] Ruqaiya Alam,[a] Sarah Maslen,[f] Stephane Mouilleron,[g] Adrien Pasquier,[h] Ok-Ryul Song,[h] Scott Warchal,[h] Joanna Redmond,[d] Michael Howell,[h] Svend Kjær,[g] Mark Skehel,[f] Manuel M. Müller,[a] Eachan O. Johnson,[c] Maxie M. Roessler,[e] and Jeannine Hess [a,b]**

a Department of Chemistry, King's College London, London, SE1 1DB, U.K.

b The Biological Inorganic Chemistry Laboratory, The Francis Crick Institute, London, NW1 1AT, U.K.

c Systems Chemical Biology of Infection and Resistance Laboratory, The Francis Crick Institute, London, NW1 1AT, U.K.

d Chemical Biology Science and Technology Platform, The Francis Crick Institute, London, NW1 1AT, U.K.

e Department of Chemistry and Centre for Pulse EPR Spectroscopy (PEPR), Imperial College London, London, W12 0BZ, U.K.

f Proteomics Science and Technology Platform, The Francis Crick Institute, London, NW1 1AT, U.K.

g Structural Biology Science and Technology Platform, The Francis Crick Institute, London, NW1 1AT, U.K.

h High Throughput Screening Science and Technology Platform, The Francis Crick Institute, London, NW1 1AT, U.K.

Corresponding author: jeannine.hess@crick.ac.uk; jeannine.hess@kcl.ac.uk

Table of Contents

1.1 Synthesis and Characterisation of Compounds 4
1.1.1 Materials and Methods 4
1.1.2 Dimethyl 4-(3-aminophenyl)pyridine-2,6-dicarboxylate dihydrochloride (1) 4
1.1.3 4-(3-aminophenyl)pyridine-2,6-dicarboxylate dihydrochloride (N1.HCl) 5
1.1.4 [Ru(2,2'-bipyridine)$_2$(1,10-phenanthroline-5,6-dione)](PF_6)$_2$ (2) 6
1.1.5 [Ru(2,2'-bipyridine)2(1-(4-(3-aminophenyl)pyridine-2,6-dicarboxylic acid) -imidazo[4,5,f][1,10]phenanthroline)](PF_6)$_2$ (Ru1) 7
1.1.6 [Ru(2,2'-bipyridine)2(1-phenyl-imidazo[4,5,f][1,10]phenanthroline)](PF6)$_2$ (Ru2) 8
1.1.7 Chromatographic LogD measurements 9

1.2 Photophysical Characterisation 10
1.2.1 UV-Vis absorption spectra of Ru1 and Ru2 10
1.2.2 Excitation and emission spectra of Ru1 and Ru2 10
1.2.3 Photostability 11
1.2.4 Electron Paramagnetic Resonance (EPR) 11
1.2.5 $^1O_2$ Quantum Yield 12
1.2.6 Luminescence lifetime 14

1.3 Protein Expression and Purification 14
1.3.1 Protein Expression and Purification 14

1.4 Structural Biology 15
1.4.1 Differential Scanning Fluorimetry (DSF) 15

1.5 Steady State Enzyme Kinetics 16

1.6 Enzyme Inhibition Assay 17
1.6.1 Inhibition of recombinant NDM-1 *in vitro* 17
1.6.2 Inhibition of NDM-1 in *E. coli* NDM-1 19

1.7 HDAC Deacetylation Assay 21
1.7.1 HDAC1 Assay conditions: 21

1.8 Protein degradation via SDS-PAGE 22
1.8.1 Ru1 and Ru2 (20 J cm^{-2}, 1 – 100 eq.) 23
1.8.2 Ru1 (60 J cm^{-2}, 1 – 100 eq.) 23
1.8.3 BSA+NDM-1 selectivity experiment 24

1.9 Liquid Chromatography-Mass Spectrometry (LC-MS) 25
1.9.1 Intact Mass Spectrometry 25
1.9.2 Trypsin Digest Mass Spectrometry 34

1.10 Molecular Docking Studies 36

1.11 Confocal Microscopy 36

1.12 LC-MS accumulation assay 38
1.12.1 Bacterial Strains and Growth Conditions: 38
1.12.2 Preparation of Accumulation Assay Samples for LC-MS Analysis: 38
1.12.3 LC-MS Analysis: 39
1.12.4 Data and statistical analysis: 40

1.13 Checkerboard Broth Microdilution Minimum Inhibitory Concentration (MIC) assay 40

1.14 Cell Viability Studies 42

1.15 NMR spectra **46**

1.16 References **52**

1.1 Synthesis and Characterisation of Compounds

1.1.1 Materials and Methods

Reagents were purchased from Sigma-Aldrich (U.K.), Fluorochem (U.K.) and ThermoFischer (U.K.) and were used without further purification. Column chromatography was carried out using a Biotage Selekt with Biotage Sfär Duo silica columns. ^{1}H NMR, ^{13}C NMR and ^{19}F NMR spectra were recorded in D_2O, MeOH-d_4 or CD_3CN-d_3 on either a Bruker Ascend 400 or a Bruker Ascend 600 instrument and processed using MestReNova. The following abbreviations were used to explain NMR peak multiplicities: s = singlet, d = doublet, t = triplet, q = quartet, m = multiplet, stack = two or more indistinguishable overlaid environments, dd = doublet of doublets, ddd = doublet of doublet of doublets, dt = doublet of triplets, ddt = doublet of doublet of triplets. High resolution mass spectral data was acquired using a Waters Xevo G2-XS QTof instrument equipped with an Acquity UPLC BEH C18 1.7 µm 2.1 x 50 mm column using a gradient of H_2O/CH_3CN (+ 0.1% formic acid) 95–5% over 5 min. LC-MS of small molecules was measured using a Waters Acquity UPLC equipped with an Acquity UPLC BEH C18 1.7 µm 2.1 x 50 mm column using a gradient of H_2O/CH_3CN (+ 0.1% formic acid) 95–5% over 4 min.

1.1.2 Dimethyl 4-(3-aminophenyl)pyridine-2,6-dicarboxylate dihydrochloride (1)

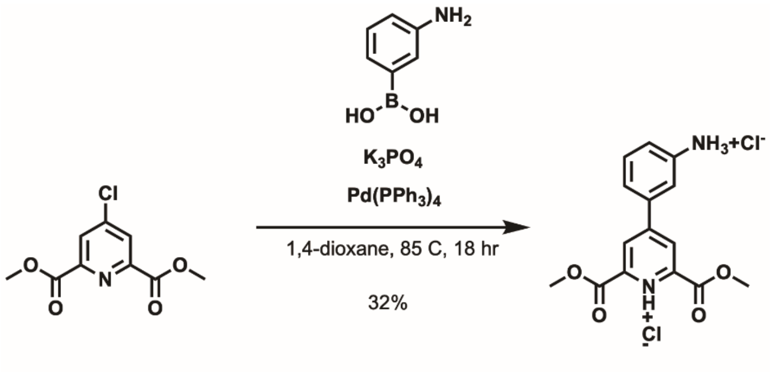

The synthesis of **1** was modified from a previously reported procedure.[1] Dimethyl 4-chloropyridine-2,6-dicarboxylate (1.95 g, 8.49 mmol, 1.00 equiv), 3-aminophenyl-boronic acid (1.86 g, 13.6 mmol, 1.60 equiv) and K_3PO_4 (5.41 g, 25.5 mmol, 3.00 equiv) were dissolved in anhydrous 1,4-dioxane (30 mL) and degassed using argon. To this suspension, tetrakis(triphenylphosphine)palladium(0) (1.47 g, 1.27 µmol, 0.150 equiv) was added and the mixture heated to 85 °C and stirred overnight. The reaction mixture was then filtered through Celite and the solvent removed by evaporation under reduced pressure. The crude mixture was dissolved in CH_2Cl_2 and washed with H_2O, brine, and dried over $MgSO_4$. The solvent was removed under reduced pressure to provide the crude material as a yellow oil.

The crude product was purified by normal phase column chromatography, eluting with ethyl acetate in cyclohexane (20–60%), to provide the desired compound. This material was dissolved in a small volume of 1,4-dioxane and treated with 4 M HCl in 1,4-dioxane. The

precipitate was filtered, washed with 1,4-dioxane and dried *in vacuo* to provide the desired compound **1** as a light-yellow solid (997 mg, 2.78 mmol, 32%).

^{1}H NMR (400 MHz, D_2O) δ 8.29 (s, 2H), 7.71 (dt, *J* = 8.0, 1.6 Hz, 1H), 7.67 – 7.60 (m, 2H), 7.51 (ddd, *J* = 7.9, 2.2, 1.1 Hz, 1H), 3.95 (s, 6H).

^{13}C NMR (101 MHz, D_2O) δ 165.4, 149.4, 147.7, 136.8, 132.4, 131.1, 126.9, 125.6, 124.3, 120.8, 53.4.

LC-MS: RT = 1.96 min, (ESI+): *m/z* $[M+H]^+$ = 287.2

ESI-TOF-HRMS: *m/z* calcd for $[M+H]^+$ $C_{15}H_{15}N_2O_4$, 287.1032; found 287.1019

1.1.3 4-(3-aminophenyl)pyridine-2,6-dicarboxylate dihydrochloride (N1.HCl)

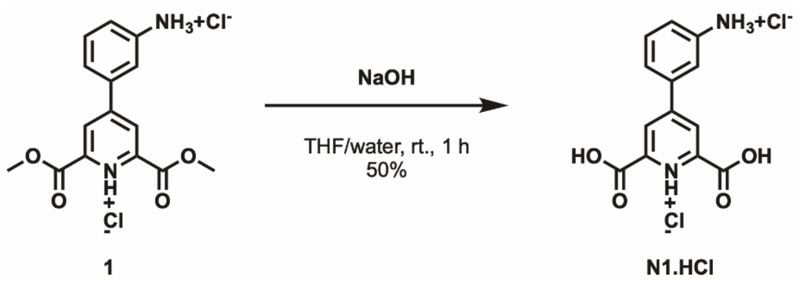

1 (200 mg, 557 µmol, 1.00 equiv) was suspended in THF (3 mL), treated with 1 M NaOH (2.78 mL, 2.78 mmol, 5.00 equiv) and stirred at room temperature for 1 h. The suspension was filtered and the solids washed with THF (2 mL). The solids were then dissolved in H_2O and washed off the filter cake. To the filtrate was added 2 M HCl drop-wise to form a precipitate. This suspension was filtered and the solids washed with pH ~4 H_2O (acidified with HCl), diethyl ether, and dried *in vacuo* to provide **N1.HCl** as a grey solid (92.0 mg, 557 µmol, 50%).

^{1}H NMR (400 MHz, DMSO) δ 8.37 (s, 2H), 7.20 (t, *J* = 7.8 Hz, 1H), 7.09 (t, *J* = 2.0 Hz, 1H), 7.01 (ddd, *J* = 7.7, 1.9, 1.0 Hz, 1H), 6.72 (ddd, *J* = 8.0, 2.2, 0.9 Hz, 1H).

^{13}C NMR (101 MHz, DMSO) δ 165.6, 150.6, 149.7, 149.0, 136.2, 130.1, 124.0, 115.6, 114.1, 111.8.

LC-MS: RT = 1.26 min, (ESI+): *m/z* $[M-2Cl+H]^+$ = 259.1

ESI-TOF-HRMS: *m/z* calcd for $[M+H]^+$ $C_{13}H_{11}N_2O_4$, 259.0719; found 259.0702

1.1.4 [Ru(2,2'-bipyridine)$_2$(1,10-phenanthroline-5,6-dione)](PF$_6$)$_2$ (2)

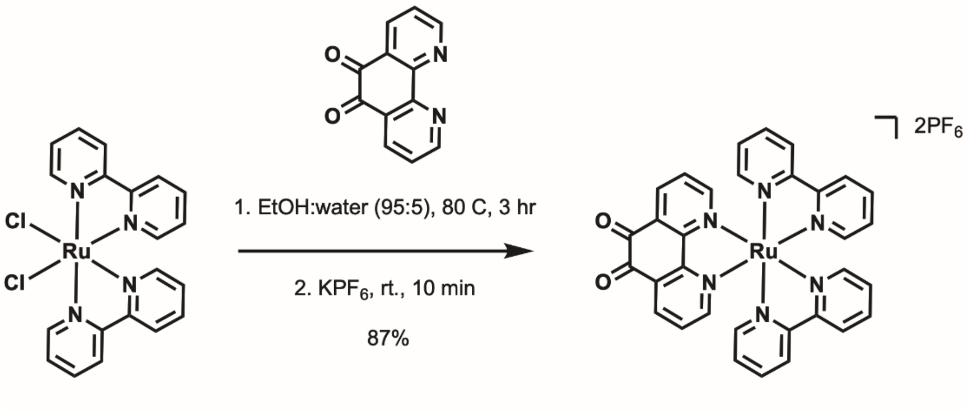

2

The synthesis of **2** was modified from a previously reported procedure.[2] Ru(bpy)$_2$Cl$_2$ (359 mg, 714 µmol, 1.00 equiv) and 1,10-phenanthroline-5,6-dione (150 mg, 714 µmol, 1.00 equiv) were suspended in 50 mL of ethanol: H_2O (95:5) and degassed using argon. The solution was refluxed for 3 h in the dark. The reaction mixture was cooled to room temperature and KPF$_6$ (1.20 g) in H_2O (10 mL) was added, which was then concentrated until a precipitate formed. The sample was cooled at 0 °C for 1 h. The precipitate was then collected by filtration, washed with H_2O, dried and then washed with diethyl ether. The solids were filtered, collected and dried *in vacuo* to afford the desired compound **2** as a brown/black solid (570 mg, 624 µmol, 87%).

^{1}H NMR (400 MHz, CD$_3$CN) δ 8.52 (stack, 6H), 8.09 (tt, *J* = 7.9, 1.7 Hz, 4H), 7.98 (d, *J* = 5.6 Hz, 2H), 7.85 – 7.80 (m, 2H), 7.77 – 7.73 (m, 2H), 7.61 (dd, *J* = 7.9, 5.6 Hz, 2H), 7.43 (ddt, *J* = 7.4, 5.8, 1.6 Hz, 4H).

^{13}C NMR (101 MHz, CD$_3$CN) δ 176.3, 158.0, 157.9, 157.5, 157.3, 153.2, 152.9, 139.3, 139.2, 136.9, 129.8, 128.7, 128.6, 125.5, 125.5.

^{19}F NMR (376 MHz, CD$_3$CN) δ -72.84 (d, *J* = 706.5 Hz).

LC-MS: RT = 1.55 min, (ESI+): *m/z* [M-2PF$_6$]$^{2+}$ = 312.1

ESI-TOF-HRMS: *m/z* calcd for [M-2PF$_6$]$^{2+}$ $C_{32}H_{22}F_{12}N_6O_2P_2Ru$, 312.0424; found 312.0430

1.1.5 [Ru(2,2'-bipyridine)2(1-(4-(3-aminophenyl)pyridine-2,6-dicarboxylic acid) -imidazo[4,5,f][1,10]phenanthroline)]$(PF_6)_2$ (Ru1)

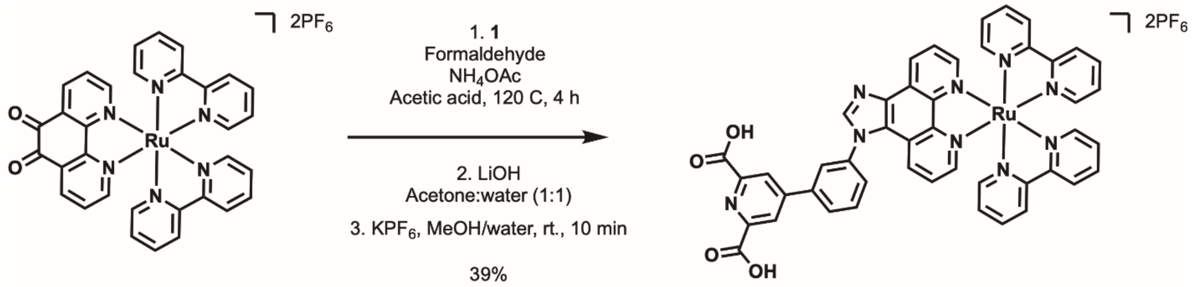

2 **Ru1**

2 (200 mg, 219 µmol, 1.00 equiv) and **1** (79.0 mg, 219 µmol, 1.00 equiv) were dissolved in glacial acetic acid (10 mL) and degassed using argon. To this solution was added formaldehyde (36% solution in H_2O, 19.0 µL, 241 µmol, 1.10 equiv) and ammonium acetate (337mg, 4.38 mmol, 20.0 equiv), and the mixture heated to 120 °C for 4 h in the dark. The reaction mixture was cooled to room temperature and the solvent removed under reduced pressure. The crude oily solids were then dissolved in acetone:H_2O (10 mL, 1:1). $LiOH.H_2O$ was added until the pH was adjusted to 12-14, and the mixture stirred at room temperature for 90 min. The reaction mixture was concentrated under reduced pressure and partitioned between CH_2Cl_2 and H_2O. The aqueous layer was washed with CH_2Cl_2 (3 x), acidified with 1 M HCl and the solvent removed under reduced pressure. The solids were dissolved in a minimal amount of MeOH and solid KPF_6 (403 mg, 2.19 mmol, 10.0 equiv) was added. The drop-wise addition of H_2O then initiated precipitation of an orange solid. The suspension was cooled on ice and the orange solids were filtered and washed with pH ~4 H_2O (acidified with HCl) and diethyl ether. The filter cake was washed through with CH_3CN and the solvent removed under reduced pressure to afford the crude material as an orange solid.

The crude material was purified by reverse phase, eluting with CH_3CN in H_2O (+ 0.1% formic acid) (0 – 30%) to provide the mixed-counterion product as an orange solid. The solids were dissolved in MeOH and solid KPF_6 (100 mg) was added. The drop-wise addition of H_2O then initiated precipitation of an orange solid. The orange solids were filtered, washed with pH ~4 H_2O (acidified with HCl), and diethyl ether. The filter cake was washed through with CH_3CN and the solvent removed under reduced pressure to afford the purified **Ru1** as an orange solid (100 mg, 85.8 µmol, 39%).

^{1}H NMR (600 MHz, MeOD) δ 9.21 (dd, *J* = 8.3, 1.3 Hz, 1H), 8.78 (s, 1H), 8.71 (stack, 4H), 8.61 (s, 1H), 8.57 (s, 1H), 8.27 (stack, 2H), 8.20 – 8.18 (m, 1H), 8.18 – 8.11 (m, 2H), 8.09 – 8.01 (m, 4H), 8.00 – 7.89 (m, 5H), 7.68 (d, *J* = 5.5 Hz, 2H), 7.58 (dd, *J* = 8.6, 5.3 Hz, 1H), 7.56 – 7.50 (m, 2H), 7.33 (t, *J* = 6.7 Hz, 2H).

^{13}C NMR (151 MHz, MeOD) δ 168.8, 158.7, 158.7, 158.5, 152.8, 152.8, 151.9, 151.5, 150.9, 147.7, 147.5, 147.3, 141.0, 139.3, 139.2, 132.9, 132.2, 130.7, 130.5, 129.0, 128.9, 127.8, 127.6, 127.3, 126.8, 125.6, 125.6, 125.4, 123.4.

^{19}F NMR (564 MHz, MeOD) δ -74.70 (d, *J* = 707.9 Hz).

LC-MS: RT = 1.62 min, (ESI+): *m/z* $[M-2PF_6]^{2+}$ = 437.6; RT = 1.77 min, (ESI+): *m/z* $[M-2PF_6-2H+2Na]^{2+}$ = 459.4

ESI-TOF-HRMS: *m/z* calcd for $[M-2PF_6]^{2+}$ $C_{46}H_{31}F_{12}N_9O_4P_2Ru$, 437.5771; found 437.5793

1.1.6 [Ru(2,2'-bipyridine)2(1-phenyl-imidazo[4,5,f][1,10]phenanthroline)]$(PF6)_2$ (Ru2)

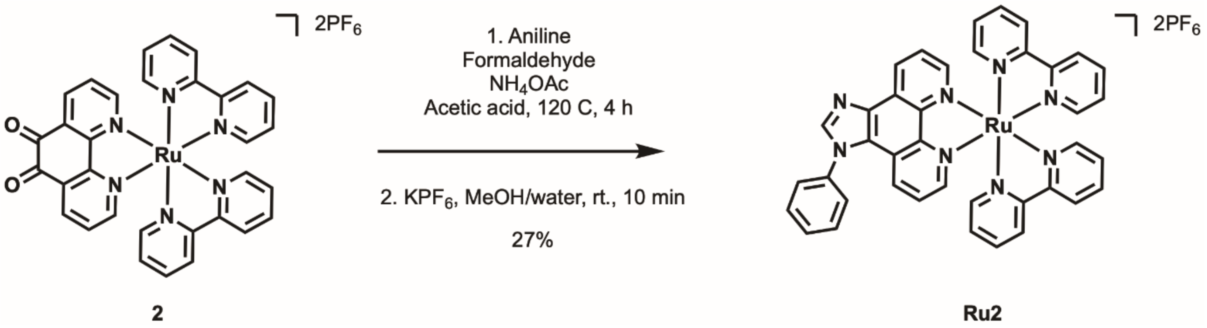

2 (200 mg, 219 µmol, 1.00 equiv) and aniline (23.9 µL, 219 µmol, 1.00 equiv) were dissolved in glacial acetic acid (10 mL) and degassed using argon. To this solution was added formaldehyde (36% solution in H_2O, 19.0 µL, 241 µmol, 1.10 equiv) and ammonium acetate (337mg, 4.38 mmol, 20.0 equiv), and the mixture heated to 120 °C for 4 h in the dark. The reaction mixture was cooled to room temperature and the solvent removed under reduced pressure to afford the crude material as an orange oily solid.

The crude material was purified by reverse phase, eluting with CH_3CN in H_2O (0 – 30%) to provide the mixed-counterion product as an orange solid. The solids were dissolved in MeOH and solid KPF_6 (100 mg) was added. The drop-wise addition of H_2O then initiated precipitation of an orange solid. The orange solids were filtered, washed with H_2O, and diethyl ether. The filter cake was washed through with CH_3CN and the solvent removed under reduced pressure to afford the purified **Ru2** as an orange solid (60.0 mg, 60.0 µmol 27%).

^{1}H NMR (400 MHz, CD_3CN) δ 9.13 (dd, *J* = 8.3, 1.3 Hz, 1H), 8.51 (dt, *J* = 13.6, 8.7 Hz, 4H), 8.41 (s, 1H), 8.09 (stack, 3H), 8.00 (ddt, J = 8.0, 4.1, 2.1 Hz, 2H), 7.96 (dd, *J* = 5.3, 1.2 Hz, 1H), 7.83 (stack, 4H), 7.74 (stack, 5H), 7.59 (d, *J* = 5.6 Hz, 1H), 7.54 (d, *J* = 5.6 Hz, 1H), 7.44 (stack, 3H), 7.25 – 7.19 (m, 2H).

^{13}C NMR (101 MHz, CD_3CN) δ 158.2, 158.1, 158.0, 152.9, 152.9, 152.8, 151.7, 151.3, 147.2, 147.0, 146.6, 138.9, 138.9, 138.8, 138.7, 138.5, 137.2, 131.8, 131.6, 131.6, 130.1, 128.6, 128.4, 128.4, 128.1, 127.5, 127.4, 127.3, 126.3, 125.3, 125.2, 123.1.

^{19}F NMR (376 MHz, CD_3CN) δ -72.93 (d, *J* = 706.4 Hz)

LC-MS: RT = 1.66 min, (ESI+): *m/z* = 355.2 $[M-2PF_6]^{2+}$

ESI-TOF-HRMS: *m/z* calcd for $[M-2PF_6]^{2+}$ $C_{39}H_{28}F_{12}N_8P_2Ru$, 355.0740; found 355.0740

1.1.7 Chromatographic LogD measurements

ChromLogD measurements were adapted from a previously reported procedure.[3] The reversed phase HPLC measurements were measured using an Agilent 1100 Series, equipped with an Eclipse Plus C18 column 4.6 x 50 mm, using a gradient of 50 mM ammonium acetate/CH_3CN 0 – 100% over 10 min. The mobile phase was adjusted to pH = 7.4 using concentrated sodium hydroxide.

The retention times (log *k'*) of seven commercially available compounds which cover the log D range from -0.02 to 3.18 (theophylline, 5-phenyl-1H-tetrazole, benzimidazole, colchicine, acetophenone, indole, propiophenone, butyrophenone, and benzophenone) were calculated using the below equation, where t_R is the retention time of the compound and t_0 is the column dead time, measured by injecting uracil (0.853 min).

$$Log\ k' = Log(\frac{t_R - t_0}{t_o})$$

The average retention times (t_R) of two consecutive injections of sample were used to calculate the log k' values, which were then used to create the standard curve (**Figure S1**). ChromLog D values for **N1**, **Ru1** and **Ru2** were then calculated using the standard curve.

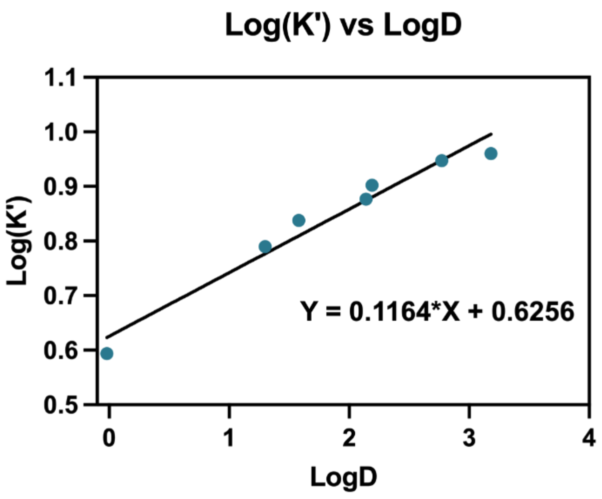

Figure S1:Standard curve of seven representative compounds with known log P values: theophylline, 5-phenyl-1H-tetrazole, benzimidazole, colchicine, acetophenone, indole, propiophenone, butyrophenone, and benzophenone. Where log K' = isocratic retention time and log D = distribution coefficient.

*Table S1: ChromLogD values for **N1**, **Ru1**, and **Ru2***

Compound	ChromLogD
**N1**	-1.32
**Ru1**	-0.04
**Ru2**	1.21

1.2 Photophysical Characterisation

UV−Vis spectra were recorded on an Edinburgh Instruments DS5 UV-Vis spectrophotometer in PBS buffer or methanol. Irradiation of samples was performed using a Height-LED SZUV-II control unit equipped with a 450 nm LED array.

1.2.1 UV-Vis absorption spectra of Ru1 and Ru2

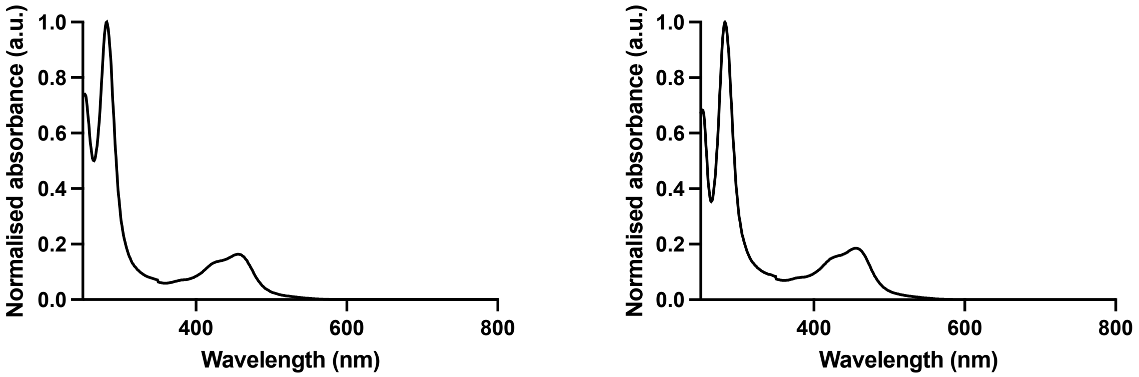

Figure S2: Normalised UV-Vis absorption spectra of ***Ru1*** *(left) and* ***Ru2*** *(right) in PBS.*

1.2.2 Excitation and emission spectra of Ru1 and Ru2

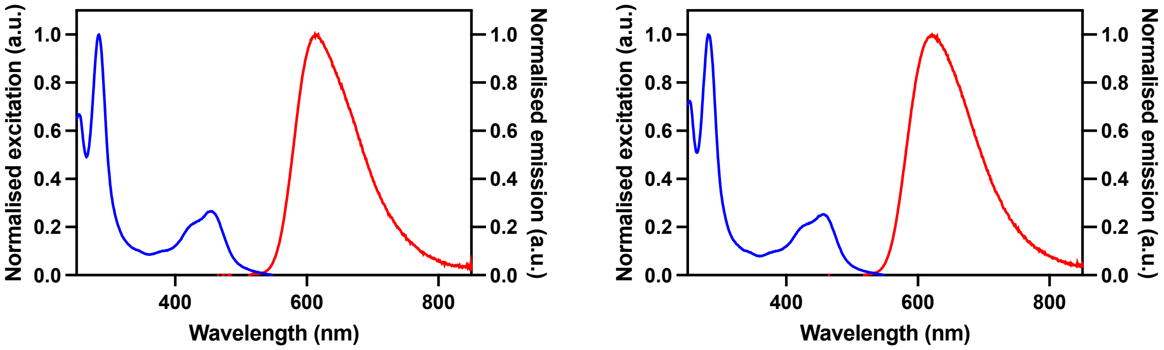

Figure S3: Normalised excitation (blue) and emission (red) spectra of ***Ru1*** *in MeOH (left) and PBS (right)*

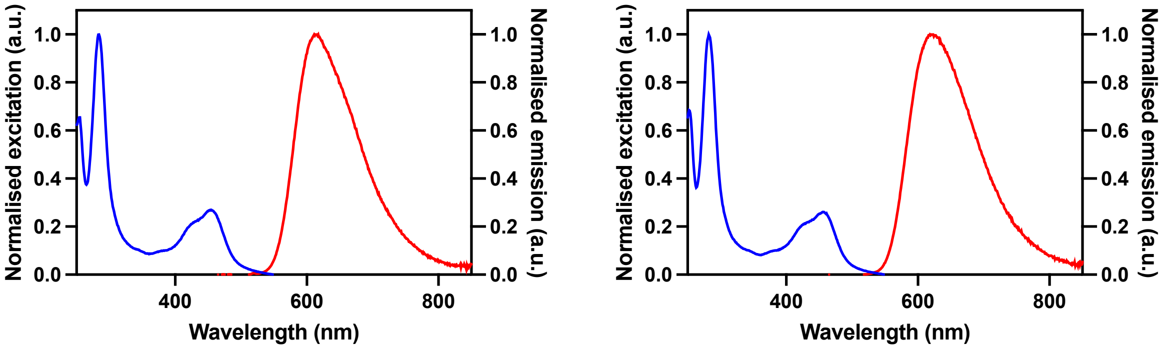

Figure S4: Normalised excitation (blue) and emission (red) spectra of ***Ru2*** *in MeOH (left) and PBS (right).*

1.2.3 Photostability

The photostability of **Ru1** and **Ru2** was performed by measuring the changes in the absorption spectra of both compounds in PBS (10 µM) under light irradiation (450 nm, 23 J cm^{-2} over 240 s at 40 s intervals).

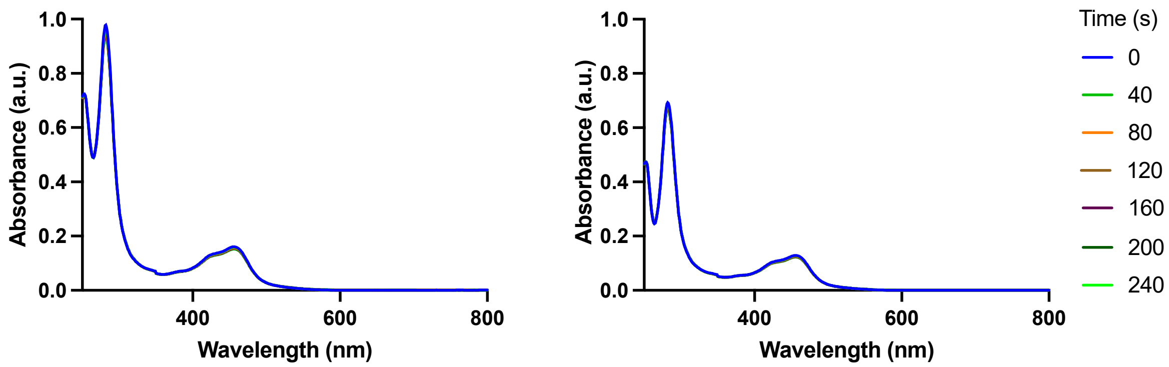

*Figure S5: Photostability of **Ru1** (left) and **Ru2** (right) in PBS.*

1.2.4 Electron Paramagnetic Resonance (EPR)

All EPR measurements were performed using a Bruker Magnettech spectrometer (ESR5000), Bruker (Germany), at room temperature. EPR samples were transferred to a 20 µL BLAUBRAND micropipette and spectra were recorded at 9.47 GHz microwave frequency, 2 mW microwave power, 100 kHz modulation frequency and 0.1 mT modulation amplitude. The field was scanned from 331.3 mT to 343.3 mT and a single scan was acquired.

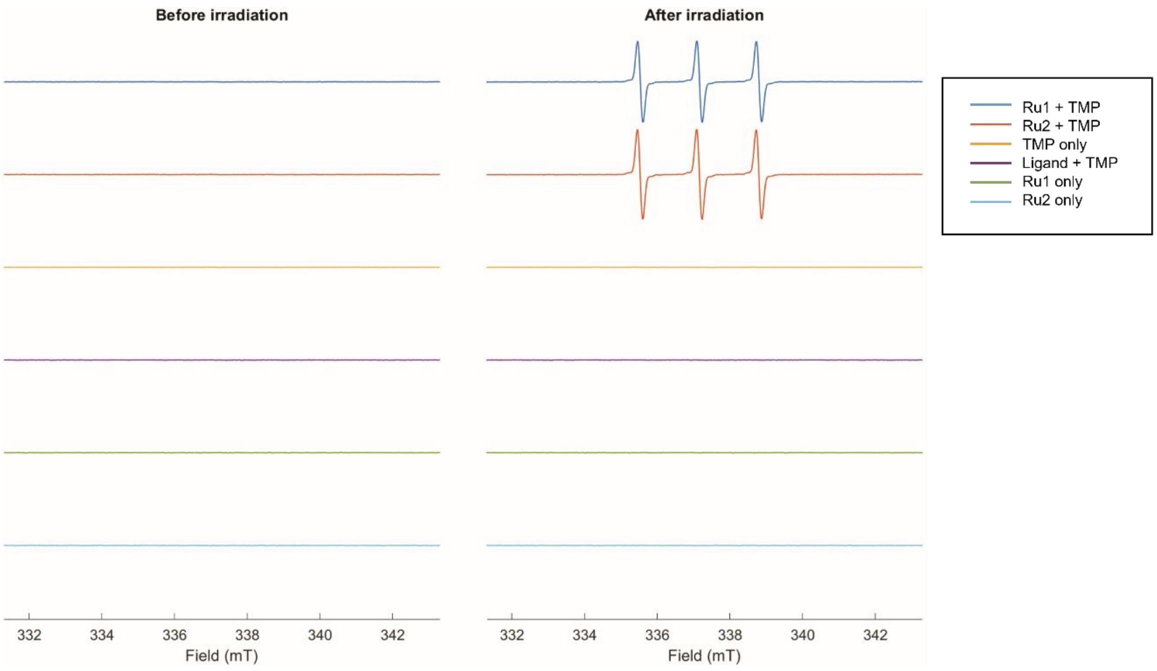

*Figure S6: EPR spectra before and after irradiation (450 nm, 20 J cm^{-2}) in methanol of **Ru1** (1 mM) + 2,2,6,6-tetramethylpiperidine (TMP) (20 mM), **Ru2** (1 mM) + TMP (20 mM), TMP (20 mM), **N1** (1 mM) + TMP (20 mM), **Ru1** (1*

*mM) and **Ru2** (1 mM). Only when **Ru1** and/or **Ru2** are irradiated in the presence of TMP is a distinctive triplet signal observed, indicative of $^1O_2$ production.*

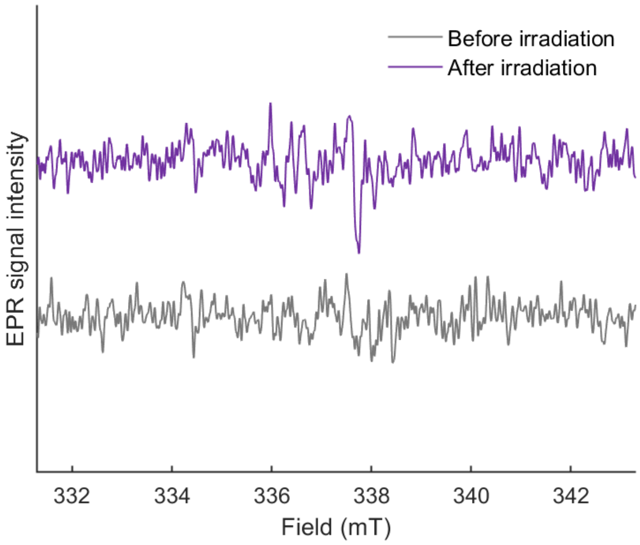

*Figure S7: EPR spectrum of **Ru1** (1 mM) and 5,5-dimethyl-1-pyrroline N-oxide (DMPO) (20 mM) in methanol before and after irradiation (450 nm, 20 J cm^{-2}). No signal is observed before or after irradiation indicating that no ·OH is produced by **Ru1**.*

1.2.5 $^1O_2$ Quantum Yield

The $^1O_2$ quantum yields were determined by tracking the photooxidation of 1,3-diphenylisobenzofuran (DPBF) (30 µM) in methanol in the presence of compounds **Ru1** and **Ru2**. The absorption at 450 nm for solutions of **Ru1, Ru2** and tris(2,2'-bipyridyl)ruthenium(II) dichloride ([Ru(bpy)$_3$]Cl$_2$) were adjusted to approximately 0.065. Methanol solutions containing **Ru1**, **Ru2**, ([Ru(bpy)$_3$]Cl$_2$) or methanol alone (control), along with DPBF, were fully aerated and exposed to LED irradiation at 450 nm (13 mW cm^{-2}). The absorbance of the reaction mixture at 411 nm was recorded at one-second intervals. ([Ru(bpy)$_3$]Cl$_2$) served as the reference for $^1O_2$ sensitization ($\Phi_\Delta = 0.87$).[4] The quantum yield of **Ru1** and **Ru2** was calculated using the below equation in which 's' represents the calibrated slope of a linear fit to the cumulative absorbance changes at 411 nm versus cumulative irradiation time. F denotes the absorption correction factor, with superscripts "s" and "r" referring to the sample and the reference ([Ru(bpy)$_3$]Cl$_2$) respectively.

$$\phi_\Delta^s = \frac{\phi_\Delta^r \times (s^s \times F^r)}{(s^r \times F^s)}$$

$$\mathrm{F} = 1 - 10^{-Abs_{450nm}}$$

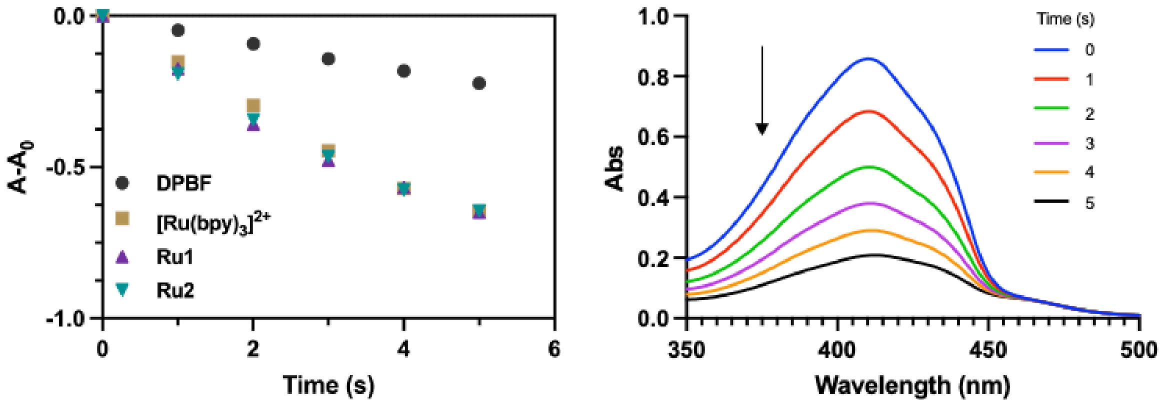

*Figure S8: Left: Measurement of $^{1}O_2$ production efficiency via changes in the absorbance of DPBF at 411 nm versus irradiation time (λ_{irr} = 450 nm) in the presence of **Ru1** and **Ru2** in aerated methanol versus $[Ru(bpy)_3]^{2+}$ as reference. Right: Overlaid UV-Vis spectra showing changes in the absorbance of DPBF in aerated methanol in the presence of **Ru1** with irradiation.*

*Table S2: Singlet oxygen quantum yield (ϕ) of compounds **Ru1**, **Ru2,** and $[Ru(bpy)_3]^{2+}$ as reference.*[4]

	**Singlet oxygen quantum yield (Φ_Δ)**
**$[Ru(bpy)_3]^{2+}$**	0.87
**Ru1**	0.89
**Ru2**	0.87

1.2.6 Luminescence lifetime

The luminescence lifetime study was conducted on solutions of **Ru1** and **Ru2** in PBS (1 µM) at room temperature with a Horiba Scientific FL-1065 (FluoroLog) using a pulse laser (486 nm) as the excitation source.

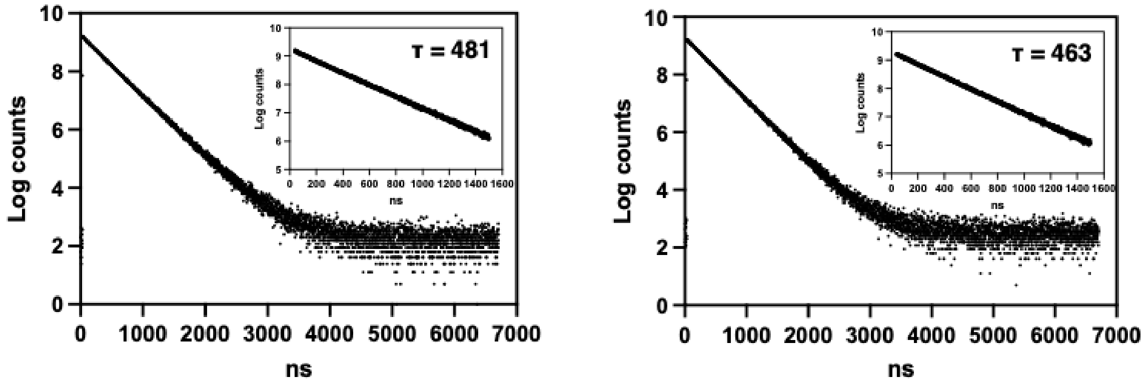

*Figure S9: Raw data of the luminescence lifetime of excited state for **Ru1** (left) and **Ru2** (right).*

*Table S3: Luminescence lifetime values of excited state for compounds **Ru1** and **Ru2**.*

	Lifetime of excited state (ns)
**Ru1**	481
**Ru2**	463

1.3 Protein Expression and Purification

1.3.1 Protein Expression and Purification

His-tagged NDM-1 (31-270) were produced in BL21 (DE3) *Escherichia coli* cells. Overnight pre-cultures were grown in LB medium, 1 mL was used to inoculate 1 L of ZYM5052 auto induced media in 2 L baffled flasks. The flasks were incubated for 11 h at 37 °C, then the temperature was lowered to 20 °C for 24 h. Harvested cells were resuspended in lysis buffer (50 mM Tris-HCl, pH 8, 10 mM imidazole, 500 mM NaCl, 1mM $ZnCl_2$), lysed by French press, clarified, and stored at -80 °C.

Clarified lysates were loaded onto a 5 mL HisTrap crude column on an AktaPure HPLC, washed with 20 CV of buffer A (50 mM Tris-HCl pH 7.5, 500 mM NaCl, 10 mM imidazole pH 8, 10 µM $ZnCl_2$, 0.5 mM TCEP), His-tagged fusion proteins were eluted in buffer B (Buffer A + 240 mM Imidazole) and purified using size exclusion chromatography on a Superdex 200 26/60 column in SEC buffer1 (50 mM Tris-HCl pH 7.5, 300 mM NaCl, 10 mM imidazole, 10 µM $ZnCl_2$, 0.5 mM TCEP). The His tag was then cleaved off by incubating overnight with His-3C protease at 4 °C and the cleaved product recovered by passage of the sample over a 5 mL HisTrap crude column. Protein was then concentrated and further purified on a Superdex 75

equilibrated in SEC buffer2 (20 mM Tris-HCl pH 7.5, 200 mM NaCl, 100 µM $ZnCl_2$, 0.5 mM TCEP). The protein was then concentrated to 15 mg/mL and stored at -80 °C.

1.4 Structural Biology

1.4.1 Differential Scanning Fluorimetry (DSF)

DSF was performed using the Applied Biosystems QuantStudio 5 thermal cycler (Thermo Fisher Scientific). Results were analysed and T_m determined with the Applied Biosystems Protein Thermal Shift Software v1.4 and T_m values determined by the software. DSF was conducted in sealed MicroAmp Optical 384-Well Reaction Plates (Thermo Fisher Scientific). The DSF run included a 10 min pre-incubation at 25 °C followed by heating from 25 – 98 °C at a temperature ramp rate of 0.03 °C/s. For compound-protein interaction studies, a final concentration of 3.125 µM NDM-1 and a range of 1000 – 2 µM compound was used in a buffer containing 5X SYPRO Orange (Thermo Fisher Scientific), 50 mM HEPES and 5%(v/v) DMSO-d_6. The final reaction volume was 20 µL per well. All DMSO compound stocks used for DSF experiments were freshly prepared from solid stocks. All DSF measurements were carried out in triplicate and the T_m reported is the mean value and the error is the standard deviation. For all compounds tested, a no protein control run was included to see potential changes in fluorescence resulting from a compound-dye interaction.

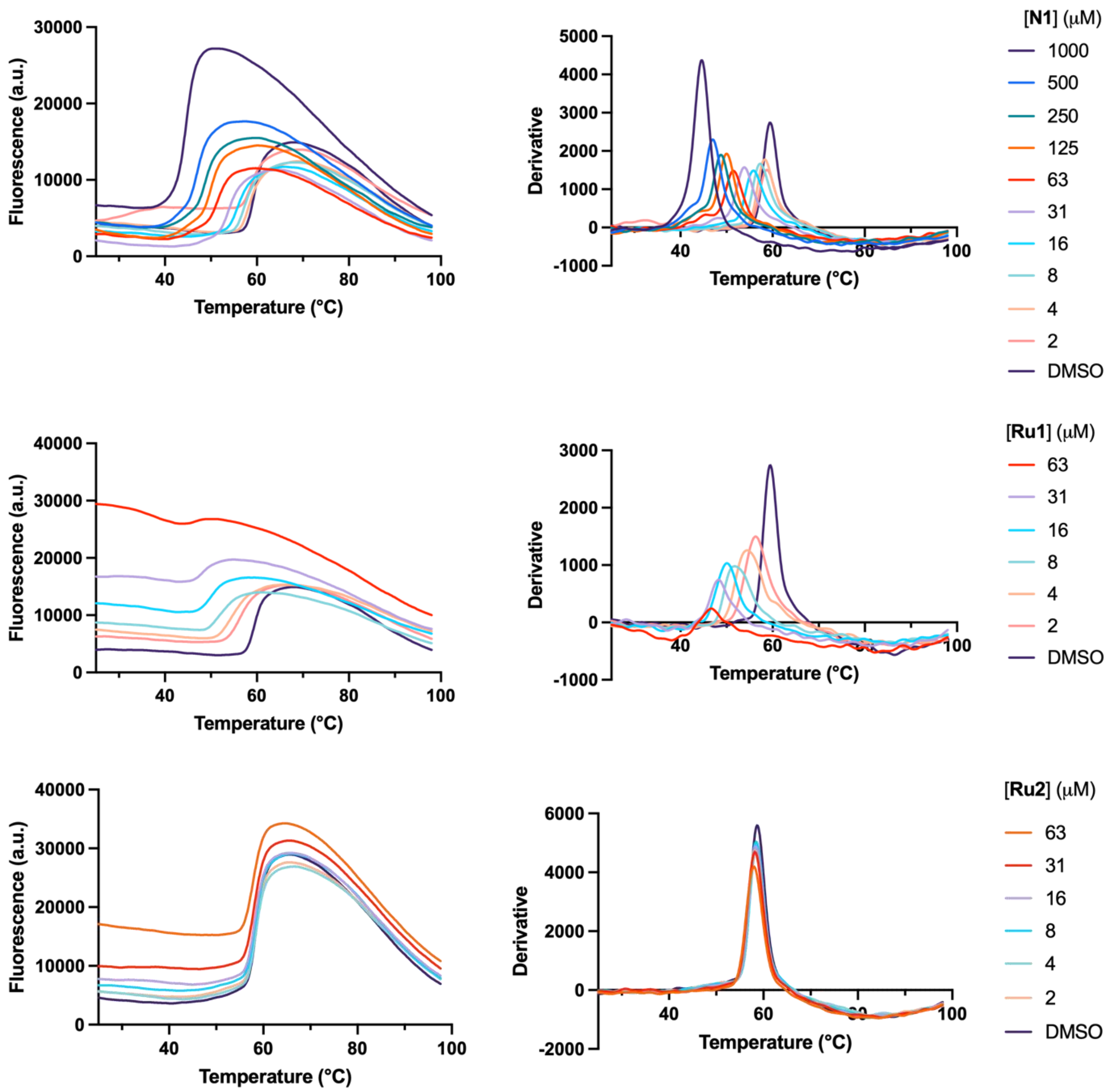

*Figure S10: Melting temperatures of NDM-1 (left) and the respective derivatives (right) with varying concentrations of **N1** (top), **Ru1** (middle), and **Ru2** (bottom). Plotted as mean-only with n = 3..*

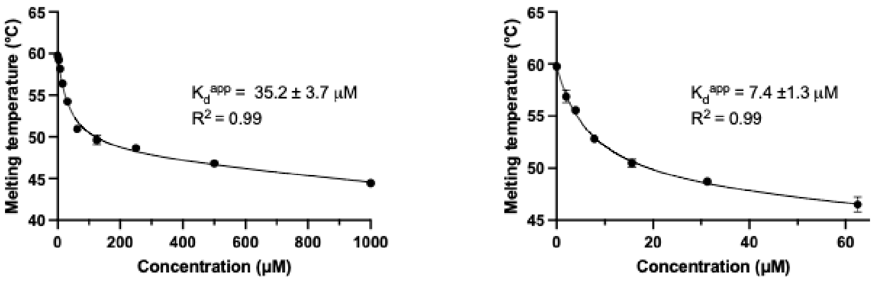

Figure S11: Apparent Kd values for N1 (left) and Ru1 (right) derived from the dose-dependent relationship of ΔTm versus concentration.

1.5 Steady State Enzyme Kinetics

The steady-state parameters of NDM-1 for the β-lactam nitrocefin were determined using an absorbance-based assay. NDM-1 activity was measured by detecting the NDM-1 catalysed

hydrolysis of nitrocefin. The reaction product results in an increase in absorbance at 468 nm. Nitrocefin concentrations were 0, 1.3, 2.6, 5.2, 10.4, 20.8, 41.6, 83, 166 and 333 µM, NDM-1 concentration was fixed at 2 nM. The assays were performed at 25 ºC in buffer containing 50 mM Tris/HCl pH 7.5, 200 mM NaCl, 5 %(v/v) glycerol, 1 mM TCEP. Activity was recorded for 100 s. The linear region of the reaction progress curves were used to determine the initial velocity (v_o) in units of µM/s product formation and converted to k_{cat} (v) by dividing by NDM-1 concentration. k_{cat} values were plotted against nitrocefin concentration and fit to the Michaelis-Menten equation using GraphPad Prism 10.2.3.

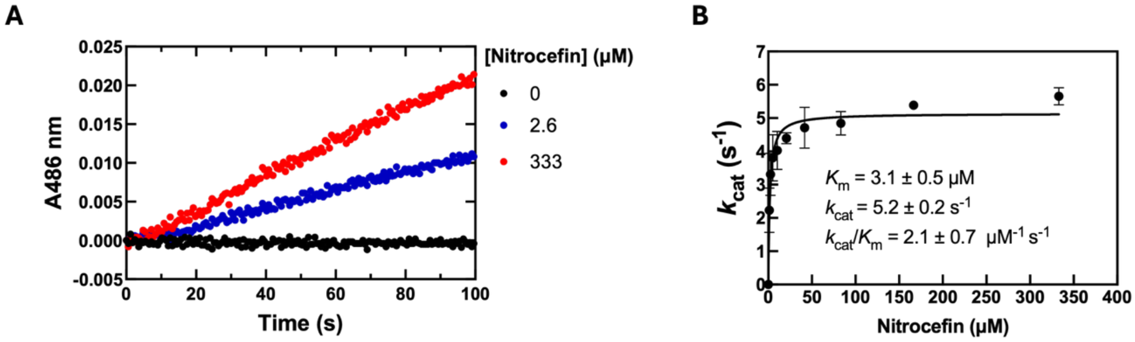

Figure S12: (A) Reaction progress curves of NDM-1 catalysed hydrolysis of nitrocefin, the rate of reaction increases with substrate concentration. (B) Representative Michaelis-Menten plot used to determine the steady-state parameters. NDM-1 has high affinity for nitrocefin with a Km(Nitrocefin) of 3.1 ± 0.5 µM, which agrees exactly with the previously reported NDM-1 Km(Nitrocefin) value of 3.2 µM,[5] the paper does not report kcat or kcat/Km values. NDM-1 concentration was 2 nM, nitrocefin concentrations were 0, 1.3, 2.6, 5.2, 10.4, 20.8, 41.6, 83, 166 and 333 µM. The assays were performed in buffer containing 50 mM Tris/HCl pH 7.5, 200 mM, NaCl, 5 %(v/v) glycerol, 1 mM TCEP at 25 ℃. Error bars represent standard deviation with n = 3.

1.6 Enzyme Inhibition Assay

1.6.1 Inhibition of recombinant NDM-1 *in vitro*

For the determination of IC_{50} values of inhibitors against NDM-1, a chromogenic assay using nitrocefin was employed. All preparations of the assay were carried out under red light (dark conditions).

Buffer A: 50 mM HEPES pH 7.5 with 0.01% Triton X-100

Buffer B: 50 mM HEPES pH 7.5 with 0.01% Triton X-100 and 10% DMSO

Inhibitor stock solutions were prepared at 10 mM in DMSO and then used to prepare the sub stock solution (1 mM compound in buffer A, 90 µL) in the first well of a Greiner 96 well plate. This sub stock solution was then serially diluted 3-fold (30 µL into 60 µL of buffer B) along the long edge of the plate to provide 12 concentrations (1 mM – 5.6 nM). 10 µL of each compound dilution was then transferred to a Greiner half-area 96 well plate in triplicate with one row of Buffer B as no-inhibitor controls (providing four rows in total). Enzyme stock (10 nM, 40 µL, final concentration of enzyme in well = 4 nM) was added to each well and the samples then pre-incubated for 10 min under either 'dark' or 'light' conditions at room temperature (25 °C) before measurement. Under 'dark' conditions, the plate was left covered at room temperature for 10 min. Under 'light' conditions the plate was left covered in the dark for 6 min 30s, followed by irradiation (208 s, 96 mW cm^{-2}).

The reaction was measured using a BMG LABTECH CLARIOstar Plus plate reader. The reaction was initiated by injection of substrate (50 µL, 200 µM, final concentration of substrate in well = 100 µM) and the plate shaken at 300 rpm for 5 s before the first reading. The samples were then measured at a discrete wavelength of 486 nm for approximately 20 cycles until the substrate had been completely consumed in the no-inhibitor controls. Method conditions are as follows: cycle time = 46 s, settling time = 0.5 s, number of flashes = 22. The raw data was processed using the CLARIOstar MARS software.

Table S4: Comparison of IC_{50} values in vitro for compounds in the dark and light.

	IC_{50} (dark) (µM)	IC_{50} (light) (µM)
**N1**	3.2	3.1
**Ru1**	23	0.22
**Ru2**	N/A	5.6

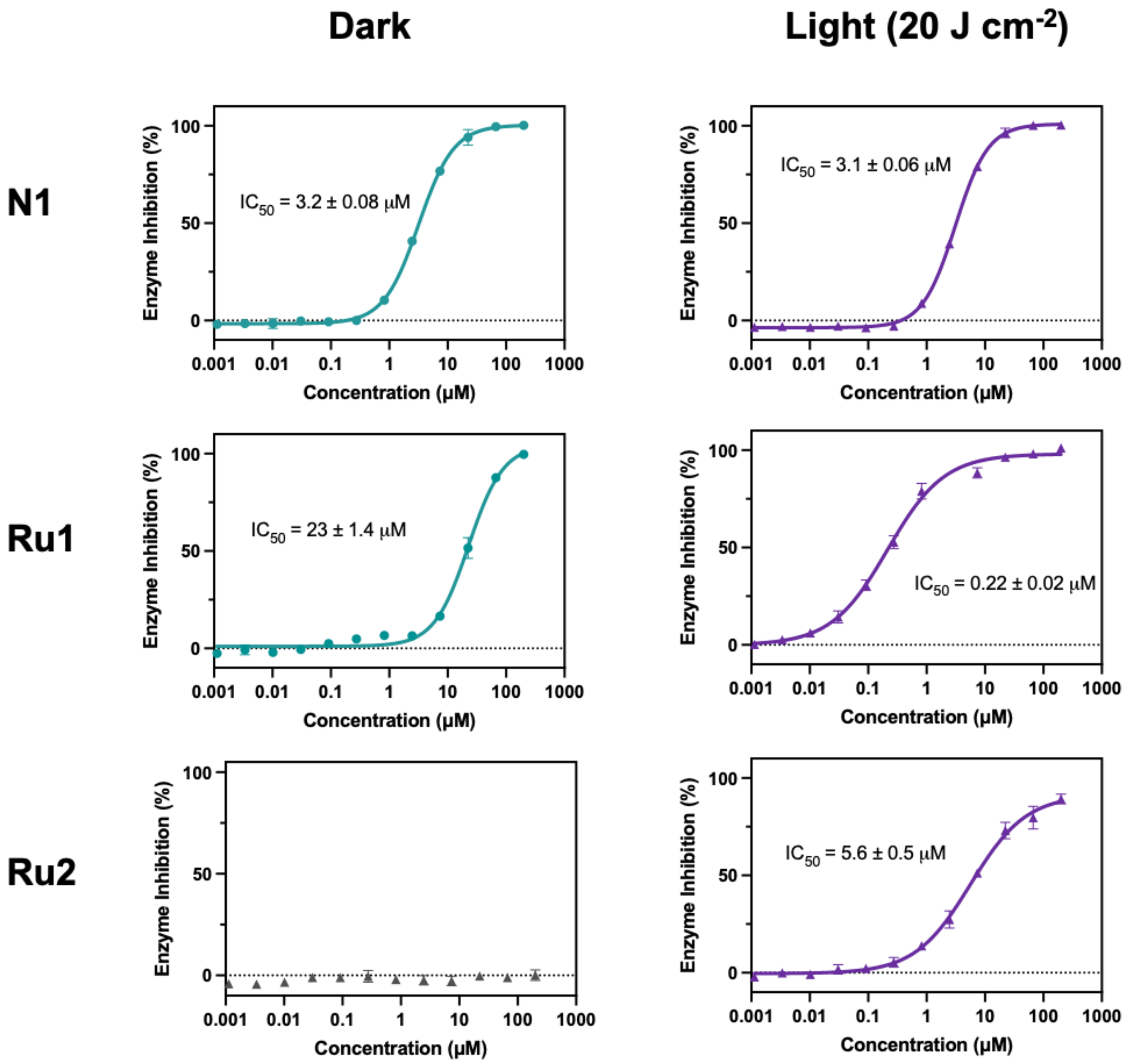

*Figure S13: Chromogenic inhibition assay results for **N1**, **Ru1**, and **Ru2** under dark, and light (450 nm 20 J cm^{-2}) conditions in vitro.*

1.6.2 Inhibition of NDM-1 in *E. coli* NDM-1

Bacteria Buffer A: PBS pH 7.5 with 0.01% Triton X-100

Bacteria Buffer B: PBS pH 7.5 with 0.01% Triton X-100 and 10% DMSO

E. coli MG1655 pSU18 NDM-1 was grown from single colonies at 37 °C with aeration, while shaking at 180 rpm for 18 hours. The overnight suspensions were subcultured (1:100) in fresh LB and incubated to yield an OD_{600} of 0.55-0.60. The were harvested and pelleted at 3500 × g for 15 minutes at 4 °C. The pellets were washed with PBS and pelleted again under the same conditions. The bacteria were then stored as pellets at 4 °C until required for the assay and to be used within 5 hours. To prepare the bacteria for the assay, a pellet was resuspended in an adjusted volume of fresh PBS to achieve an $OD_{600} \approx 5.0$ and then kept at 4 °C for 1 hr before being used. The bacteria were then diluted to $OD_{600} \approx 2.0$ in fresh PBS and used immediately.

As above for the recombinant NDM-1 assay, inhibitor stock solutions (10 mM in DMSO) were used to prepare the sub stock solution (1 mM compound in Bacteria Buffer A, 90 µL) in the first well of a Greiner 96 well plate. This sub stock solution was then serially diluted 3-fold (30 µL into 60 µL of Bacteria Buffer B) along the long edge of the plate to provide 12 concentrations (1 mM – 5.6 nM). 10 µL of each compound dilution was then transferred to a Greiner half-area 96 well plate in triplicate with one row of Bacteria Buffer B (10 µL) as a no-inhibitor control, and one row of Bacteria Buffer B (50 µL) which served as a no-bacteria control (providing five rows in total). Bacteria stock ($OD_{600} \approx 2.0$, 40 µL, final concentration of bacteria in well = $OD_{600} \approx 0.8$) was added to each well of the first four rows, the plate was sealed, and the samples then pre-incubated for 60 min under either 'dark' or 'light' conditions in a shaking incubator (180 rpm, 37 °C). Under 'dark' conditions, the plate was left sealed in a shaking incubator for 60 min. Under 'light' conditions the plate was left sealed in a shaking incubator for 50 min followed by unsealing and irradiation (624 s, 96 mW cm^{-2}, 60 J cm^{-2}). Following preincubation, injection of the substrate (50 µL, 200 µM, final concentration of substrate in well = 100 µM) was conducted manually and then the plate immediately resealed and measured.

The reaction was measured using a BMG LABTECH CLARIOstar Plus plate reader. The samples were measured at a discrete wavelength of 486 nm for up to 40 cycles or until the substrate had been completely consumed. Method conditions are as follows: cycle time = 100 s with shaking before each cycle (5 s, 400 rpm), settling time = 0.5 s, number of flashes = 22. The raw data was processed using the CLARIOstar MARS software.

Table S5: Comparison of IC_{50} values in E. coli NDM-1 for compounds in the dark and light.

	IC_{50} (dark) (µM)	IC_{50} (light) (µM)
**N1**	13.2	11.1
**Ru1**	22.4	0.75
**Ru2**	N/A	N/A

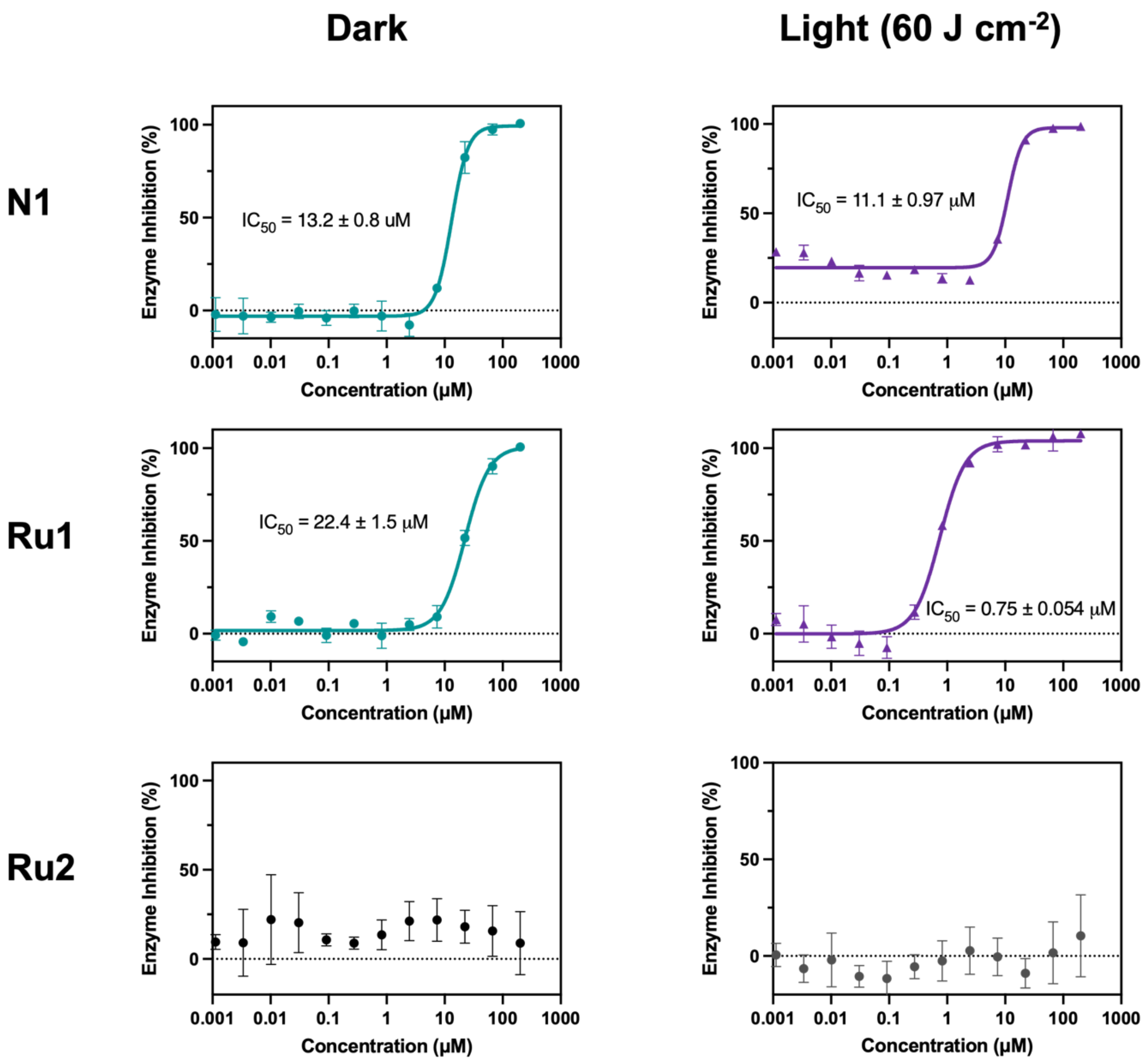

*Figure S14: Chromogenic inhibition assay results for **N1**, **Ru1**, and **Ru2** under dark, and light (450 nm 60 J cm^{-2}) conditions in E. coli NDM-1.*

1.7 HDAC Deacetylation Assay

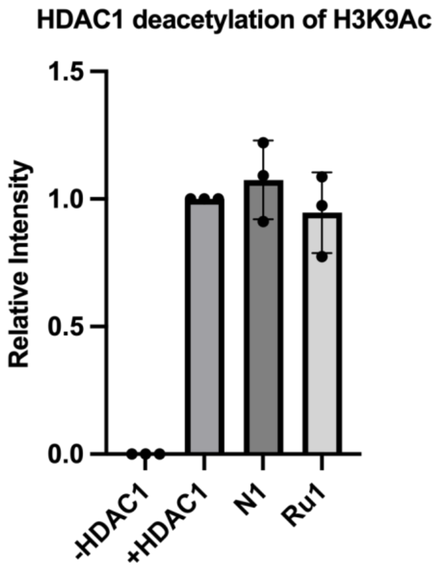

*Figure S15: **N1** and **Ru1** do not inhibit HDAC1. Deacetylation of H3K9Ac by HDAC1 was measured by mass spectrometry in the presence or absence of **N1** and **Ru1.** Error bars represent the standard deviation with n=3.*

1.7.1 HDAC1 Assay conditions:

In a reaction buffer containing 25 mM Tris-HCl (pH 8.0), 137 mM NaCl, 2.7 mM $MgCl_2$, 1 mM KCl, and 0.1 mg/mL BSA, 3 µM Histone $H3_{1-21}K9Ac$ peptide and 100 µM **Ru1** or **N1** (in DMSO) were incubated with 100 nM HDAC1 protein for 3 hours at 37°C in a total reaction volume of 10 µL or 20 µL. The reaction was quenched using a 1:1 ratio of reaction mix:1% TFA in ddH_2O. The results of the assay were analysed using HRMS.

To plot the data, product peaks were normalised to the sum of the product (H3K9) + substrate (H3K9Ac). Relative activities were calculated by normalising the product ratios to the positive control sample in the absence of **Ru1** or **N1** (+HDAC1). A negative control in the absence of HDAC1 was included (-HDAC1).

Peptide sequence:

H3K9Ac (1-21 aa, 21-mer): H-ARTKQTAR**K(Ac)**STGGKAPRKQLA-NH_2

The HDAC1 substrate peptide (H3K9Ac) was synthesised on a 0.10 mmol scale of rink amide solid resin support (Novabiochem®) using an automated Biotage® Initiator+ Alstra™ peptide synthesiser. In short, Fmoc-protected rink amide resin (0.16 g, 0.62 mmol/g loading, 0.10 mmol) was left to swell in a reaction cartridge with 10 mL DMF for 20 min at 70 °C. The resin was drained and the Fmoc group was deprotected with 20% (v/v) piperidine in DMF, stirring for 10 min at room temperature before draining. The resin was washed with DMF and then the first Fmoc-protected amino acid (4 equiv.) was coupled using DIC/Oxyma (4 equiv./ 4 equiv.) for 5 mins at 75 °C. The couplings were performed in duplicate. In the case of Fmoc-protected cysteine residue, coupling was achieved at room temperature for 1 hour. Thereafter, the resin was capped using 1 mL acetic anhydride in DMF for 10 min at room temperature. The above deprotection, double coupling, and capping steps were repeated for each amino acid in the

desired peptide sequence, except, the final amino acid which was not capped. Once the sequence was complete, the resin was washed with DCM.

Upon completion of the synthesis of the peptide sequence, the peptide was cleaved off the resin and its sidechains deprotected through treatment with cleavage cocktail K (82.5% TFA, 5% phenol, 5% MQ H_2O, 5% thioanisole, and 2.5% 1,2-ethanedithiol). After 2 hours of rocking, the cleavage cocktail was filtered through a fritted cartridge where the filtrate was collected. TFA was evaporated under a stream of nitrogen and the crude peptide was precipitated with cold diethyl ether. The suspension was centrifuged, and the supernatant was decanted. The precipitate was washed with cold diethyl ether, thrice. The residing peptide was dissolved in 50% CH_3CN in H_2O (0.1% TFA), flash frozen, and lyophilised. Peptides were purified using preparative reverse-phase high-performance liquid chromatography (prep-HPLC) on the Agilent 1260 Infinity II Quaternary HPLC with a Diode-Array Detector (DAD) for UV-vis detection at 214 nm and 280 nm, and a Zorbax 300SB-C18 column (7 µm, 21.2 x 150 mm). A gradient of CH_3CN in H_2O (0.1% TFA, 0 – 70%, 30 min) was used with a constant flow rate of 20 mL/min at room temperature. High resolution mass spectra (HRMS) were obtained using a Waters Acquity UHPLC with reverse phase chromatography paired to a Waters Xevo G2-XS QToF mass spectrometer. An Acquity UPLC BEH C18 column (1.7 µm, 21 x 50 mm) was applied with a gradient of CH_3CN in H_2O (0.1% formic acid) at a flow rate of 0.4 mL/min.

1.8 Protein degradation via SDS-PAGE

All preparations of the assay were carried out under red light conditions. Compound stocks were made at 10 mM in DMSO and then diluted to the appropriate concentration (1 mM – 10 µM) in 50 mM HEPES pH 7.5. The enzyme stocks (10 µM) were made up in 50 mM HEPES pH 7.5. The compound and enzyme stocks were mixed 1:1 and then pre-incubated for 10 min under either ‘dark’ or ‘light’ conditions at room temperature (25 °C) before measurement. Under ‘dark’ conditions, the samples are left covered at room temperature for 10 min. Under ‘light’ conditions the samples were transferred to a 96 well plate to provide irradiation. For a dose of 20 J cm^{-2} the plate was incubated in the dark for 6 min 30s, followed by irradiation (208 s, 96 mW cm^{-2}). For a dose of 60 J cm^{-2} the plate was irradiated immediately (624 s, 96 mW cm^{-2})

An aliquot of each sample was mixed with SDS-sample buffer (NuPAGE® LDS Sample buffer 4x) and heated to 95°C for 3 min. The samples were loaded on to the gel (NuPAGE® 4-12% Bis-tris gel) and run at 200V/125 mA for 30 min (1 gel per Gel box) or 60 min (2 gels per Gel box). SDS-PAGE gels were imaged using a Cytiva Amersham ImageQuant™ 800. Densitometry of gel bands was measured and processed using Fiji (ImageJ).

1.8.1 Ru1 and Ru2 (20 J cm^{-2}, 1 – 100 eq.)

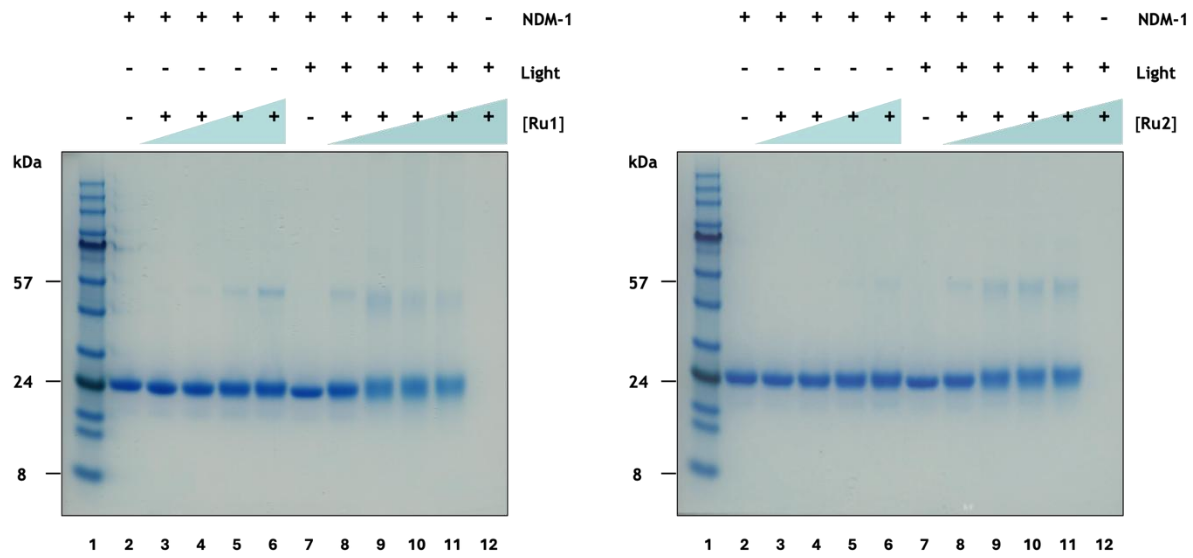

*Figure S16: SDS-PAGE gels of NDM-1 incubated with **Ru1** (left) and **Ru2** (right) under dark and light conditions (20 J cm^{-2}). Lane 1: Ladder; Lane 2: NDM-1 – no compound (dark); Lane 3: NDM-1 + 1 eq. compound (dark); Lane 4: NDM-1 + 10 eq. compound (dark); Lane 5: NDM-1 + 50 eq. compound (dark); Lane 6: NDM-1 + 100 eq. compound (dark); Lane 7: NDM-1 – no compound (light); Lane 8: NDM-1 + 1 eq. compound (light); Lane 9: NDM-1 + 10 eq. compound (light); Lane 10: NDM-1 + 50 eq. compound (light); Lane 11: NDM-1 + 100 eq. compound (light); Lane 12: 1 mM compound (light) – no protein control.*

1.8.2 Ru1 (60 J cm^{-2}, 1 – 100 eq.)

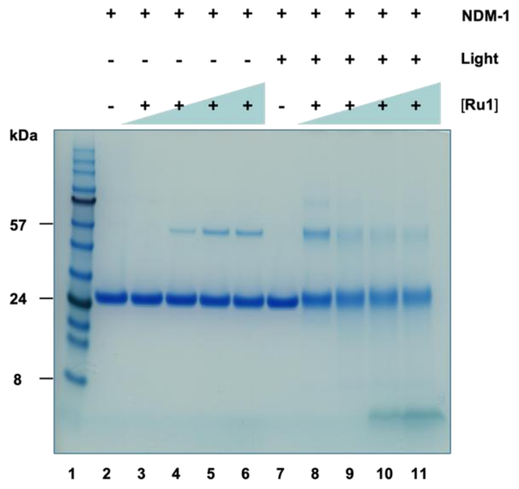

*Figure S17: SDS-PAGE gel of NDM-1 incubated with **Ru1** under dark and light conditions (60 J cm^{-2}). Lane 1: Ladder; Lane 2: NDM-1 – no compound (dark); Lane 3: NDM-1 + 1 eq. compound (dark); Lane 4: NDM-1 + 10 eq. compound (dark); Lane 5: NDM-1 + 50 eq. compound (dark); Lane 6: NDM-1 + 100 eq. compound (dark); Lane 7: NDM-1 – no compound (light); Lane 8: NDM-1 + 1 eq. compound (light); Lane 9: NDM-1 + 10 eq. compound (light); Lane 10: NDM-1 + 50 eq. compound (light); Lane 11: NDM-1 + 100 eq. compound (light).*

1.8.3 BSA+NDM-1 selectivity experiment

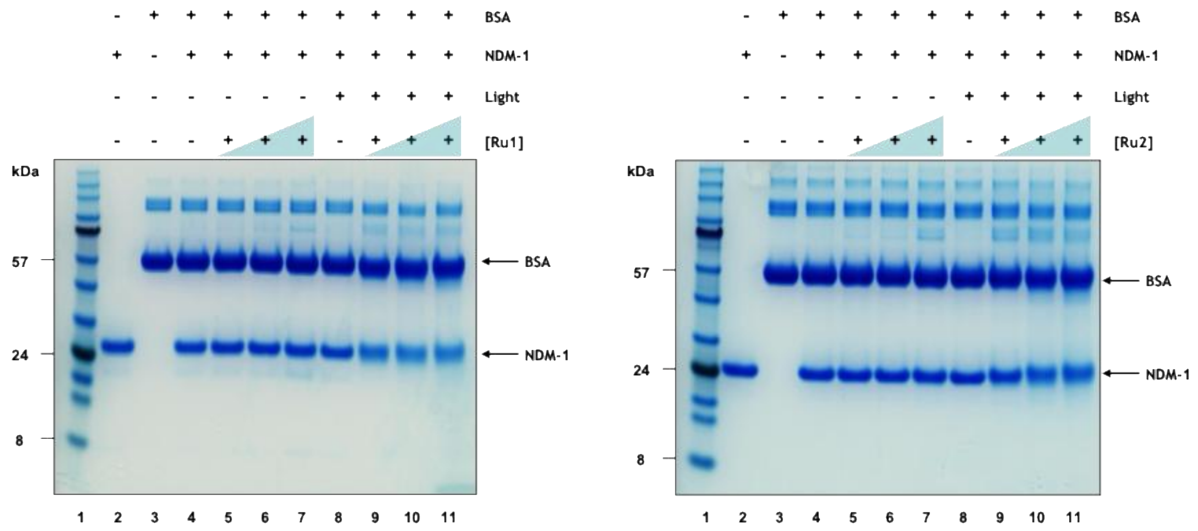

*Figure S18: SDS-PAGE gels of NDM-1 + BSA incubated with **Ru1** (left) and **Ru2** (right) under dark and light conditions (450 nm, 20 J cm^{-2}). Lane 1: Ladder; Lane 2: NDM-1 – no compound control (dark); Lane 3: BSA – no compound control (dark); Lane 4: NDM-1 + BSA - no compound control (dark); Lane 5: NDM-1 + BSA + 1 eq. compound (dark); Lane 6: NDM-1 + BSA + 10 eq. compound (dark); Lane 7: NDM-1 + BSA + 100 eq. compound (dark); Lane 8: NDM-1 + BSA - no compound control (light); Lane 9: NDM-1 + BSA + 1 eq. compound (light); Lane 10: NDM-1 + BSA + 10 eq. compound (light); Lane 11: NDM-1 + BSA + 100 eq. compound (light).*

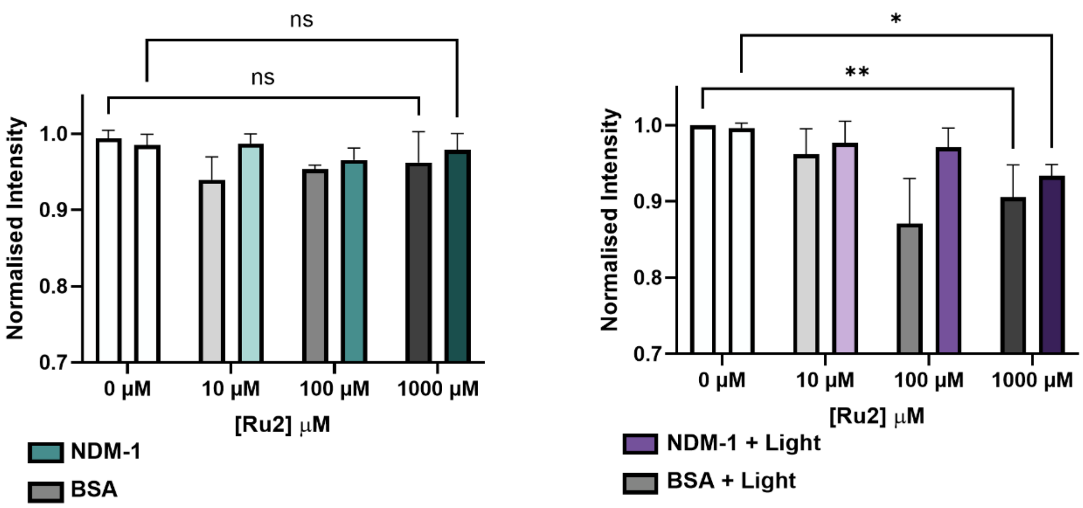

*Figure S19: Results of SDS-PAGE densitometry analysis of a mixture of NDM-1 and BSA treated with **Ru2** (0 - 1000 µM) in the dark (left) and under light irradiation (450 nm, 20 J cm^{-2}). A small amount of indiscriminate degradation of NDM-1 and BSA is observed. Error bars represent standard deviation with n = 3. ns (not significant) = $P > 0.05$, * = $P \leq 0.05$, ** = $P \leq 0.01$.*

1.9 Liquid Chromatography-Mass Spectrometry (LC-MS)

1.9.1 Intact Mass Spectrometry

Samples were prepared as per the protocol for SDS-PAGE to provide stock solutions containing **Ru1** and 5 µM protein. The protein was then precipitated with methanol/chloroform using a previously reported procedure;[6] Four volumes of methanol were added to one volume of the protein sample, and the mixture was vortexed. One volume of chloroform was then added, and the mixture was vortexed. Three volumes of water were added, the sample was vortexed and then centrifuged at 10000 g for 5 min. The aqueous methanol layer was removed from the top of the sample. The proteins remained at the phase boundary between the aqueous methanol layer and the chloroform layer. Four volumes of methanol were added, and the mixture was vortexed. The sample was spun at 15000 g for 5 min. The supernatant was removed without disturbing the pellet, and the pellet was air dried. The dried pellet was then dissolved in 100 µL H_2O (2% formic acid (v/v) + 2% CH_3CN (v/v)). Samples were measured using a Waters Xevo G2-XS QTof equipped with a Acquity Premier Protein BEH C4 300 Å 1.7 µm 2.1 x 50 mm column using a gradient of H_2O/CH_3CN (+ 0.1% formic acid) 95–5% over 8 min. The multiply charged ESI+ spectra were deconvoluted using the MaxEnt1 algorithm within MassLynx.

NDM-1 + no-compound control (dark)

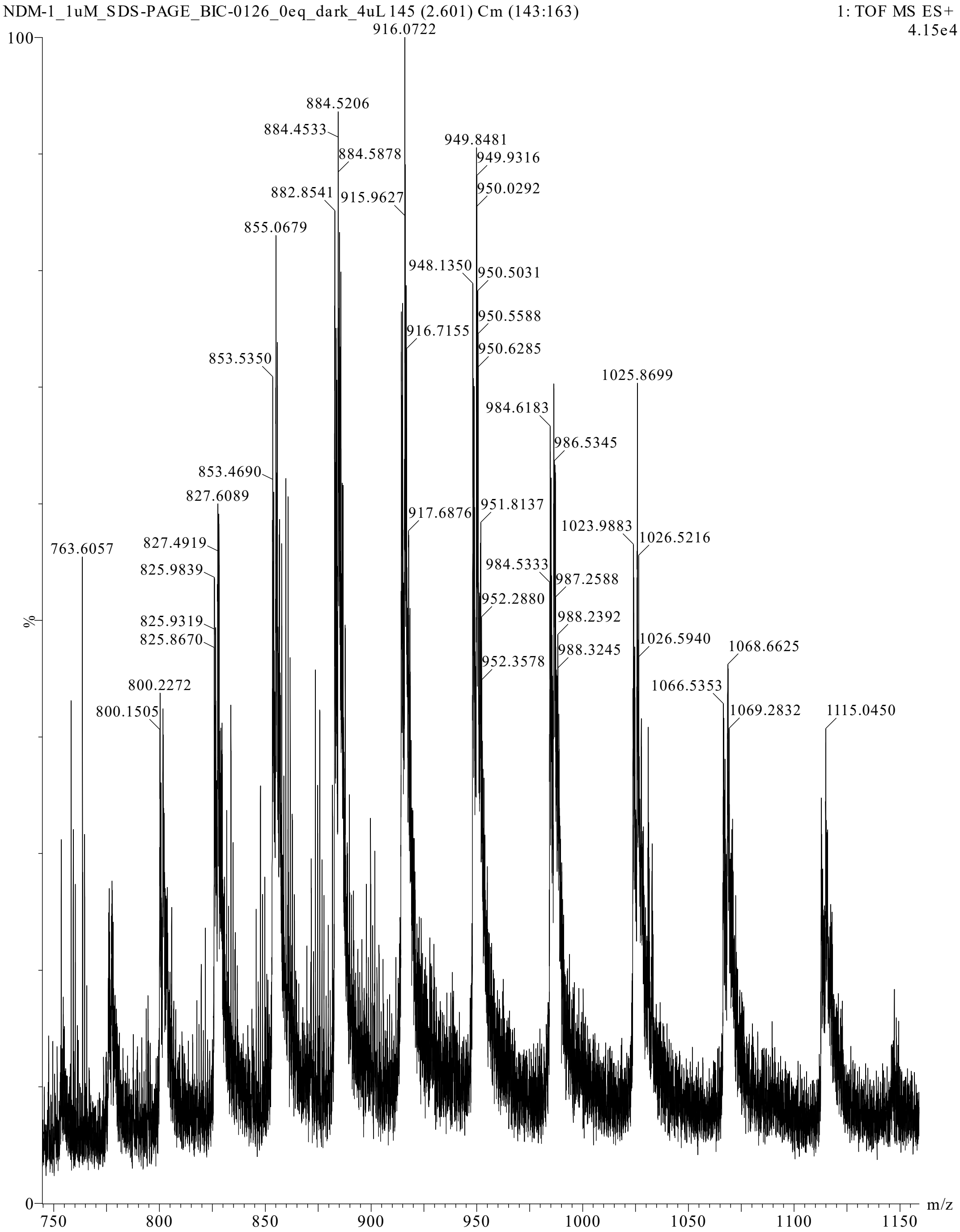

Figure S20: Raw mass spectrum of NDM-1 in the dark with no inhibitor.

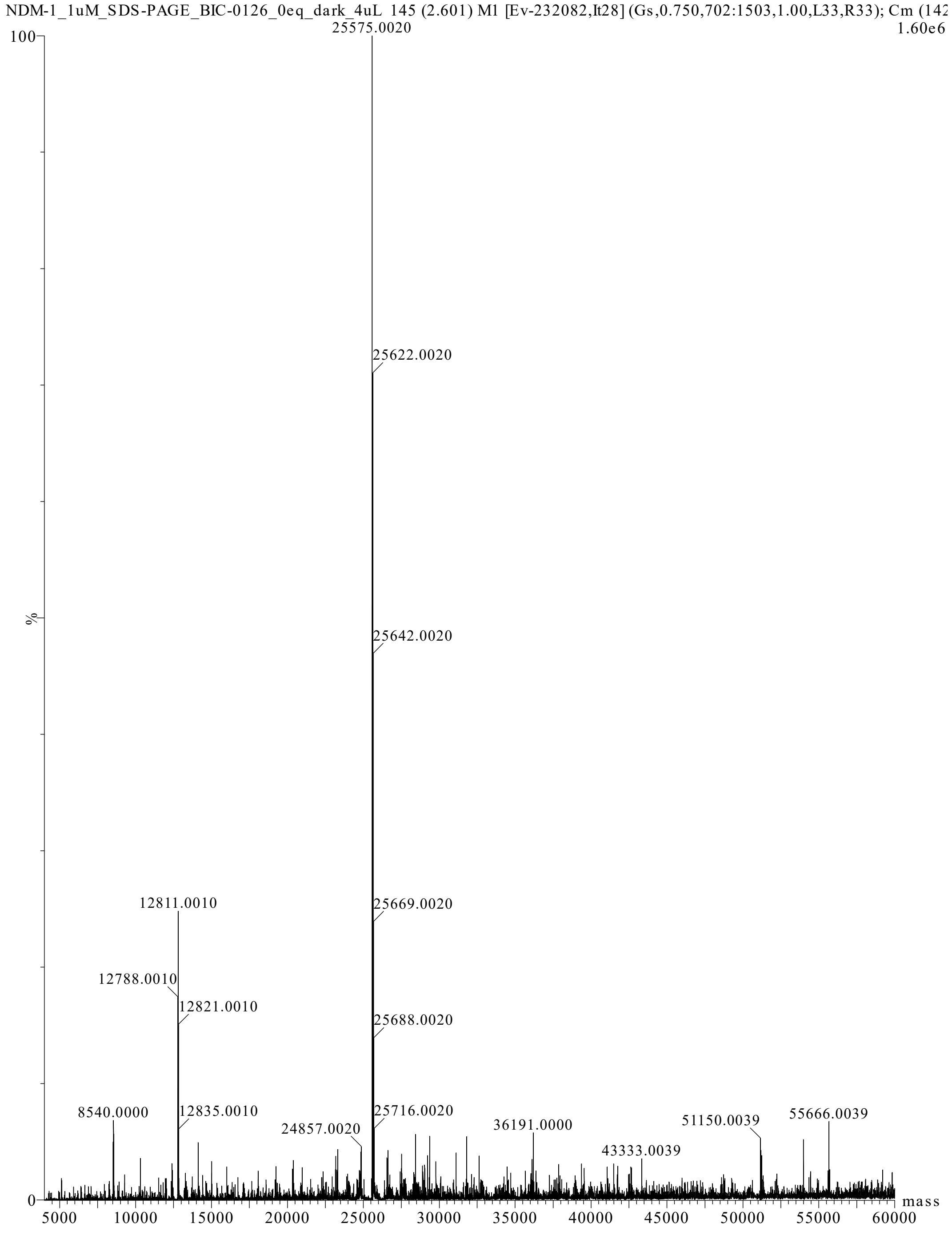

Figure S21: Deconvoluted mass spectrum of NDM-1 in the dark with no inhibitor.

NDM-1 + no-compound control (light, 20 J cm^{-1})

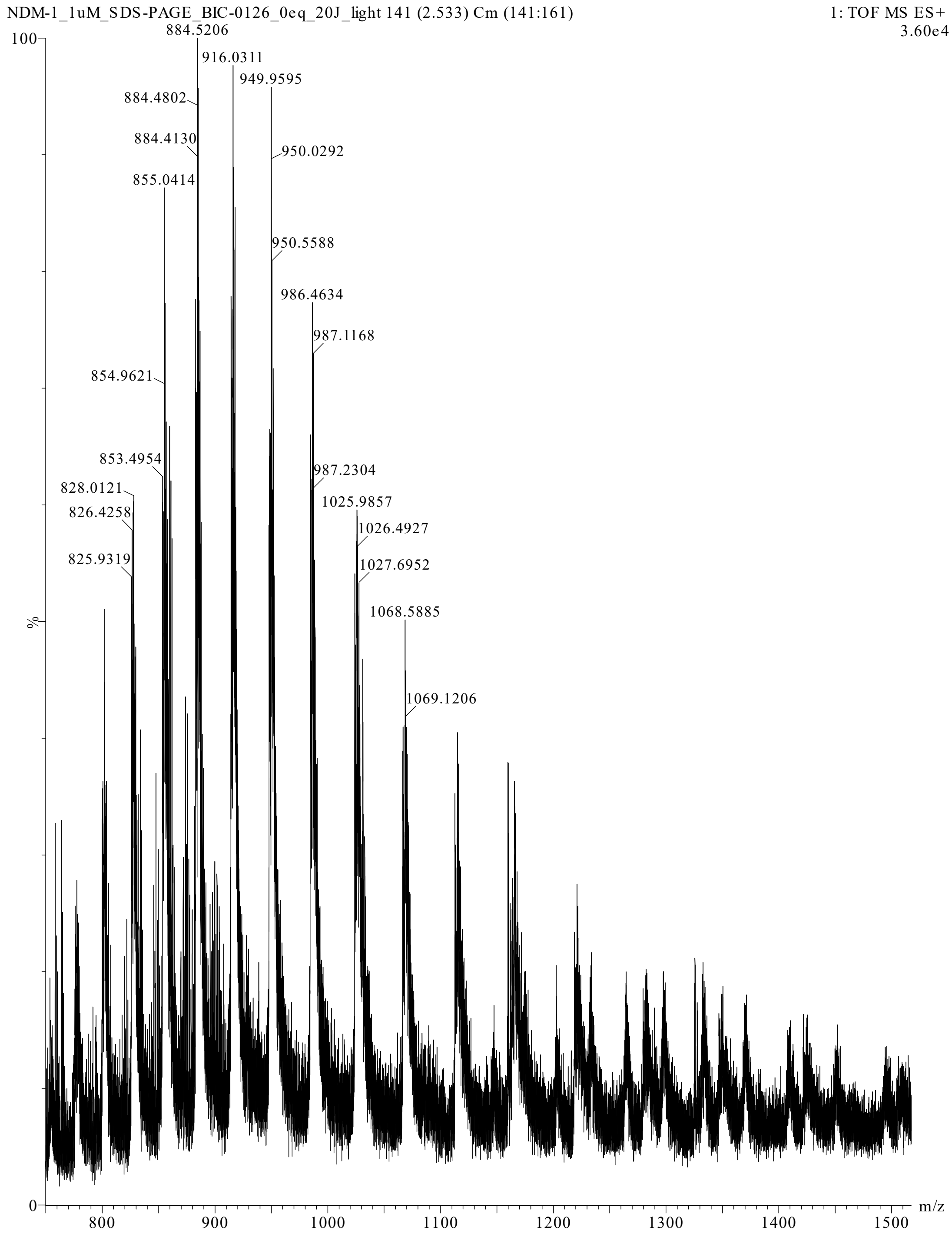

Figure S22: Raw mass spectrum of NDM-1 following light irradiation (450 nm, 20 J cm^{-2}) with no inhibitor.

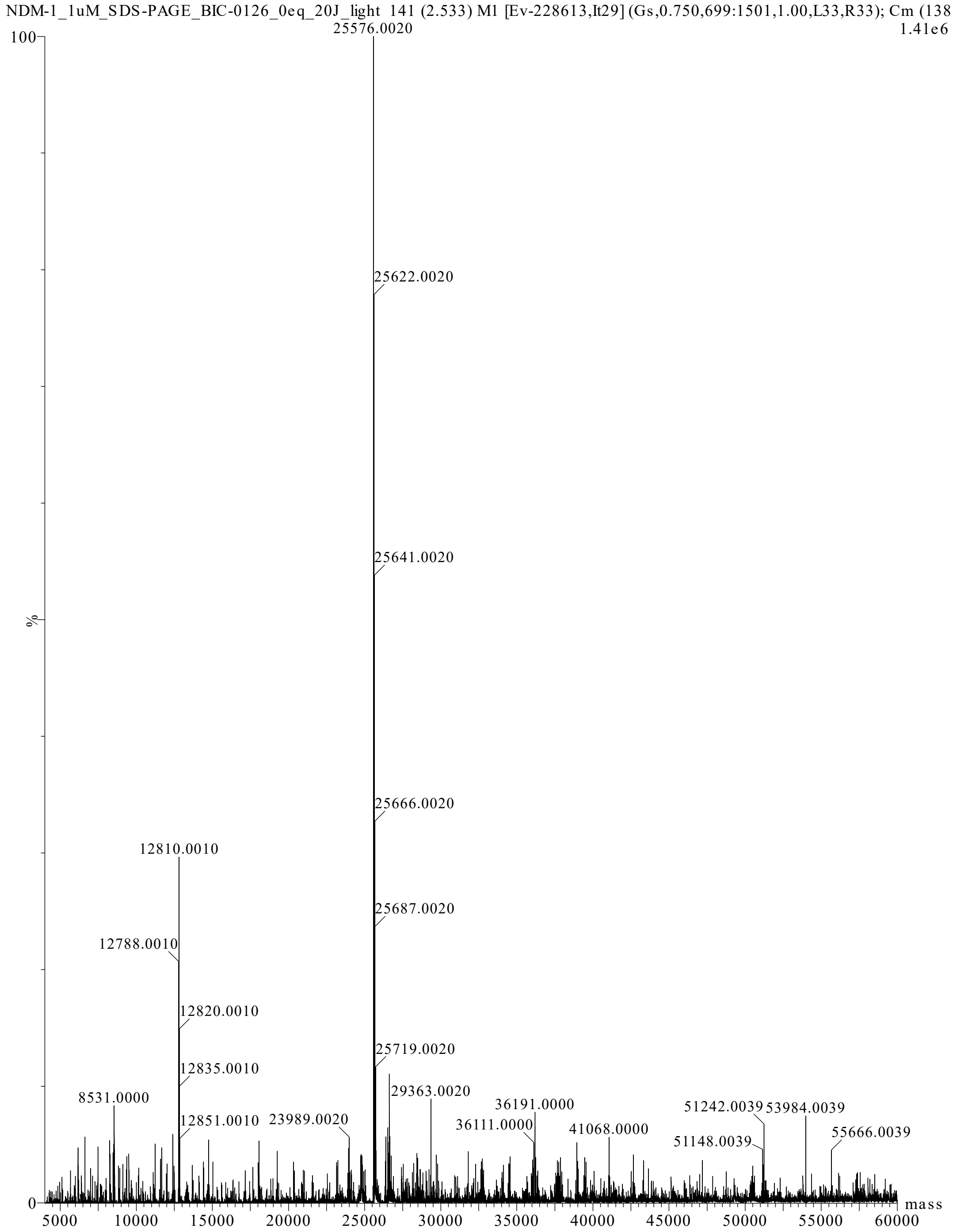

Figure S23: Deconvoluted mass spectrum of NDM-1 following light irradiation (450 nm, 20 J cm^{-2}) with no inhibitor.

NDM-1 + 100 eq. Ru1 (dark)

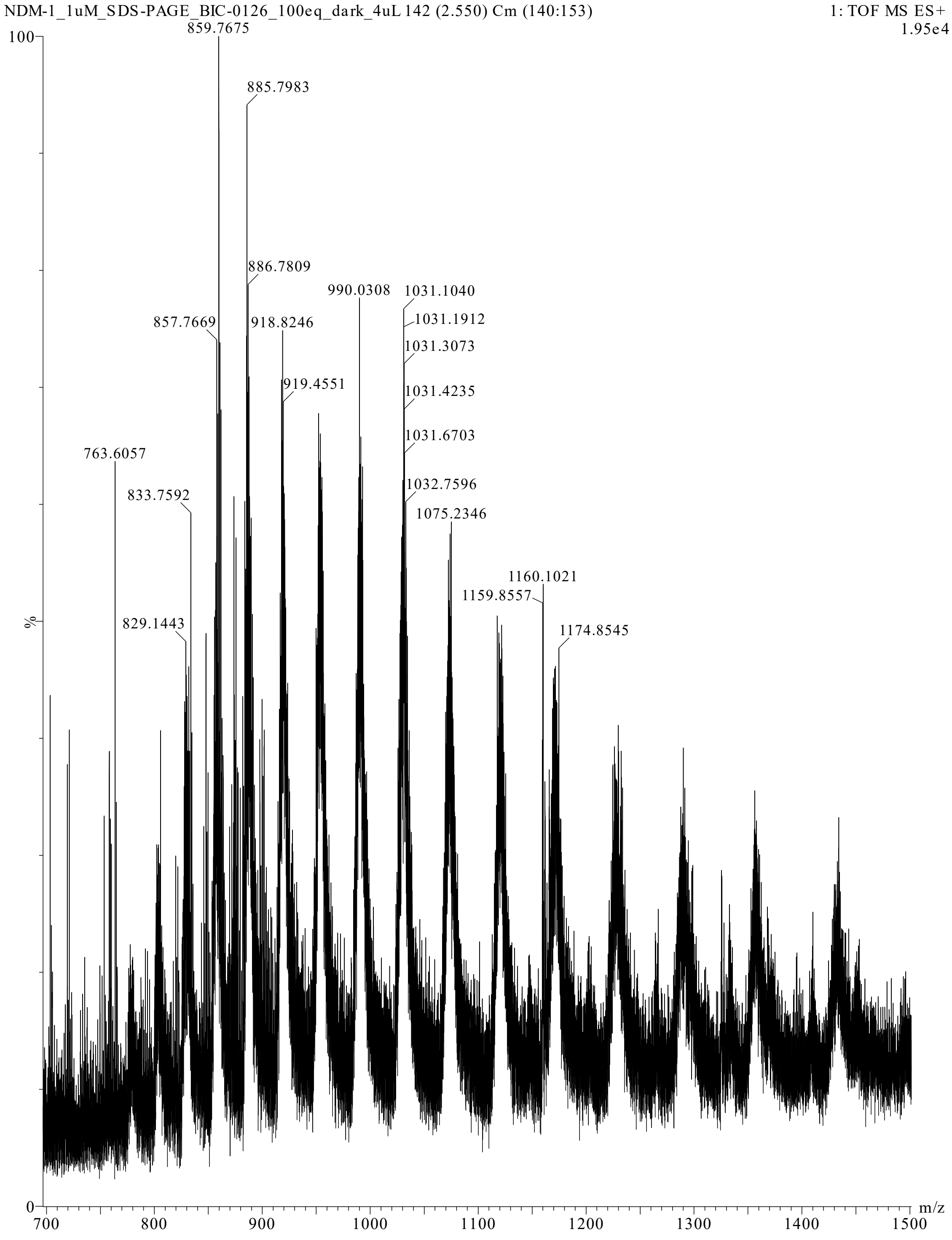

*Figure S24: Raw mass spectrum of NDM-1 in the dark following incubation with **Ru1** (100 equiv.).*

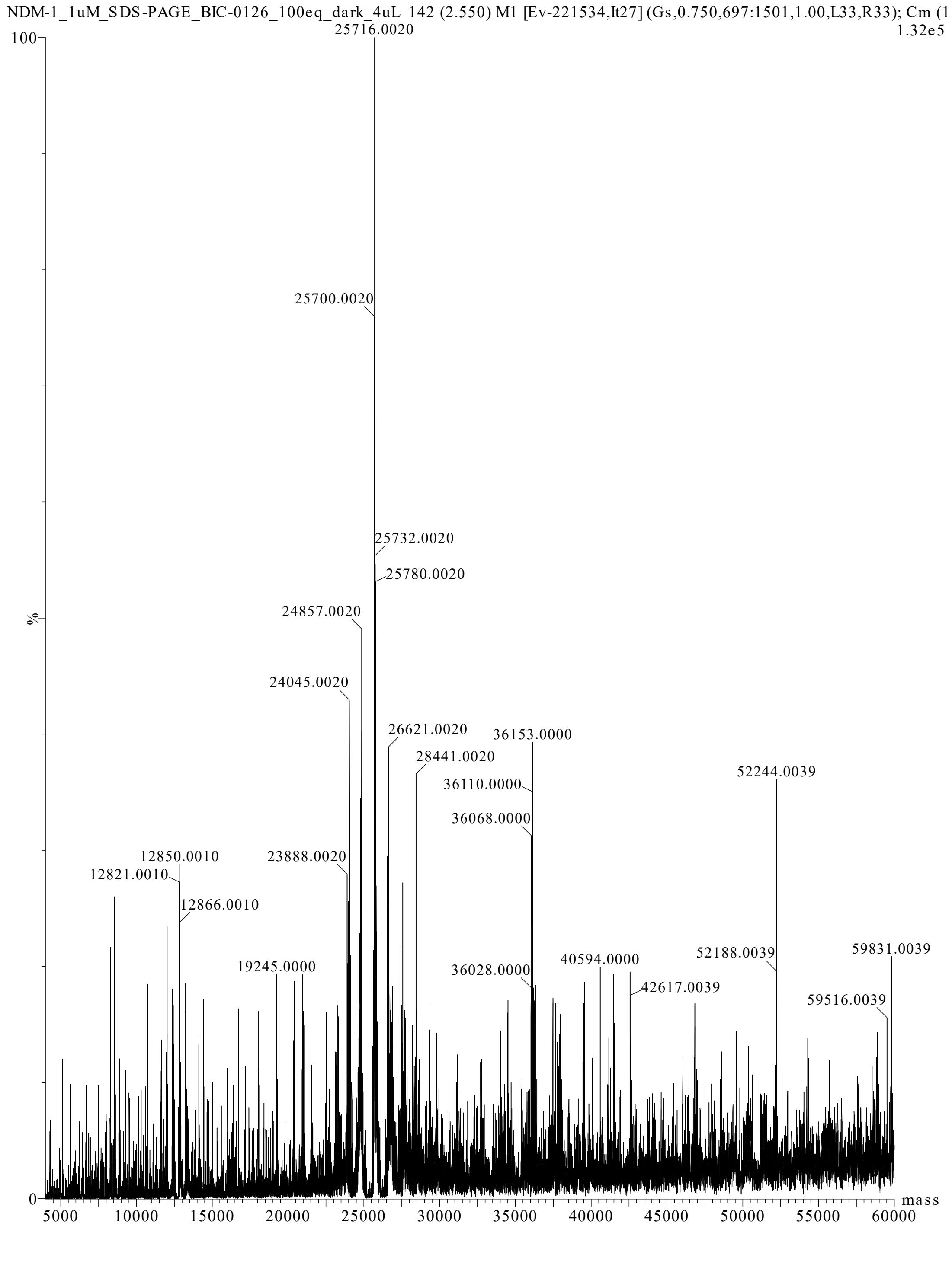

*Figure S25: Deconvoluted mass spectrum of NDM-1 in the dark following incubation with **Ru1** (100 equiv.).*

NDM-1 + 1 eq. Ru1 (light, 20 J cm^{-1})

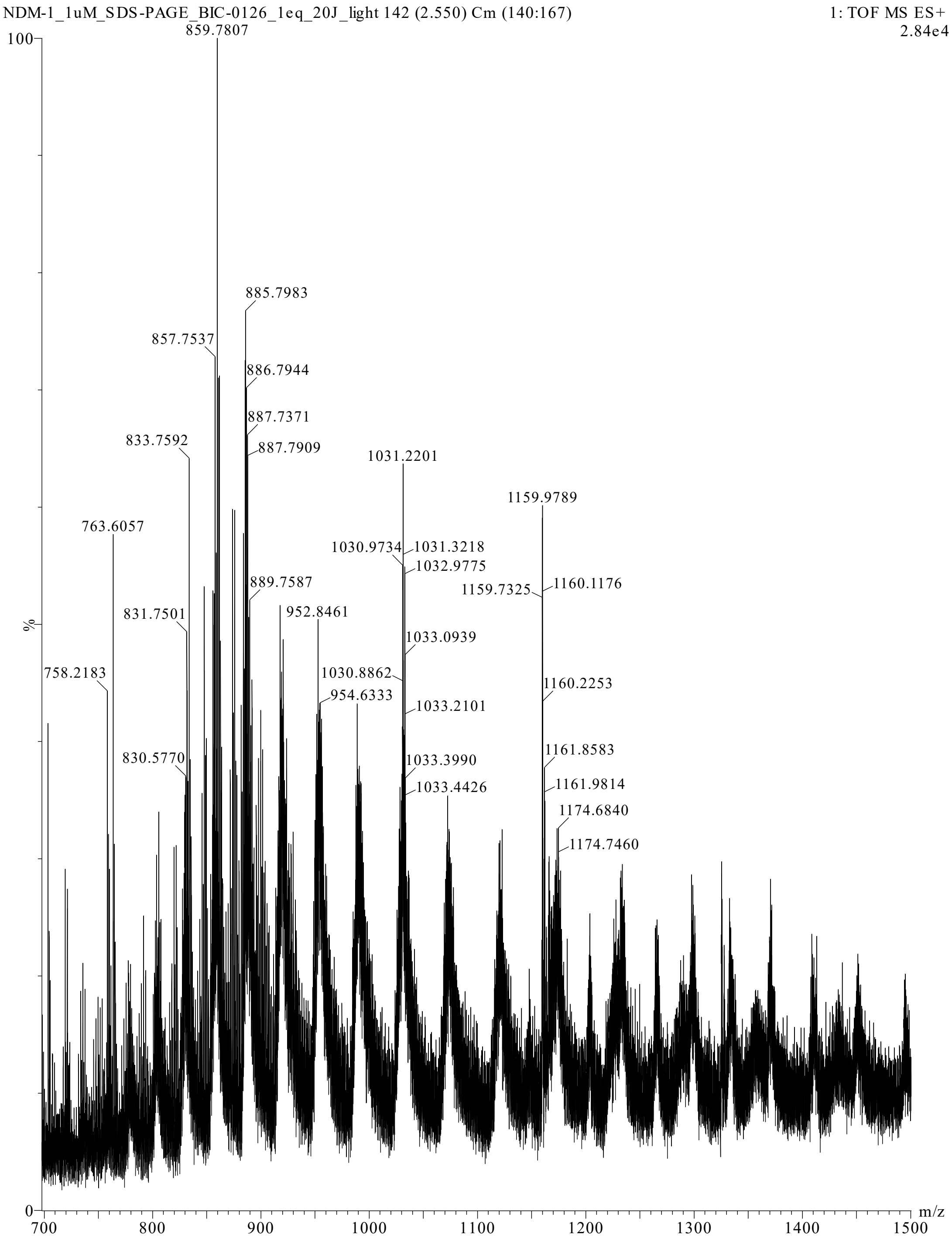

*Figure S26: Raw mass spectrum of NDM-1 following light irradiation (450 nm, 20 J cm^{-2}) and incubation with **Ru1** (1 equiv.).*

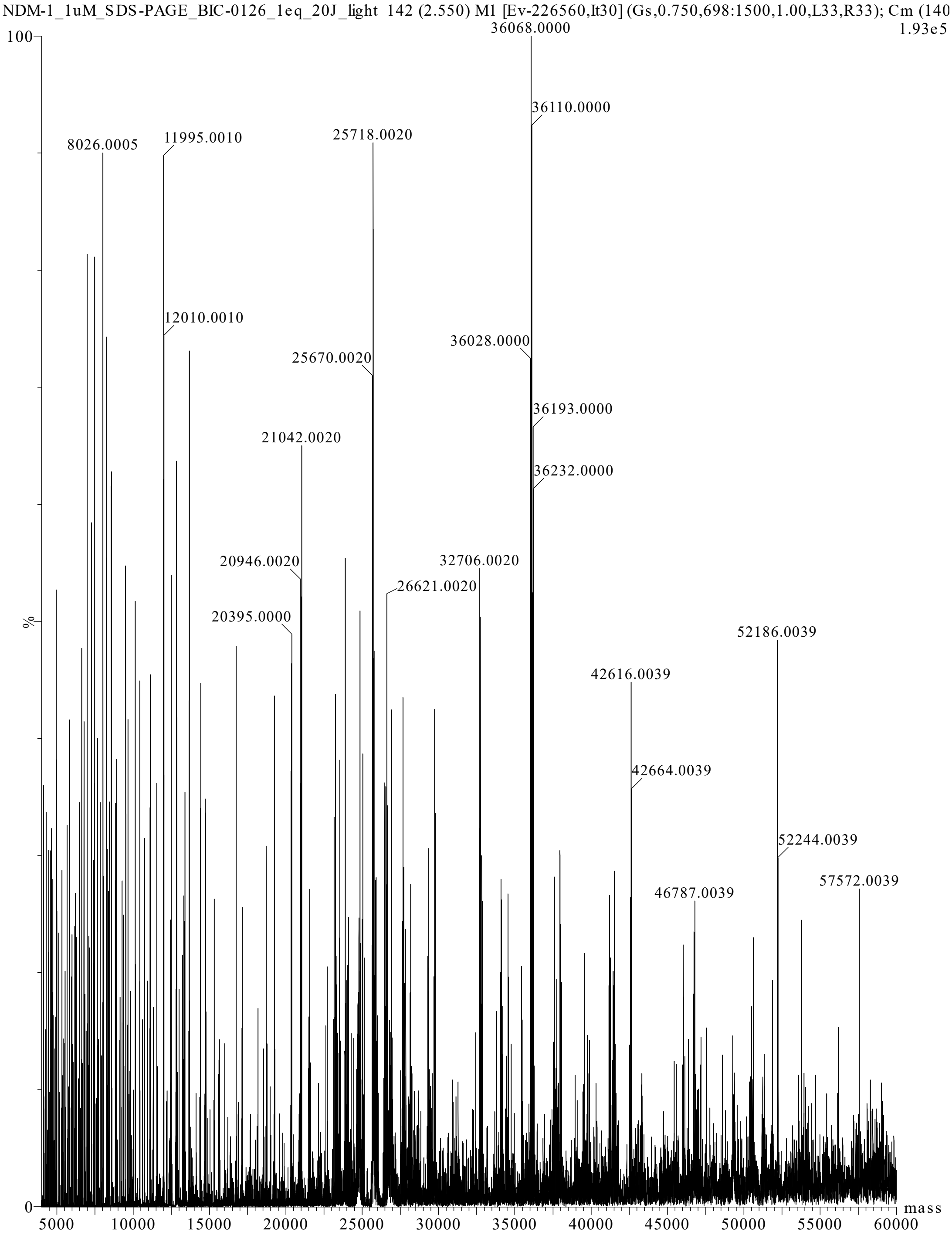

*Figure S27: Deconvoluted mass spectrum of NDM-1 following light irradiation (450 nm, 20 J cm^{-2}) and incubation with **Ru1** (1 equiv.).*

1.9.2 Trypsin Digest Mass Spectrometry

Proteins were reduced with 10 mM dithiothreitol and alkylated with 50 mM iodoacetamide. They were then digested with trypsin at an enzyme-to-substrate ratio of 1:100 for 2 h at room temperature and further digested overnight at 37°C following addition of trypsin at a ratio of 1:20.

Peptides were diluted in 1% (v/v) formic acid and 2% (v/v) CH_3CN and analysed by nano-scale capillary LC-MS/MS using a Vanquish Neo UPLC (ThermoScientific Dionex, USA) to deliver a flow of approximately 250 nL/min. A PepMap Neo C18 5 µm, 300 µm x 5 mm nanoViper (ThermoScientific Dionex, USA) trapped the peptides before separation on a 50 cm EASY-Spray column (50 cm x 75 µm ID, PepMap C18, 2 µm particles, 100 Å pore size: ThermoScientific, USA). Peptides were eluted with a gradient of CH_3CN over 30 min. The analytical column outlet was directly interfaced *via* a nano-flow electrospray ionisation source, with a quadrupole Orbitrap mass spectrometer (Orbitrap Exploris 480, ThermoScientific, USA). MS data were acquired in data-dependent mode using a top 10 method, where ions with a precursor charge state of 1+ was excluded. High-resolution full scans (R=60000, *m/z* 380–1600) were recorded in the Orbitrap followed by higher energy collision dissociation (HCD) using normalized collision energy of 30% for the 10 most intense MS peaks. The fragment ion spectra were acquired at a resolution of 15,000 and a dynamic exclusion window of 20 s was applied.

For data analysis, Xcalibur raw files were converted into the MGF format using Mascot Distliler (Matrix Science) and used directly as input files for Mascot.[7] Searches were performed against an *ad hoc* protein database containing the sequence of the proteins in the complex. The following parameters were set for the searches: maximum number of missed cleavages = 3; minimum peptide length = 5 amino acids; variable modifications = carbamidomethylation of cysteine (mass shift 57.02146 Da), methionine oxidation (mass shift 15.99491 Da, highlighted in green). Finally, each fragmentation spectrum was manually inspected and validated.

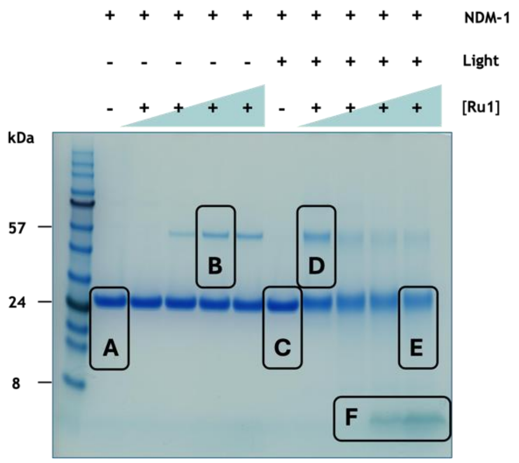

*Figure S28: SDS-PAGE gel of NDM-1 incubated with **Ru1** under dark and light conditions (60 J cm^{-2}). Gel bands cut out for trypsin digest LC-MS/MS have been highlighted.*

Band A: NDM-1 + no-compound control (dark)

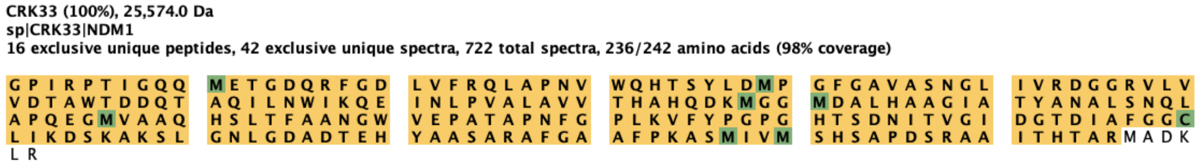

Figure S29: Peptide sequence coverage of NDM-1 following trypsin digest of band A, extracted from Scaffold 5 Proteome software. Yellow indicates 80 – 94% probability, white indicates 0 – 19% probability and green M's indicate methionine oxidation.

Band B: NDM-1 + 50 eq. Ru1 (dark)

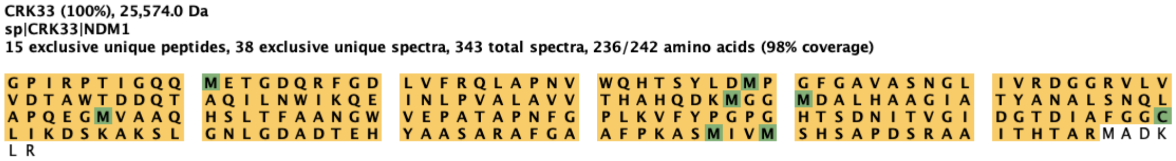

Figure S30: Peptide sequence coverage of NDM-1 following trypsin digest of band B, extracted from Scaffold 5 Proteome software. Yellow indicates 80 – 94% probability, white indicates 0 – 19% probability and green M's indicate methionine oxidation.

Band C: NDM-1 + no-compound control (light)

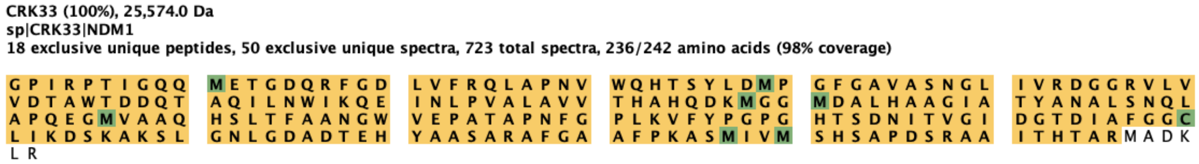

Figure S31: Peptide sequence coverage of NDM-1 following trypsin digest of band C, extracted from Scaffold 5 Proteome software. Yellow indicates 80 – 94% probability, white indicates 0 – 19% probability and green M's indicate methionine oxidation.

Band D: NDM-1 + 1 eq. Ru1 (light)

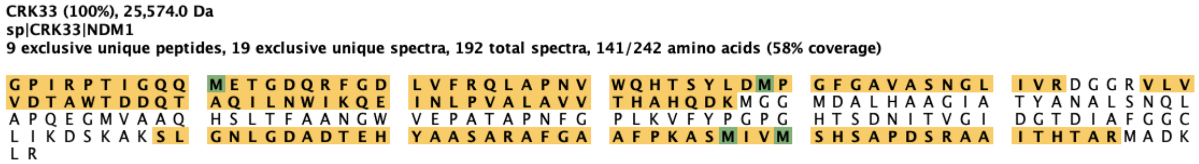

Figure S32: Peptide sequence coverage of NDM-1 following trypsin digest of band D, extracted from Scaffold 5 Proteome software. Yellow indicates 80 – 94% probability, white indicates 0 – 19% probability and green M's indicate methionine oxidation.

Band E: NDM-1 + 100 eq. Ru1 (light)

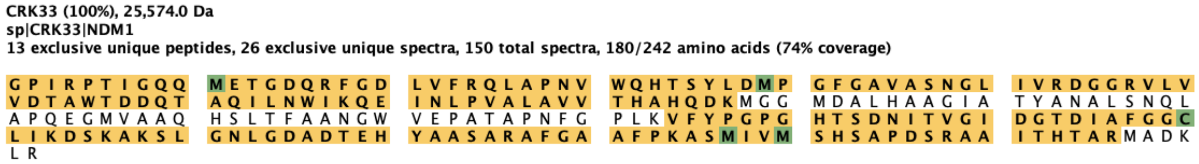

Figure S33: Peptide sequence coverage of NDM-1 following trypsin digest of band E, extracted from Scaffold 5 Proteome software. Yellow indicates 80 – 94% probability, white indicates 0 – 19% probability and green M's indicate methionine oxidation.

Band F: NDM-1 + 50/100 eq. Ru1 (light)

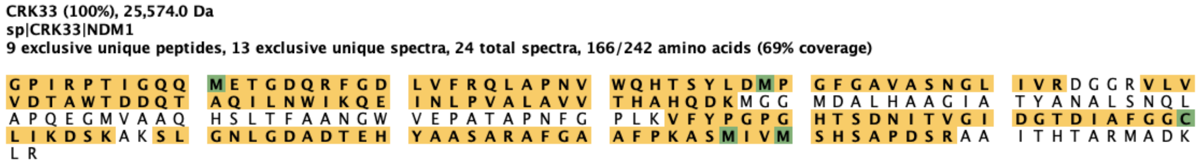

Figure S34: Peptide sequence coverage of NDM-1 following trypsin digest of band F, extracted from Scaffold 5 Proteome software. Yellow indicates 80 – 94% probability, white indicates 0 – 19% probability and green M's indicate methionine oxidation.

1.10 Molecular Docking Studies

Docking calculations were performed using MetalDock.[8] The **Ru1** ligand input was generated by editing the tris(2,2'-bipyridyl)ruthenium(II) crystal structure (CSD refcode BPYRUF) in Discovery Studio Visualizer. Geometry optimization of **Ru1** within the MetalDock workflow employed ORCA 6.0.1 with the Hay–Wadt effective core potential basis set for ruthenium; the optimized complex was treated with a total charge of +2 and spin multiplicity of 0. NDM-1 was prepared from PDB entry 6RMF by removing the co-crystallized ligand and all water molecules; the protein was then protonated at physiological pH (7.4) and Gasteiger partial charges were assigned to all atoms. A cubic grid box (30 Å per side) was centered on the two active-site Zn(II) ions. Fifty docking poses were generated and clustered; the cluster exhibiting the lowest (best) docking score and highest population was retained for further analysis. Final binding modes were visualized in PyMOL.

1.11 Confocal Microscopy

All work with dyes performed under red light conditions and samples protected from light at all times. Cultures of *E. coli* MG1655 pSU18 empty and *E. coli* MG1655 pSU18 NDM-1 were prepared by inoculating 12.5 mL Müller Hinton broth II (MHBII) in 2 x 125 mL Erlenmeyer flasks. The flasks were then incubated at 37 °C with shaking (180 rpm) for 16 h. The resultant cultures had an optical density at 600 nm (OD_{600}) of 7.7 (empty) and 6.2 (NDM-1). The suspensions were diluted in 75-fold in MHBII (167 µL in 12.5 mL) and the resultant suspensions incubated for a further 2 h at 37 °C with shaking (180 rpm). Dilutions had an OD_{600} of 0.7 (empty) and 0.8 (NDM-1) after 2 h. Both cultures were diluted 2-fold to give 10 mL cultures with an OD_{600} of 0.4. 4 x 1.5 mL Eppendorf's were primed with each strain to give 8 total cultures. The analytes (**Ru1** and **Ru2**) and the control (3-[[(7-Hydroxy-2-oxo-2H-1-benzopyran-3-yl)carbonyl]amino]-D-alanine hydrocholoride (HADA)) were taken as 10 mM stocks in DMSO and samples were prepared at 100 µM for **Ru1**, **Ru2** and HADA. The

following samples were prepared: 1: *E. coli* MG1655 pSU18 empty (148.5 µL) + **Ru1** (1.5 µL), 2: *E. coli* MG1655 pSU18 empty (148.5 µL) + **Ru2** (1.5 µL), 3: *E. coli* MG1655 pSU18 empty (147 µL) + **Ru1** (1.5 µL) + HADA (1.5 µL), 4: *E. coli* MG1655 pSU18 empty (147 µL) + **Ru2** (1.5 µL) + HADA (1.5 µL), 5: *E. coli* MG1655 pSU18 NDM-1 (148.5 µL) + **Ru1** (1.5 µL), 6: *E. coli* MG1655 pSU18 NDM-1 (148.5 µL) + **Ru2** (1.5 µL), 7: *E. coli* MG1655 pSU18 NDM-1 (147 µL) + **Ru1** (1.5 µL) + HADA (1.5 µL), 8: *E. coli* MG1655 pSU18 NDM-1 (147 µL) + **Ru2** (1.5 µL) + HADA (1.5 µL). The resulting cultures were incubated at 37 °C with shaking (180 rpm) in darkness for 1 h before centrifugation at 13000 rcf for 5 min. The supernatant was removed and the pellets resuspended in 150 µL MHBII, centrifuged and resuspended twice more at 13000 rcf and 5 min. Supernatant was again removed and the pellets were resuspended in 100 µL paraformaldehyde in phosphate buffered saline (4%) the resuspended culture was kept at room temperature for 30 min. The samples were then subjected to centrifugation at 13000 rcf for 5 min. Supernatant removed. Samples were then stored at -20 °C until imaging. Prior to imaging the samples were resuspended in 20 µL SlowFade™ Gold Antifade Mountant (Thermofisher). Imaged on a Visitech-international VT-iSIM confocal microscope, 150 x oil objective. Channel 1 ex/em: 405/450 nm, Channel 2 ex/em: 445/680. Images were analysed using FIJI (ImageJ).

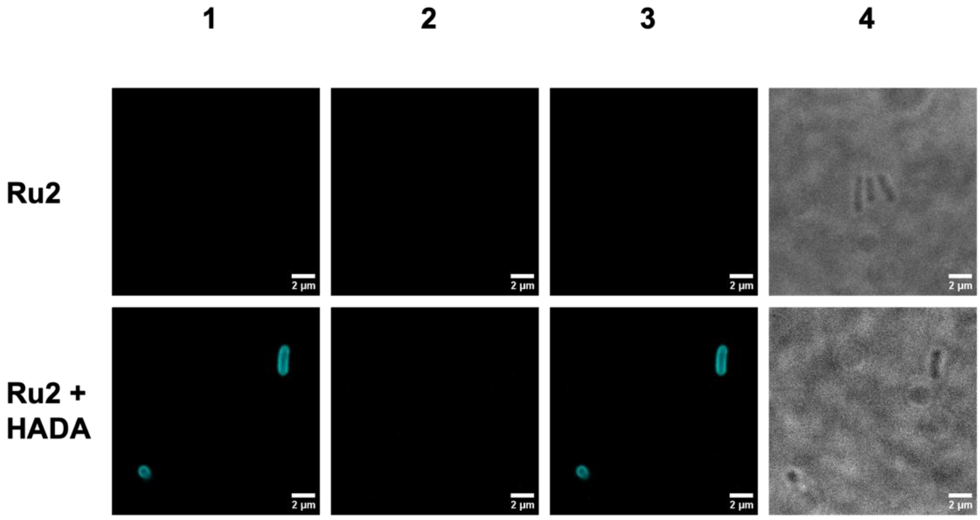

*Figure S35: E. coli MG1655 pSU18 NDM-1 treated with **Ru2** (100 µM), with or without HADA (100 µM). Suspended in SlowFade™ Gold Antifade Mountant (Thermofisher) Imaged on a Visitech-international VT-iSIM confocal microscope, 150 x oil objective. 1: ex/em: 405/450 nm, 2: ex/em: 445/680. 3: combination of 1 and 2, 4: brightfield. Images were analysed using FIJI (ImageJ).*

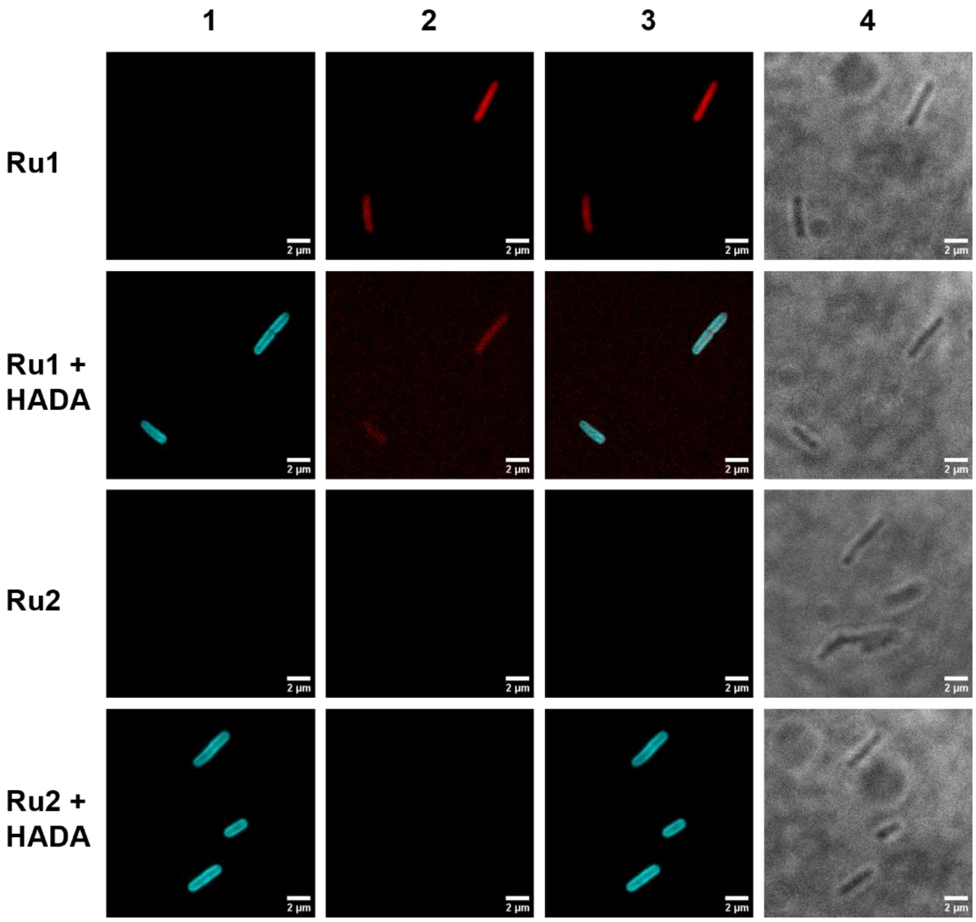

*Figure S36: E. coli MG1655 pSU18 empty treated with **Ru1** (100 µM), or **Ru2** (100 µM), with or without HADA (100 µM). Suspended in SlowFade™ Gold Antifade Mountant (Thermofisher) Imaged on a Visitech-international VT-iSIM confocal microscope, 150 x oil objective. 1: ex/em: 405/450 nm, 2: ex/em: 445/680. 3: combination of 1 and 2, 4: brightfield. Images were analysed using FIJI (ImageJ).*

1.12 LC-MS accumulation assay

1.12.1 Bacterial Strains and Growth Conditions:

Accumulation assay was performed two *E. coli* K-12 MG1655 strains transfected with pSU18 vector containing NDM-1 expression or an empty vector control. Cell growth was performed in Luria-Bertani (LB) broth (NaCl 10g/L, K_2HPO_4 6.3 g/L, KH_2PO_4 1.8 g/L, sodium citrate 0.45 g/L, $MgSO_4{\bullet}7H_2O$ 0.09 g/L, $(NH_4)_2SO_4$ 0.9 g/L, glycerol 44.0 g/L) at 37 °C with aeration.

1.12.2 Preparation of Accumulation Assay Samples for LC-MS Analysis:

Assay was adapted from the work of Geddes *et al.* and Widya *et al.*[9–11] *E. coli* MG1655 pSU18 NDM-1 and pSU18 Empty were grown from single colonies at 37 °C with aeration, while shaking at 180 rpm for 18 hours. The overnight suspensions were subcultured (1:100) in fresh LB and incubated to yield an OD_{600} of 0.55-0.60. The were harvested and pelleted at 3500 × g for 15 minutes at 4 °C. The pellets were washed with Meuller-Hinton II (MHII) broth and pelleted again under the same conditions. The pellets were resuspended in an adjusted volume of fresh MHII to achieve an $OD_{600} \approx 10.0$. Shielded from ambient light, cells were incubated with 320 µM of metal complex for 1 hour while shaking at 180 rpm. Separately, another set of cells was incubated under identical conditions without any added metal complex. Cells

incubated with metal complex were maintained incubating under the same conditions for an additional 10 minutes, while the second set of cells was incubated with 75 µM of meropenem trihydrate for 10 minutes. After treatment, 10 µL of each cell suspension was taken and dilution 1:10 PBSA for OD_{600} viability analysis. A 150 µL aliquot of each cell suspension was layered onto 150 µL of cold silicone oil mixture (1:9, v/v AR20:High temperature silicone oil) and centrifuged at 3500 × g for 1 hour at 4 °C. The aqueous and oil supernatant were carefully aspirated to isolate the cell pellets, which were resuspended in 50 µL of water and transferred to a fresh microplate. For lysis, a 1:1 (v/v) mixture of acetonitrile and methanol was added to each sample, and the samples were freeze thawed at -80 °C for approximately 18 hours. Thawed samples were clarified by two rounds of centrifugation at 3500 × g for 1 hour at 4 °C, each.

External calibration curves were prepared by mixing compound at increasing concentration with extracted cell lysate (prepared in parallel to analyte containing samples) in concentrations of 25, 50, 100, 200, 400, 800, 1600 and 3200 nM. Quality control samples of 1500, 300 and 50 nM were also prepared to assess quantitative accuracy. Samples were submitted to LCMS analysis as biological triplicate using a single calibration curve. Sulfadimethoxine at 1500 nM was spiked into each calibration and analyte sample as an internal standard.

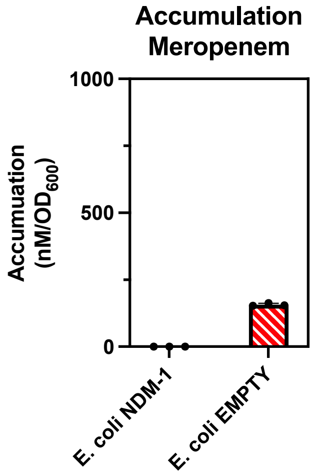

Figure S37: Accumulation of meropenem trihydrate (75 µM) in E. coli empty (0 nM/OD_{600}) and E. coli NDM-1 (150 nM/OD_{600}) strains.

1.12.3 LC-MS Analysis:

Chromatography was performed using a Waters AQCUITY Premier UPLC system, fitted with a CORTECS T3 (2.1 × 30 mm, 1.6 µm particle size and 120 Å pore) fitted with a VanGuard Pre-column (2.1 × 0.5 mm) of complimentary stationary phase, with a sample injection volume of 1.8 µL. Chromatographic conditions used water as non-polar solvent A and acetonitrile as polar solvent B, both supplemented with 0.1 % formic acid. The flowrate was set to 0.5 mL/min using the following gradient: 0 to 0.6 min (3% B), 0.6 to 2.1 min (3 – 95% B), 2.1 to 2.7 min (95% B), 2.7 to 2.76 min (95 – 3% B) and 2.76 – 3 min (3% B), with an additional 0.3 min for column equilibration. Samples were detected by a Waters XEVO G2-XS quadropole time-of-flight mass spectrometer via electrospray ionisation, operating in positive mode. Leucine-

enkephalin (m/z 556.2771) was injected at 200 pg/µL every 0.5 mins for lock mass calibration. Detection was performed using resolution mode, at continuum using a scan rate of 0.06 s.

1.12.4 Data and statistical analysis:

The integrated compound signals were collected, and regression analysis were performed using TargetLynx (Waters Corp) software. Single polynomial regression was performed, using a $1/x$ weighting excluding the calibration origin. Determined sample concentrations were normalised against viability OD_{600} readings to estimate compound accumulation per cell density. All assay-based screening experiments were performed in biological triplicate, each replicate starting from a new single colony inoculation. Statistical significance was determined using two-way ANOVA followed by Sidak's multiple comparison test, with a single pooled variance.

1.13 Checkerboard Broth Microdilution Minimum Inhibitory Concentration (MIC) assay

20% glycerol stocks of *E. coli* MG1655 pSU18 NDM-1 (*E. coli* NDM-1) and *E. coli* MG1655 pSU18 empty (*E. coli* empty) stored at -80 °C were used to streak nutrient agar plates. After incubation for 24 h at 37 °C, 12.5 mL Mueller Hinton Broth II (MHBII) in a 125 mL Erlenmeyer was inoculated with either *E. coli* strain and incubated at 37 °C with shaking at 180 rpm for 16 h. The MHBII culture growing both *E. coli* strains contained chloramphenicol as an antibiotic resistance marker (2 µg/mL, equivalent to ¼ $MIC_{E. coli}$ for chloramphenicol = 8 µg/mL). The resulting suspensions were each diluted 100 x (15 µL in 1.5 mL) in MHBII with chloramphenicol (2 µg/mL), and the resultant suspensions were incubated to OD_{600} = 0.6-1.2 (~3 h) at 37 °C with shaking.

To generate the two-dimensional drug interaction matrix, four 96-well source plates were prepared. In Source Plates A1-3, the three analytes were subjected to a two-fold serial dilution across the rows (A to G), maintaining constant concentrations within each row and achieving a vertical concentration gradient. In parallel, Source Plate B was prepared by diluting meropenem in a two-fold series across the Columns (9 to 2), generating a horizontal concentration gradient across the plate. Equal volumes (1 µL) from one of the source plates A and source plate B were transferred into a 96-well assay plate to establish the checkerboard layout with a 50X dilution into MHBII (50 µL total volume).

The prepared bacterial cells were diluted to OD_{600} = 0.01. Each prepared checkerboard plate was then inoculated with 50 µL per well of bacterial culture in MHBII with ½ MIC of chloramphenicol (100 µL total volume per well, final OD_{600} = 0.005). Control wells were included to account for drug-free growth, sterility, and single-agent gradient exposures. Dark plates were then incubated at 37 °C for 24 h before OD_{600} reading. For the light condition, the assays plates were statically incubated at 37 °C in the dark for one hour, followed by irradiation (450 nm, 60 J cm^{-2}). They were then further incubated at 37 °C for 23 h before OD_{600} reading. OD_{600} was measured on a BMG Labtech CLARIOstar plate reader. The MIC is the first value at which no growth is seen. All *E. coli* NDM-1 data were averaged from 3 biological replicates, while the *E. coli* empty strain data 1 replicate was used due to clear susceptibility towards meropenem at 0.32 µM with and without compound treatment.

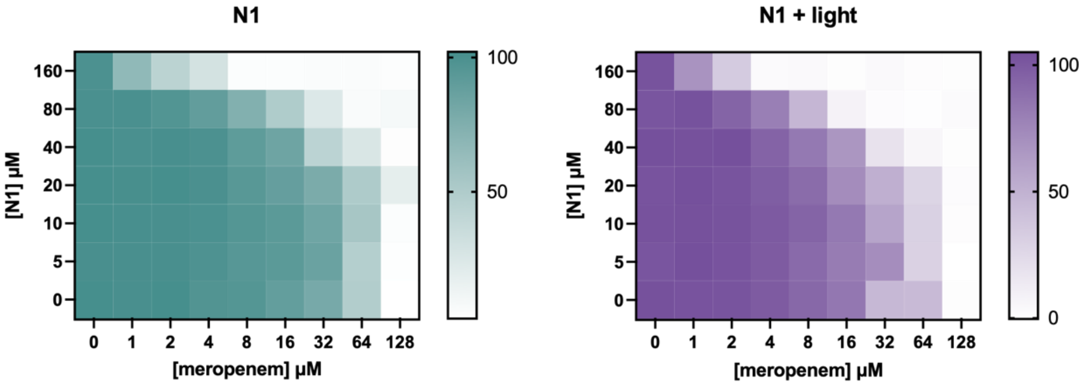

*Figure S38: Heat maps representing the checkerboard assay results for **N1** (0 – 160 µM) combined with meropenem (0 – 128 µM) in the dark and in the light (450 nm, 60 J cm^{-2}). Higher intensity of colour represents greater bacterial growth.*

1.14 Cell Viability Studies

Compound cytotoxicity was tested simultaneously on seven representative cell lines HaCat (human keratinocyte), HeLa (human cervical cancer), HCT116 (human colorectal carcinoma), hTERT RPE-1 (human retinal pigment epithelial), RAW264.7 (mouse macrophage), HepG2 (human liver cancer), HEK293T (human embryonic kidney), were incubated with the compound for 24 or 48 h at 37 °C and 5% CO_2 (v/v). The testing concentration range for each compound was made of 8 points, following a 3-fold dilution, starting from 50 µM for **Ru1**, **Ru2** and **N1**. The required amount of compound was distributed in each well of the 384-well assay plate (Bio-one, Greiner CELLSTAR® microplates) using the Echo 650 Acoustic Liquid Handler and wells were back-filled with DMSO when required to keep a final DMSO concentration of 0.5 %. Next, 50 µL of cell solution was added to the compound-containing well using the Integra® Viafill Bulk dispenser. Cell solutions were prepared to get 5000 (24hr)/3500 (48hr) cells per well for HaCaT, 5000 (24hr)/2500 (48hr) cells per well for HeLa, 10 000 (24hr)/5000 (48hr) cells per well for HCT116, 5000 (24hr)/1500 (48hr) cells per well for hTERT RPE-1, 15 000 (24hr)/8000 (48hr) cells per well for RAW264.7, 5000 (24hr)/ 3500 (48hr) cells per well for HepG2, and 3000 (24hr)/ 1500 (48hr) cells per well for HEK293T. After incubation, cells were fixed with 4% PFA (Merck, F8775), permeabilised with 0.2% Triton X-100 (Merck, T8787) and stained to visualise their nuclei using 2 µg/mL DAPI (Merck, 10236276001). Viable cells were acquired at 377 nm excitation and 447 nm emission filter using the CeligoTM Image Cytometer. The number of nuclei per well was determined using the Direct Cell Count analysis from the CeligoPro Sofware (5.5.1.0). All conditions were performed in triplicate. Staurosporine (Cambridge Bioscience, s7600) was used as a positive control and DMSO-only as a negative control. The percentage of cell viability was normalised to the DMSO-only control and LD_{50}s were extracted with MatLab using a 4-parameter logistic curve.

*Table S6: Cytotoxicity data for compounds **N1**, **Ru1**, **Ru2** and staurosporine (positive control) represented as LD_{50} values (µM). Values represent the mean of biological triplicate.*

	HCT116		HEK293T		HaCaT		HeLa		HepG2		RAW		RPE1	
	24 h	48 h	24 h	48 h	24 h	48 h	24 h	48 h	24 h	48 h	24 h	48 h	24 h	48 h
**N1**	>50	>50	>50	>50	>50	>50	>50	>50	>50	>50	>50	>50	>50	>50
**Ru1**	>50	>50	>50	>50	>50	>50	>50	>50	>50	>50	>50	>50	>50	>50
**Ru2**	>50	>50	>50	>50	>50	>50	>50	>50	>50	>50	>50	>50	>50	>50
**Staurosporine**	0.08	0.02	0.07	0.01	0.05	0.01	0.05	0.02	0.3	0.09	0.03	0.01	0.01	0.01

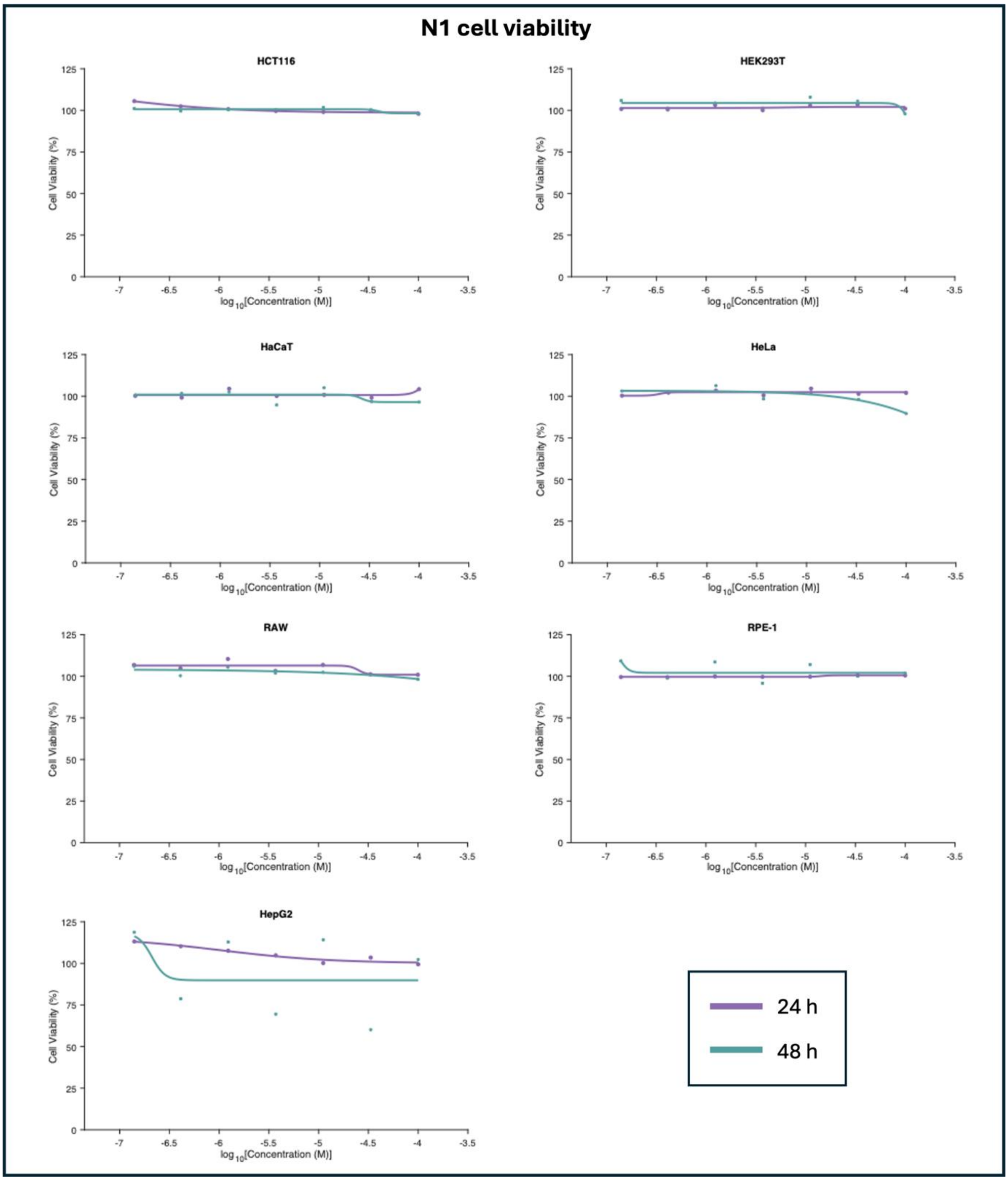

*Figure S39:Cell viability dose response curves for **N1** against seven tested cell lines (24 and 48 h): HCT116, HEK293T, HaCaT, HeLa, RAW, RPE1 and HepG2.*

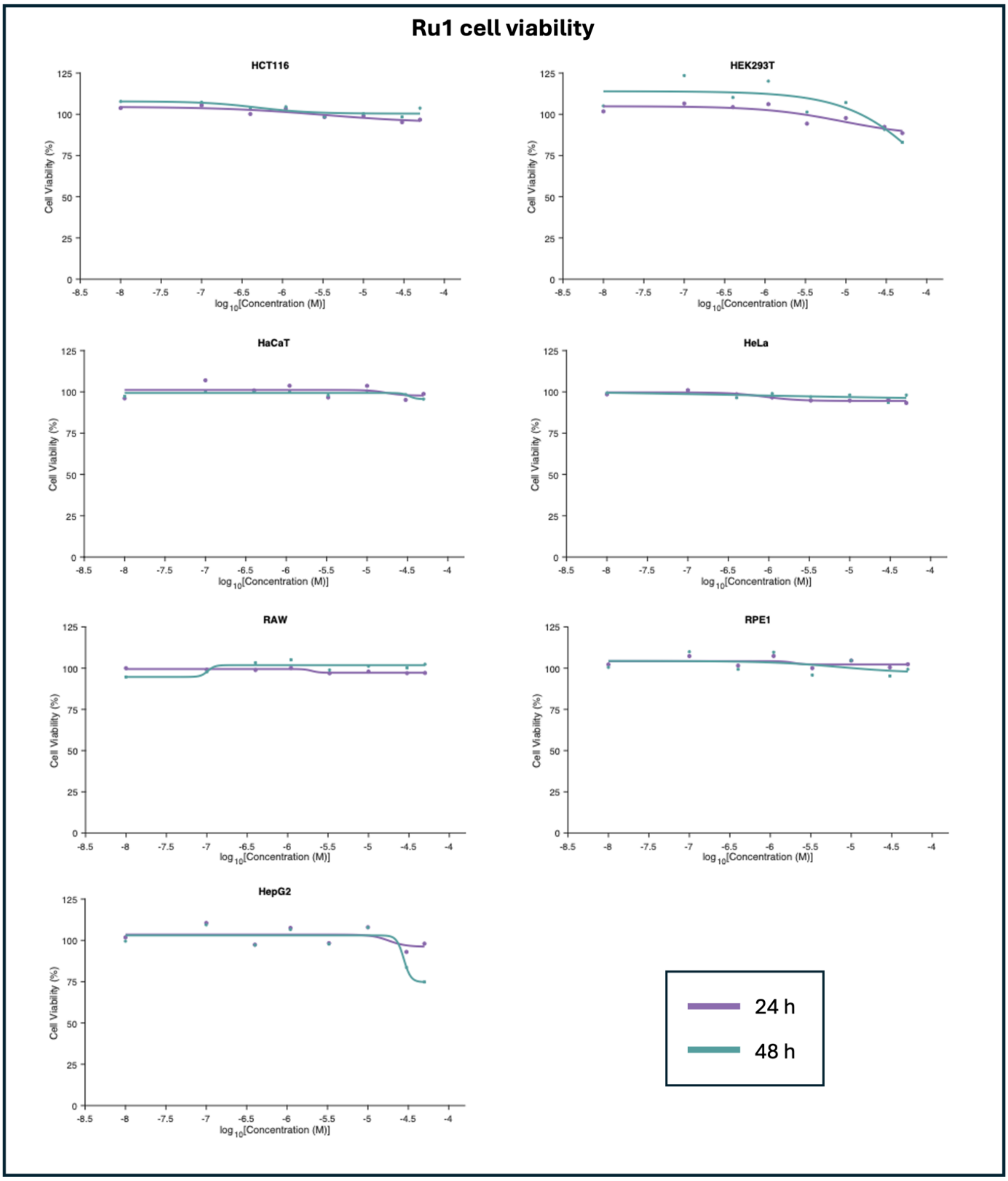

*Figure S40: Cell viability dose response curves for **Ru1** against seven tested cell lines (24 and 48 h): HCT116, HEK293T, HaCaT, HeLa, RAW, RPE1 and HepG2.*

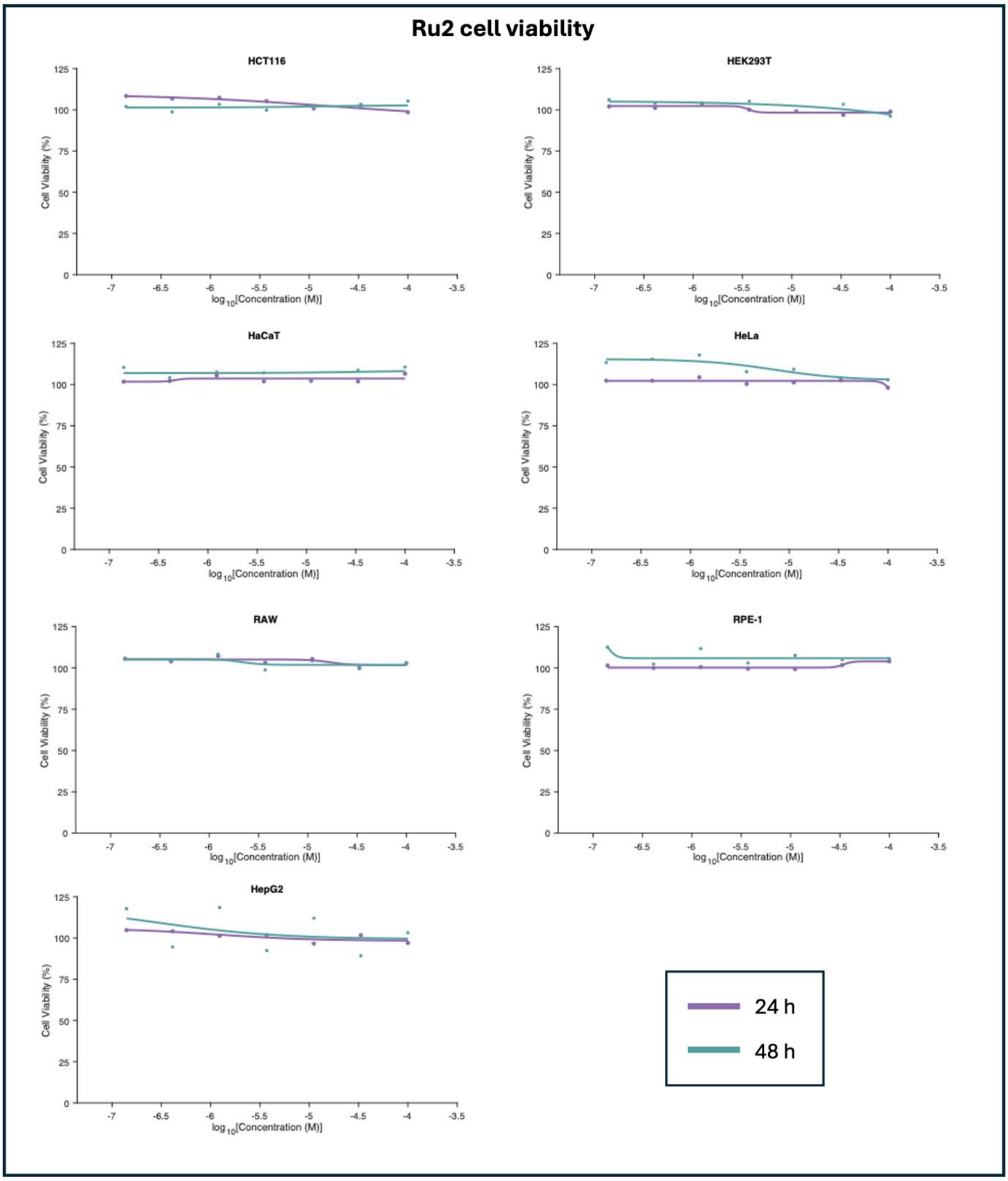

Figure S41: Cell viability dose response curves for ***Ru2*** *against seven tested cell lines (24 and 48 h): HCT116, HEK293T, HaCaT, HeLa, RAW, RPE1 and HepG2.*

1.15 NMR spectra

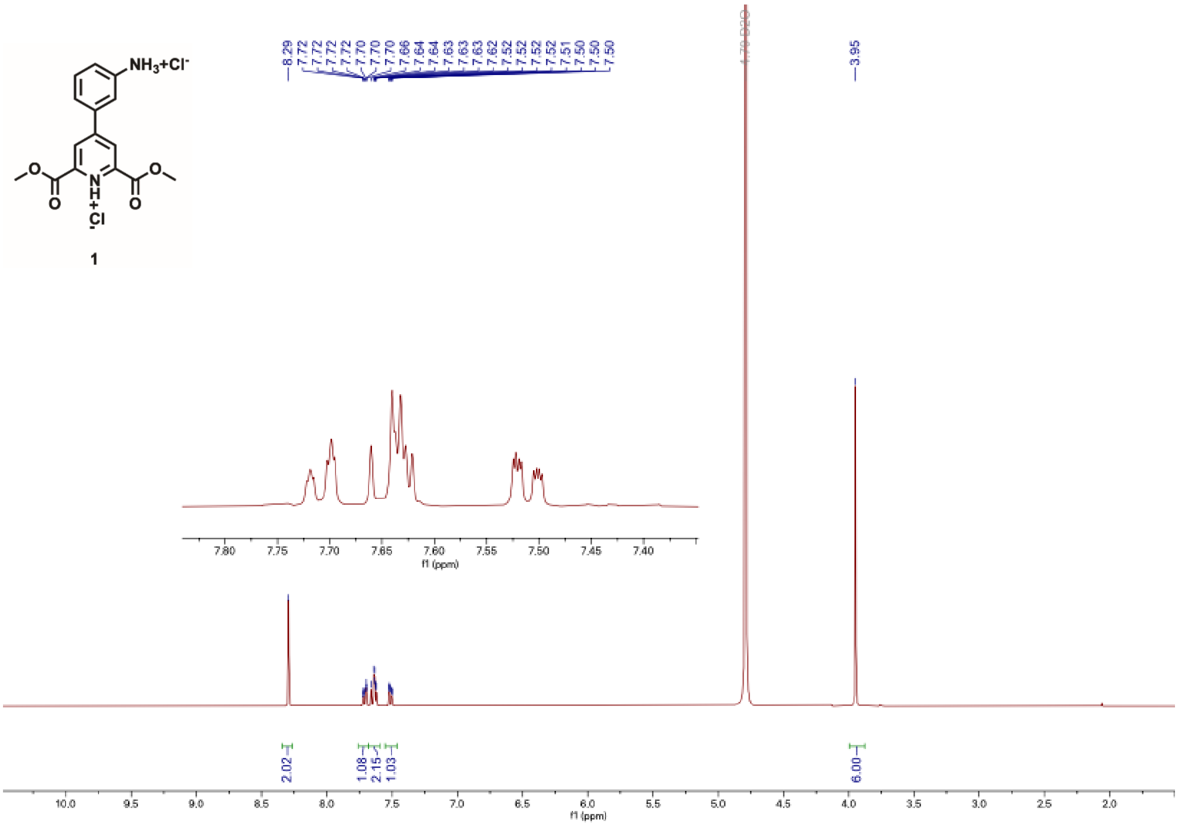

Figure S42: [1]*H NMR (400 MHz, 25 °C, D_2O) spectrum of* ***1***.

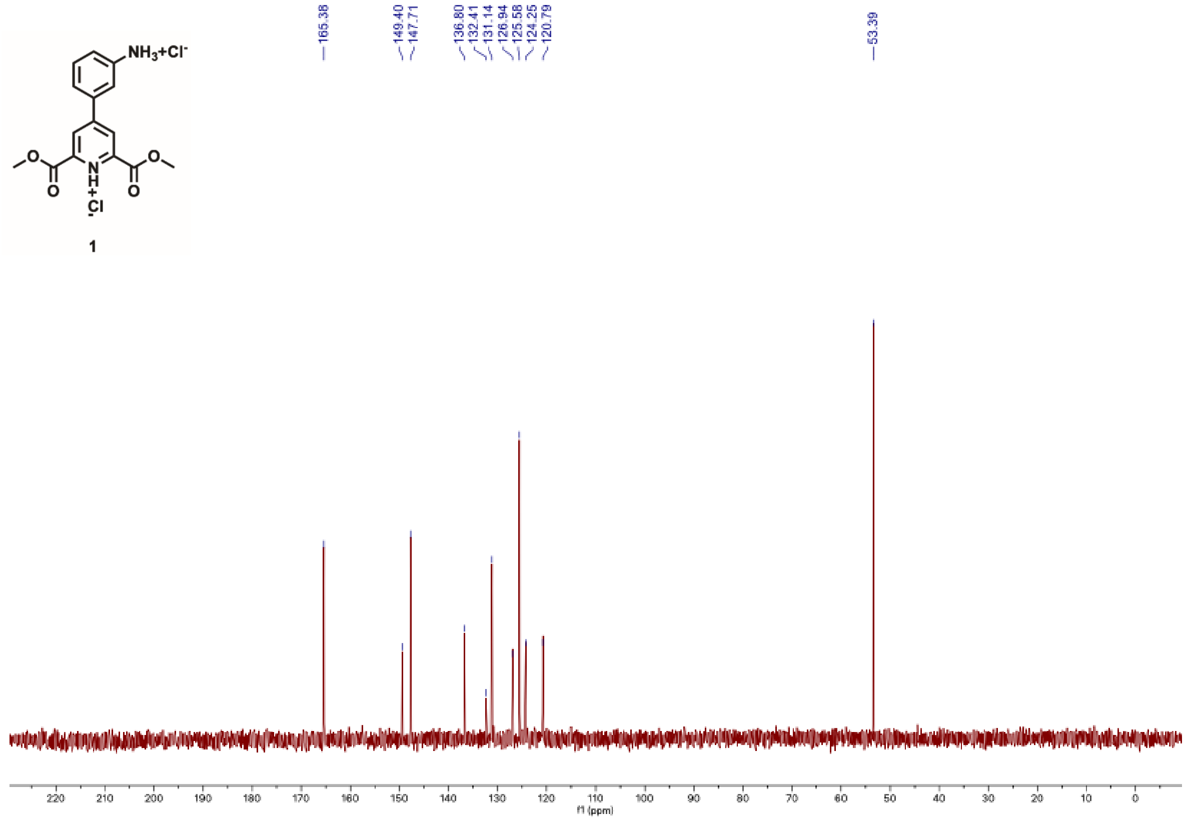

Figure S43: [13]*C NMR (101 MHz, 25 °C, D_2O) spectrum of* ***1***.

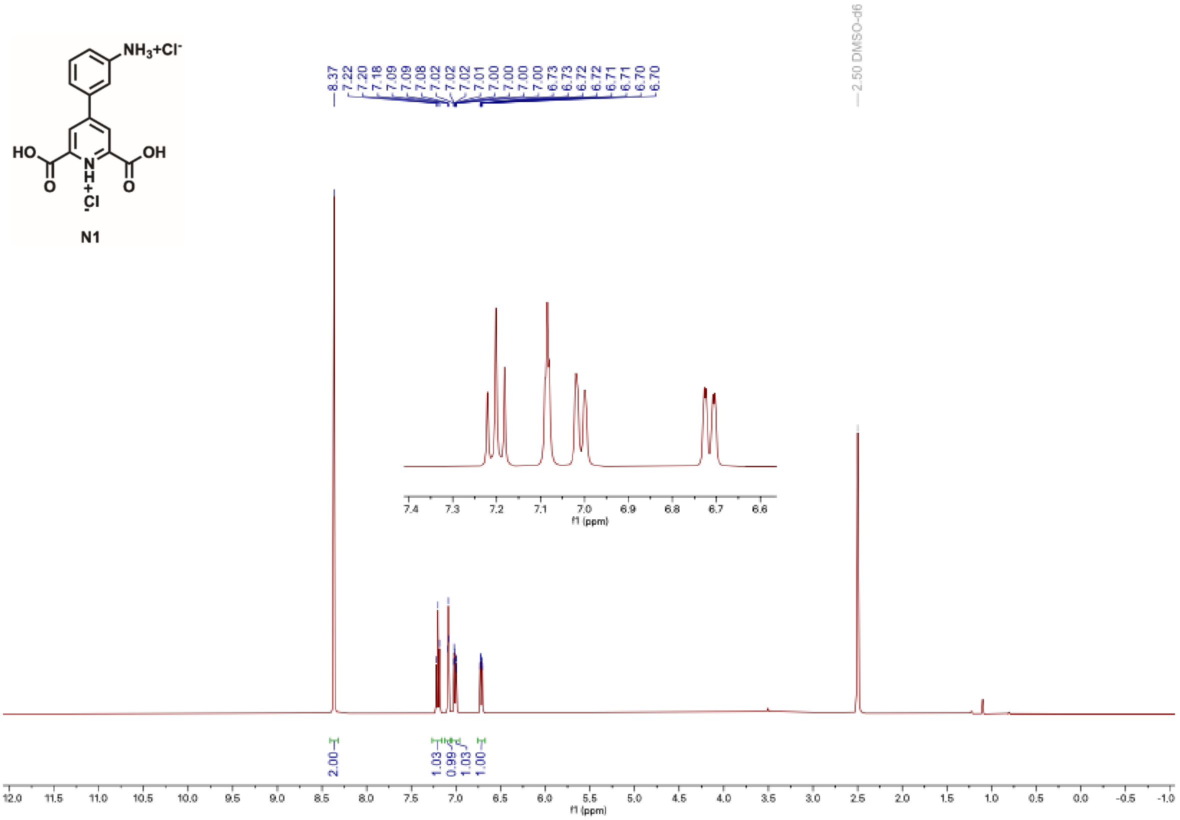

Figure S44: ^{1}H NMR (400 MHz, 25 °C, DMSO-d6) spectrum of **N1**.

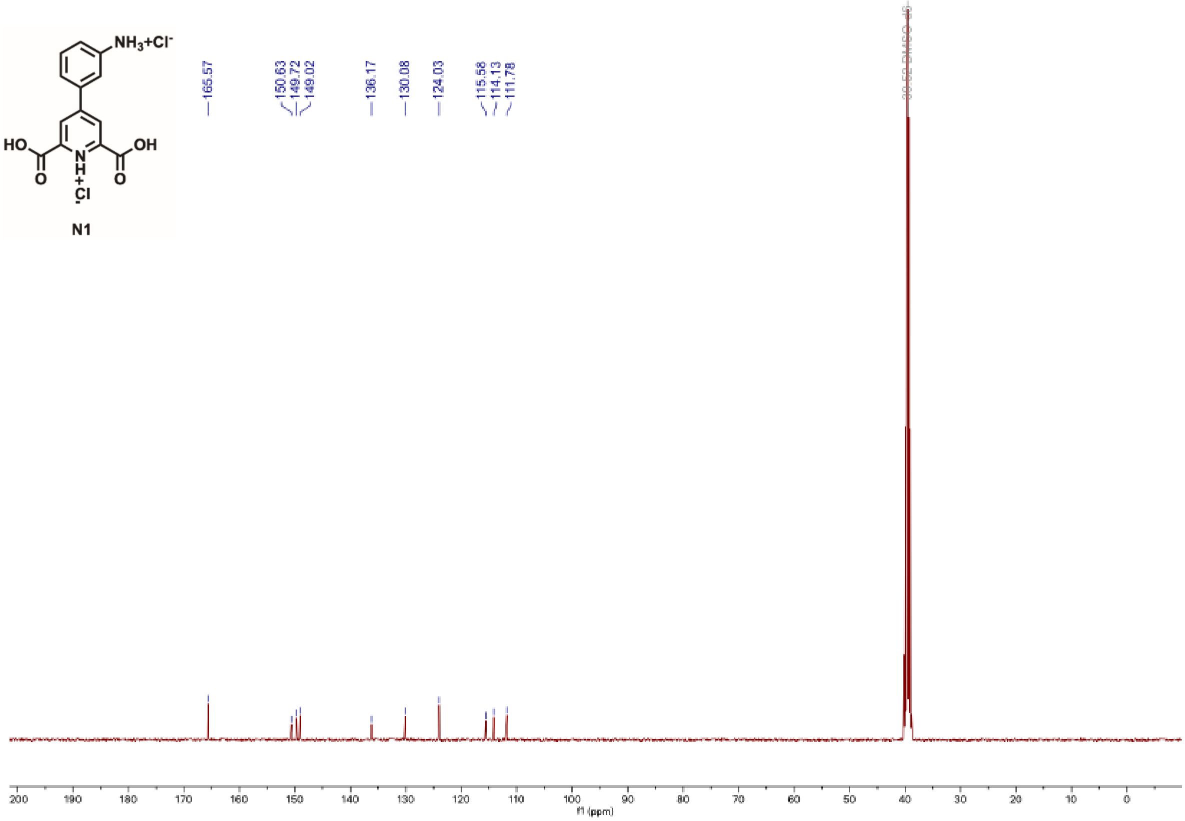

Figure S45: ^{13}C NMR (101 MHz, 25 °C, DMSO-d6) spectrum of **N1**.

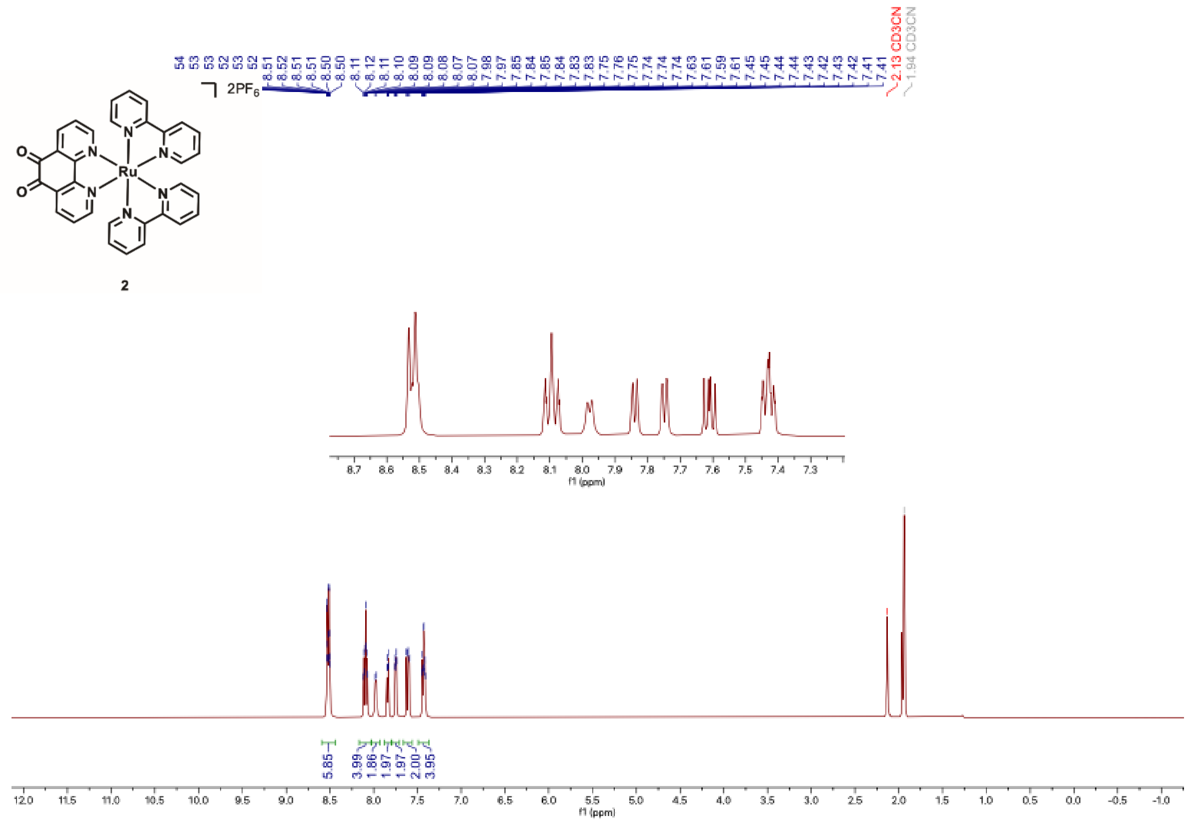

*Figure S46: ^{1}H NMR (400 MHz, 25 °C, CD_3CN) spectrum of **2**.*

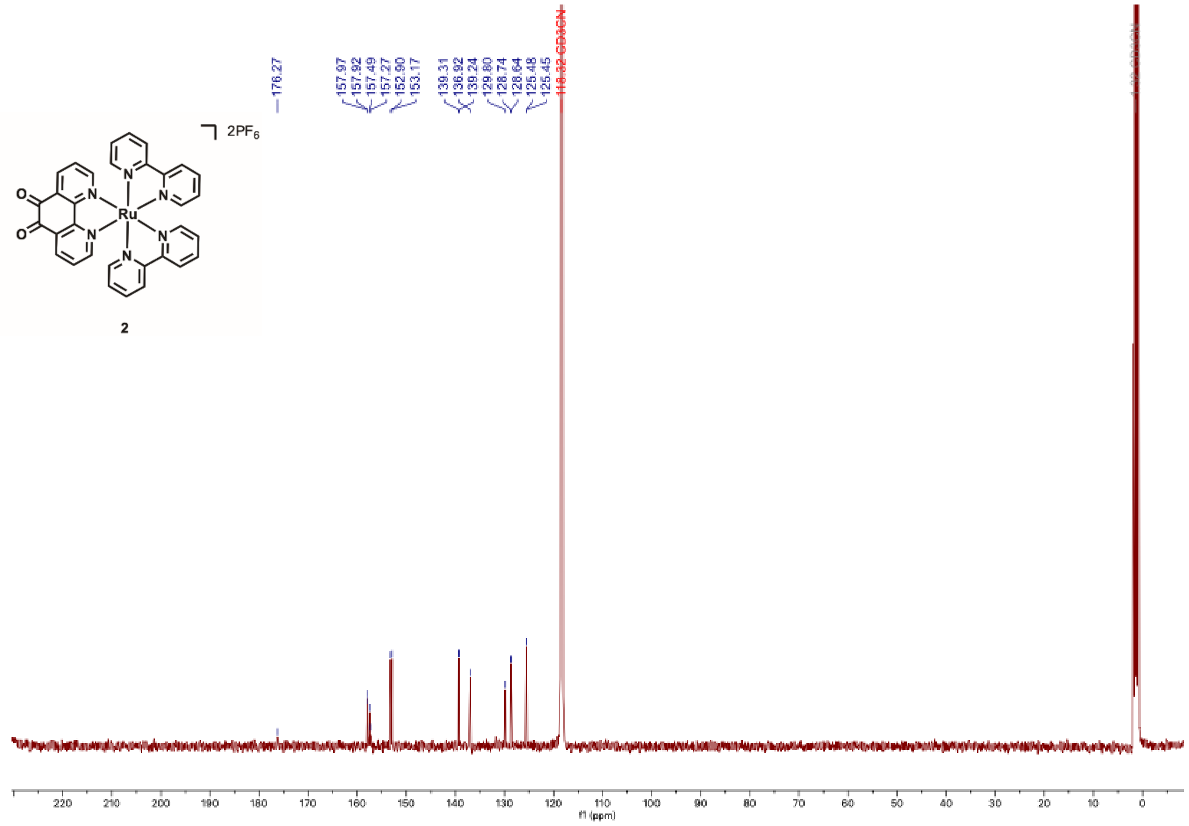

*Figure S47: ^{13}C NMR (101 MHz, 25 °C, CD_3CN) spectrum of **2**.*

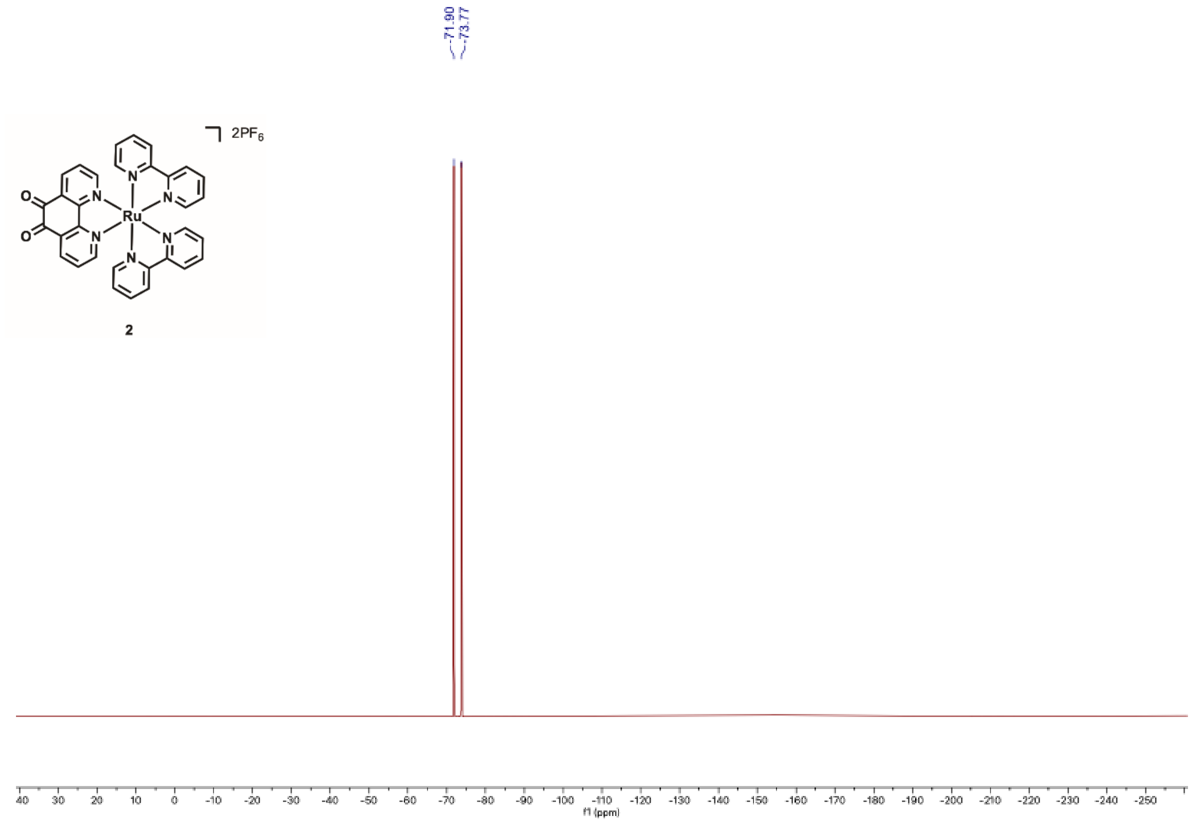

*Figure S48: ^{19}F NMR (376 MHz, 25 °C, CD_3CN) spectrum of **2**.*

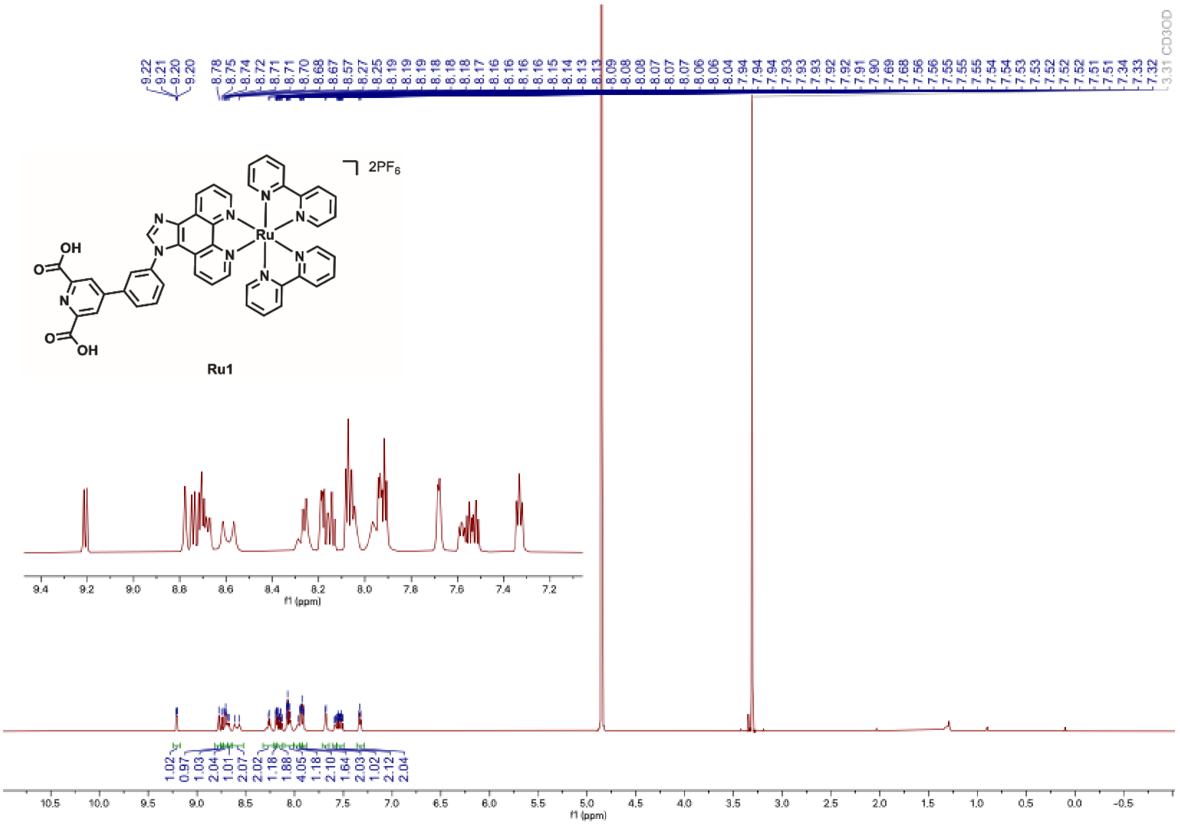

*Figure S49: ^{1}H NMR (600 MHz, 25 °C, MeOD) spectrum of **Ru1**.*

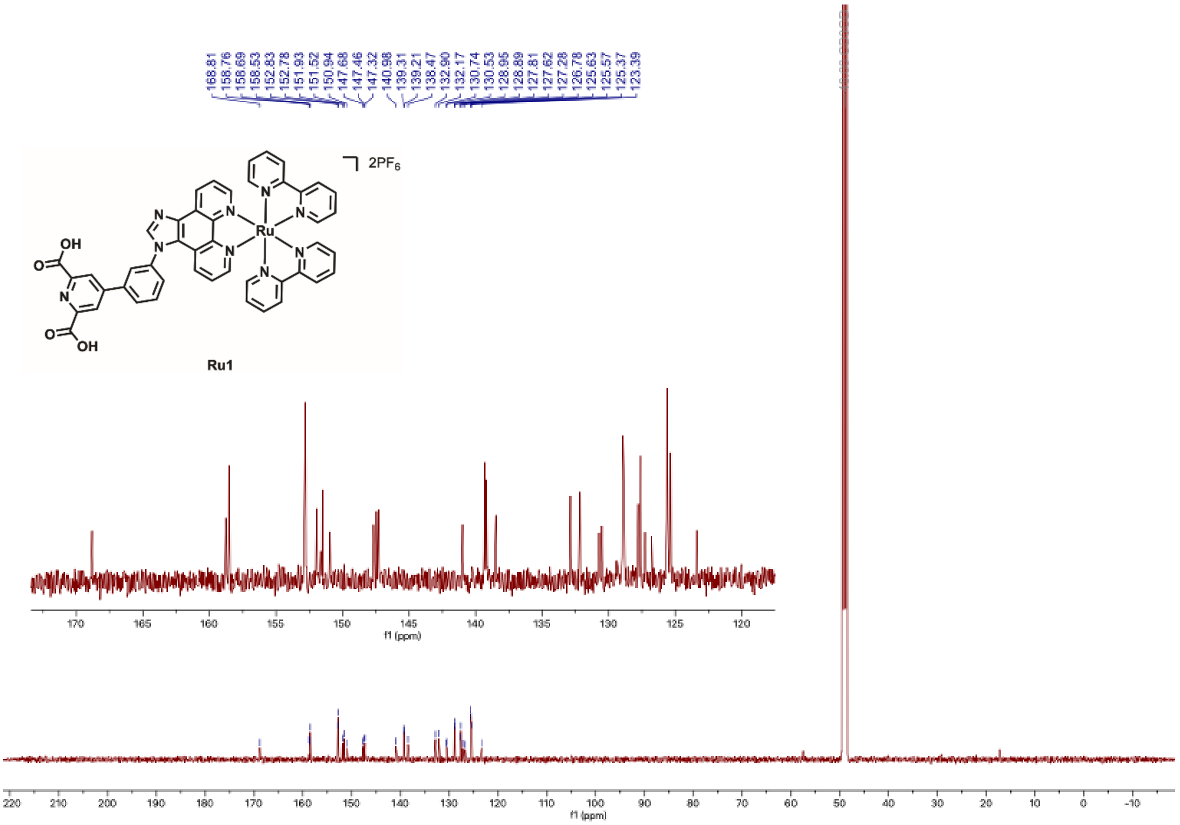

*Figure S50: ^{13}C NMR (151 MHz, 25 °C, MeOD) spectrum of **Ru1**.*

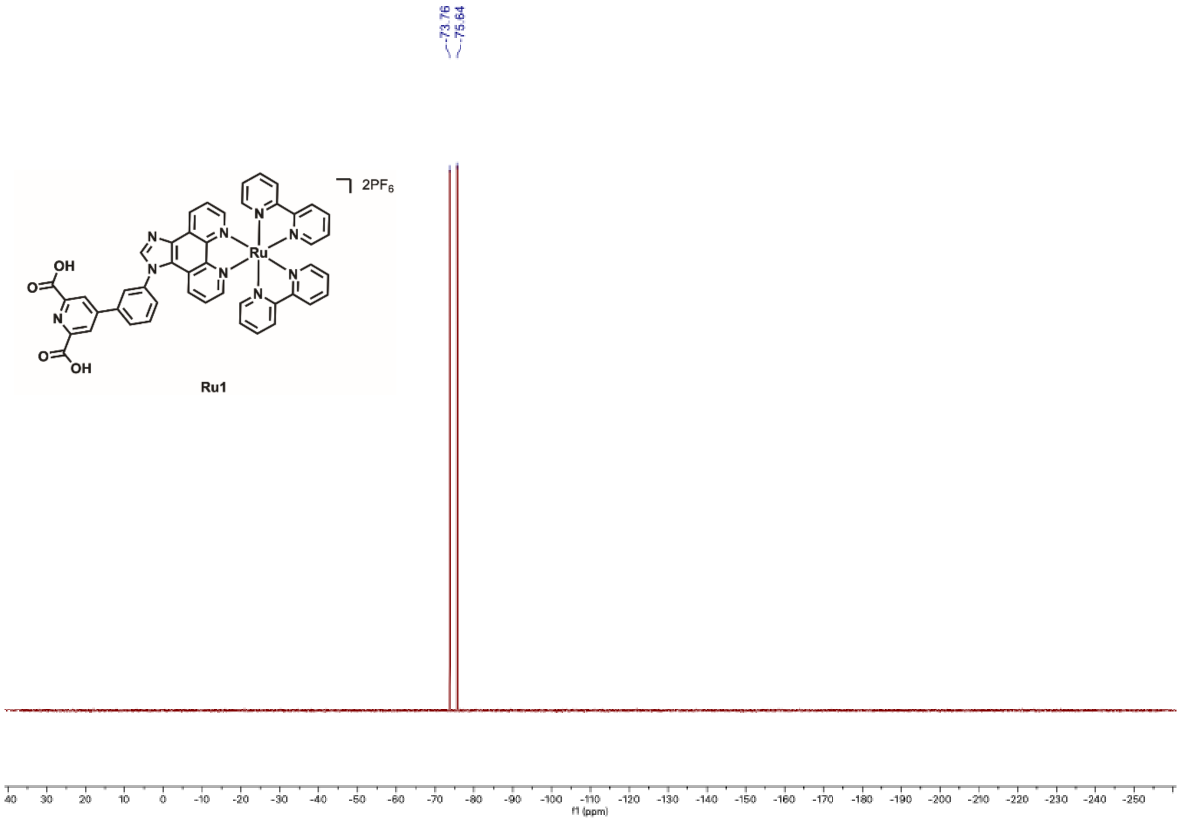

*Figure S51: ^{19}F NMR (376 MHz, 25 °C, MeOD) spectrum of **Ru1**.*

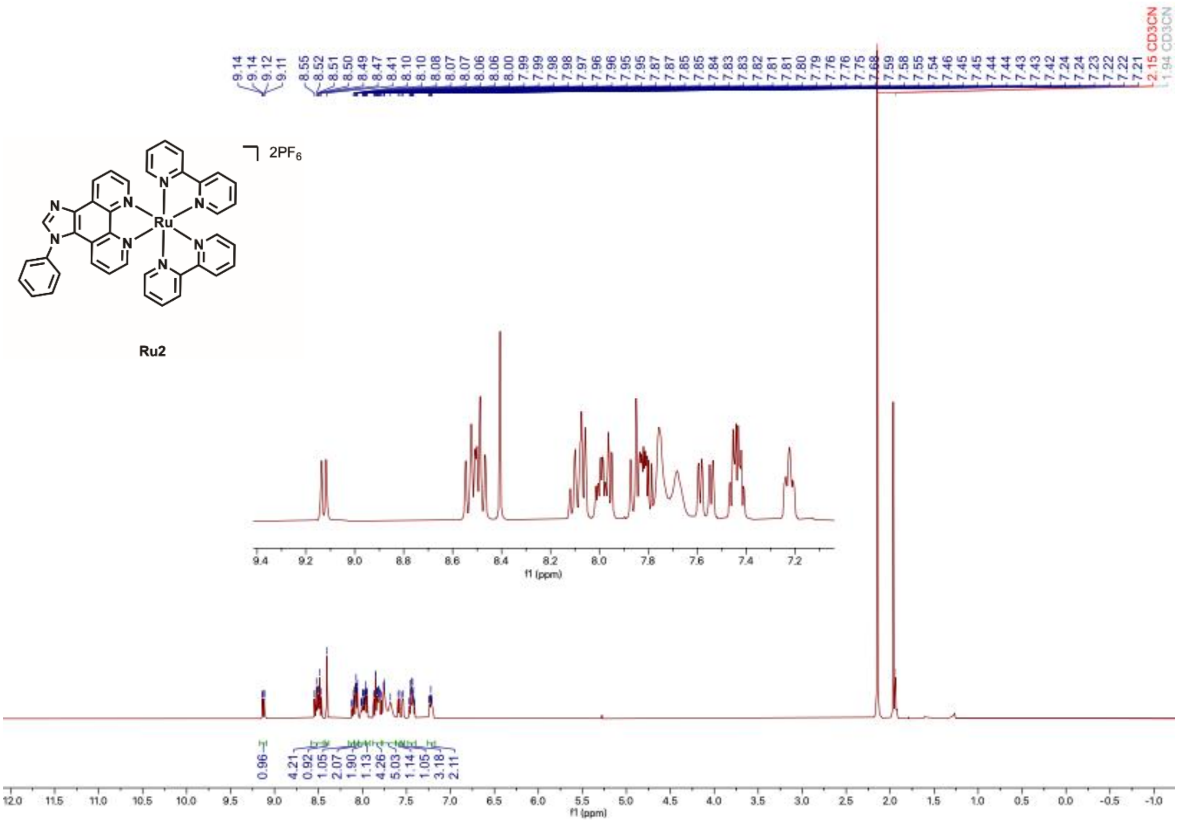

*Figure S52: ^{1}H NMR (400 MHz, 25 °C, CD$_3$CN) spectrum of **Ru2**.*

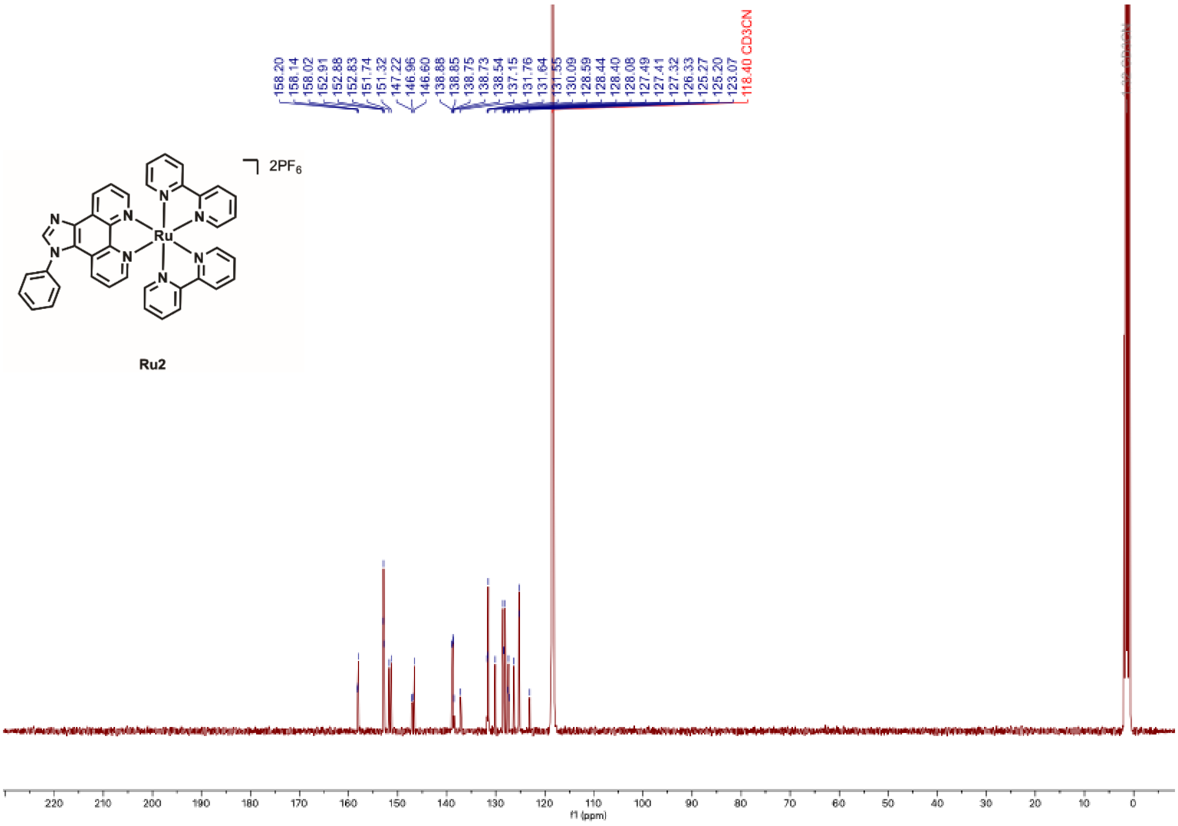

*Figure S53: ^{13}C NMR (101 MHz, 25 °C, CD$_3$CN) spectrum of **Ru2**.*

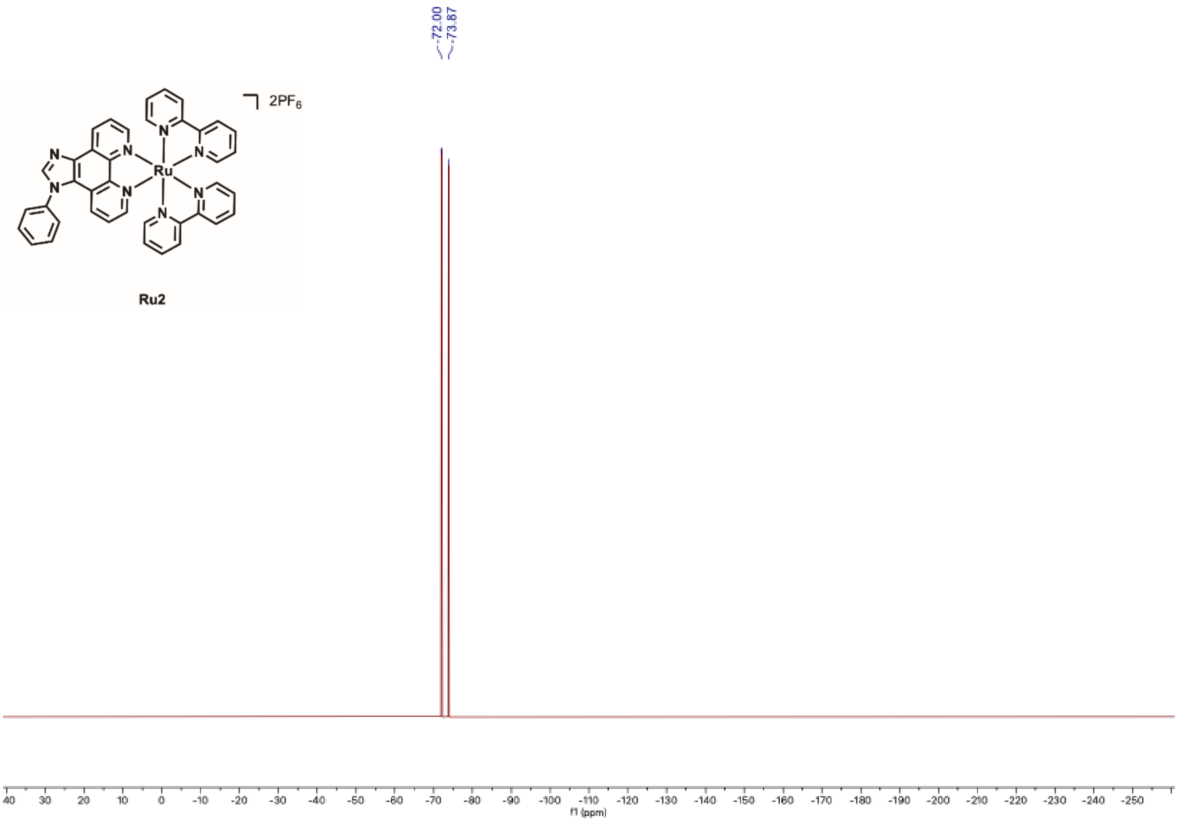

Figure S54: : ^{19}F NMR (376 MHz, 25 °C, CD_3CN) spectrum of ***Ru2****.*

1.16 References

(1) Chen, A. Y.; Thomas, P. W.; Stewart, A. C.; Bergstrom, A.; Cheng, Z.; Miller, C.; Bethel, C. R.; Marshall, S. H.; Credille, C. V.; Riley, C. L.; Page, R. C.; Bonomo, R. A.; Crowder, M. W.; Tierney, D. L.; Fast, W.; Cohen, S. M. Dipicolinic Acid Derivatives as Inhibitors of New Delhi Metallo-β-Lactamase-1. *J. Med. Chem.* **2017**, *60* (17), 7267–7283. https://doi.org/10.1021/acs.jmedchem.7b00407.

(2) Wachter, E.; Moyá, D.; Parkin, S.; Glazer, E. C. Ruthenium Complex “Light Switches” That Are Selective for Different G-Quadruplex Structures. *Chem. – Eur. J.* **2016**, *22* (2), 550–559. https://doi.org/10.1002/chem.201503203.

(3) Valkó, K.; Bevan, C.; Reynolds, D. Chromatographic Hydrophobicity Index by Fast-Gradient RP-HPLC: A High-Throughput Alternative to Log P/Log D. *Anal. Chem.* **1997**, *69* (11), 2022–2029. https://doi.org/10.1021/ac961242d.

(4) Tanielian, C.; Wolff, C.; Esch, M. Singlet Oxygen Production in Water: Aggregation and Charge-Transfer Effects. *J. Phys. Chem.* **1996**, *100* (16), 6555–6560. https://doi.org/10.1021/jp952107s.

(5) Shapiro, A. B. Kinetics of Sulbactam Hydrolysis by β-Lactamases, and Kinetics of β-Lactamase Inhibition by Sulbactam. *Antimicrob. Agents Chemother.* **2017**, *61* (12), 10.1128/aac.01612-17. https://doi.org/10.1128/aac.01612-17.

(6) Fic, E.; Kedracka-Krok, S.; Jankowska, U.; Pirog, A.; Dziedzicka-Wasylewska, M. Comparison of Protein Precipitation Methods for Various Rat Brain Structures Prior

to Proteomic Analysis. *ELECTROPHORESIS* **2010**, *31* (21), 3573–3579. https://doi.org/10.1002/elps.201000197.

(7) Perkins, D. N.; Pappin, D. J. C.; Creasy, D. M.; Cottrell, J. S. Probability-Based Protein Identification by Searching Sequence Databases Using Mass Spectrometry Data. *ELECTROPHORESIS* **1999**, *20* (18), 3551–3567. https://doi.org/10.1002/(SICI)1522-2683(19991201)20:18<3551::AID-ELPS3551>3.0.CO;2-2.

(8) Hakkennes, M. L. A.; Buda, F.; Bonnet, S. MetalDock: An Open Access Docking Tool for Easy and Reproducible Docking of Metal Complexes. *J. Chem. Inf. Model.* **2023**, *63* (24), 7816–7825. https://doi.org/10.1021/acs.jcim.3c01582.

(9) Geddes, E. J.; Gugger, M. K.; Garcia, A.; Chavez, M. G.; Lee, M. R.; Perlmutter, S. J.; Bieniossek, C.; Guasch, L.; Hergenrother, P. J. Porin-Independent Accumulation in Pseudomonas Enables Antibiotic Discovery. *Nature* **2023**, *624* (7990), 145–153. https://doi.org/10.1038/s41586-023-06760-8.

(10) Geddes, E. J.; Li, Z.; Hergenrother, P. J. An LC-MS/MS Assay and Complementary Web-Based Tool to Quantify and Predict Compound Accumulation in E. Coli. *Nat. Protoc.* **2021**, *16* (10), 4833–4854. https://doi.org/10.1038/s41596-021-00598-y.

(11) Widya, M.; Pasutti, W. D.; Sachdeva, M.; Simmons, R. L.; Tamrakar, P.; Krucker, T.; Six, D. A. Development and Optimization of a Higher-Throughput Bacterial Compound Accumulation Assay. *ACS Infect. Dis.* **2019**, *5* (3), 394–405. https://doi.org/10.1021/acsinfecdis.8b00299.
